# Inequalities in the impact of COVID-19-associated disruptions on tuberculosis diagnosis by age and sex in 45 high TB burden countries

**DOI:** 10.1186/s12916-022-02624-6

**Published:** 2022-11-14

**Authors:** C. Finn McQuaid, Marc Y. R. Henrion, Rachael M. Burke, Peter MacPherson, Rebecca Nzawa-Soko, Katherine C. Horton

**Affiliations:** 1 TB Modelling Group, TB Centre and Centre for Mathematical Modelling of Infectious Diseases, Department of Infectious Disease Epidemiology, Faculty of Epidemiology and Population Health, London School of Hygiene & Tropical Medicine, London, UK; 2 Malawi-Liverpool-Wellcome Programme, Blantyre, Malawi; 3 Department of Clinical Sciences, Liverpool School of Tropical Medicine, Liverpool, UK; 4 Clinical Research Department, Faculty of Infectious and Tropical Diseases, London School of Hygiene & Tropical Medicine, London, UK

**Keywords:** Tuberculosis, Epidemiology, COVID-19, Gender, Notifications, Paediatric, Children, Elderly, Age, Sex

## Abstract

**Background:**

Tuberculosis remains a major public health priority and is the second leading cause of mortality from infectious disease worldwide. TB case detection rates are unacceptably low for men, the elderly and children. Disruptions in TB services due to the COVID-19 pandemic may have exacerbated these and other inequalities.

**Methods:**

We modelled trends in age- and sex- disaggregated case notifications for all forms of new and relapse TB reported to the World Health Organization for 45 high TB, TB/HIV and MDR-TB burden countries from 2013 to 2019. We compared trend predicted notifications to observed notifications in 2020 to estimate the number of people with TB likely to have missed or delayed diagnosis. We estimated the risk ratio (RR) of missed or delayed TB diagnosis for children (aged < 15 years) or the elderly (aged ≥ 65 years) compared to adults (aged 15–64 years) and women compared to men (both aged ≥ 15 years) using a random-effects meta-analysis.

**Results:**

An estimated 195,449 children (95% confidence interval, CI: 189,673–201,562, 37.8% of an expected 517,168), 1,126,133 adults (CI: 1,107,146–1,145,704, 21.8% of an expected 5,170,592) and 235,402 elderly (CI: 228,108–243,202, 28.5% of an expected 826,563) had a missed or delayed TB diagnosis in 2020. This included 511,546 women (CI: 499,623–523,869, 22.7%, of an expected 2,250,097) and 863,916 men (CI: 847,591–880,515, 23.0% of an expected 3,763,363). There was no evidence globally that the risk of having TB diagnosis missed or delayed was different for children and adults (RR: 1.09, CI: 0.41–2.91), the elderly and adults (RR: 1.40, CI: 0.62–3.16) or men and women (RR: 0.59, CI: 0.25–1.42). However, there was evidence of disparities in risk by age and/or sex in some WHO regions and in most countries.

**Conclusions:**

There is no evidence at an aggregate global level of any difference by age or sex in the risk of disruption to TB diagnosis as a result of the COVID-19 pandemic. However, in many countries, disruptions in TB services have been greater for some groups than others. It is important to recognise these context-specific inequalities when prioritising key populations for catch-up campaigns.

**Supplementary Information:**

The online version contains supplementary material available at 10.1186/s12916-022-02624-6.

## Background

Tuberculosis (TB) remains a major public health priority and, prior to the emergence of COVID-19, was the leading infectious cause of mortality worldwide [1]. Large inequalities exist in TB burden and access to care globally [2]. Older people are at higher risk of developing active TB disease [3], whilst children face high rates of mortality due to TB and have particularly low rates of case detection [1, 4]. TB prevalence is high amongst men [5, 6], who, compared to women, have more limited access to care [1], and in some settings may also be at an increased risk of drug-resistance [7]. Many of these inequalities have remained consistent over the last decade or more [2].

The COVID-19 pandemic has substantially impacted TB prevention, diagnosis, and care, resulting in major reductions in TB notifications, the number of people diagnosed with and reported as having TB, worldwide [1, 8, 9]. With a wide range of disruptions caused by the COVID-19 pandemic, we might expect that the impact of these disruptions on TB burden would vary for different population groups. For example, during the first three months of the COVID-19 pandemic in the UK, children and young people have had a much greater reduction in visits to accident and emergency departments for infections than the rest of the population [10], whilst reductions in clinic attendance for acute respiratory infections in Singapore were higher for both children and older adults [11]. Elderly people may have been more inclined to “shield” during the pandemic compared to working age adults and so be less likely to attend healthcare facilities. In Malawi, TB diagnosis in women and girls was significantly more affected than in men and boys in 2020 [12], whilst in India, more women than men were diagnosed in 2020–2021, in a reversal of previous trends [13]. In Mozambique, TB notifications amongst men were more severely impacted than amongst women [14]. In addition, the proportion of transmission attributable to contact within the household is likely to have increased as a result of COVID-19-associated non-pharmaceutical interventions [8], which may disproportionately affect children, who are at a high risk of infection when exposed to TB within the household [15]. However, efforts to estimate changes in the burden of TB as a result of COVID-19-associated disruptions going forward [1, 16, 17] have primarily focused on the overall effect of these disruptions with no consideration of potential inequalities in that effect.

Assessing disparities in the impact of the COVID-19 pandemic on TB services requires identifying any inequalities in access to TB diagnosis and then prioritising key populations for catch-up campaigns. In this analysis, we investigate the impact of COVID-19 on missed or delayed TB diagnosis in 2020 by age and sex for 45 high TB, TB/HIV and multidrug- or rifampicin-resistant (MDR) TB burden countries.

## Methods

### Data

We used country-level age- and sex-disaggregated data on new and relapse (new only in Azerbaijan) case notifications collected by national TB programmes and reported to the World Health Organization (WHO) [18]. These data recorded the number of TB patients who were notified to the healthcare system for the years 2013–2020 (the definitions for recording notifications by age and sex changed in 2013). We restricted our analysis to the WHO high TB, high HIV/TB and high MDR-TB burden countries [19]. Further details on total monthly notifications during 2020 can be found in [1].

We combined the reported five year age bands into: children (aged < 15 years), adults (aged 15–64 years) and the elderly (aged ≥ 65 years) of both sexes. We also considered men (aged ≥ 15 years) and women (aged ≥ 15 years). We did not consider boys (aged < 15 years) and girls (aged < 15 years) separately, as there is no evidence for a difference in TB burden between these populations [20], and the number of reported notifications was low. We only used notifications that were reported by age and sex, noting that these did not necessarily equate to the total number of notifications, as in some instances age and/or sex was not recorded. However, we confirmed that the proportion of notifications that included information on age and sex did not change over time.

### Analysis

We fitted Poisson regression models to data from 2013–2019 to estimate the expected number of notifications in 2020 in each country by age and by sex. Given that for each country, age- and sex-stratum there are only a maximum of seven data points, this Poisson model included only time t as a predictor variable. Mathematically, the model for *Y*_c,a,s_, the number of notifications in country *c* for age band *a* and sex *s* is given by *Y*_*c,a,s*_*|t* ~ Pois(λ_*c,a,*s_(*t*)) where *λ*_*c,a,s*_(*t*) = *E*[*Y*_*c,a,s*_|*t*] and log(*E*[*Y*_*c,a,*s_|*t*]) = *β*_0_ + *β*_1_∙*t* with *β*_0_ and *β*_1_ the regression parameters to be estimated. Further details can be found in Additional file 1.

To identify “missed or delayed” notifications (expected notifications minus observed notifications), we compared the model-predicted estimate for 2020 to the actual observed number of notifications. We used this to calculate the risk of not being diagnosed due to COVID-19-related disruptions (as opposed to the overall risk of not being diagnosed) for different populations. For example, for women the probability of being missed or delayed due to the COVID-19 pandemic was given by

$$\textit{P}\text{(}\textit{missed|COVID,women}\text{)=}\frac{\text{e}{\textit{xpected}}_\textit{women}{\textit{-observed}}_\textit{women}}{{\textit{expected}}_\textit{women}}$$

The risk ratio (RR) of being missed or delayed due to COVID-19 for women compared to men was then given by:

$${\textit{RR}}_\textit{WM}\text{=}\frac{\textit{P}\text{(}\textit{missed|COVID,women}\text{)}}{\textit{P}\text{(}\textit{missed|COVID,men}\text{)}}$$

We interpreted RRs > 1 as women having had an increased risk of being missed or delayed, i.e. have been more negatively affected by COVID-19 than men, and RRs < 1 as men having had an increased risk of being missed or delayed, i.e. have been more negatively affected by COVID-19 than women. Confidence intervals for missed notifications were derived using a normal approximation of the log(count) scale.

Where observed notifications exceeded expected notifications, such as due to an increase in case finding, the probability of a missed or delayed diagnosis was 0. To avoid numerical issues when computing RRs and performing meta-analyses, we replaced observed notifications with expected notifications minus 0.5 in these cases. In the case where observed notifications exceeded expected in both the reference and the comparison groups, the corresponding RR is undefined and such cases were removed from the meta-analysis.

We used parametric bootstrap sampling to derive confidence intervals for the estimated relative risks. Using the estimated standard errors and the estimated predicted mean, we sampled from the approximate normal distribution on the link scale to generate sets of predicted notifications for both the reference and the comparison groups. In this way, using the sampled sets, we derived empirical distributions of the RR and used these to derive estimates for the 95% confidence intervals (using the percentile method) and standard errors of the RR, needed for the meta-analysis.

As we anticipated considerable between-country heterogeneity, a random-effects model was used to pool effect sizes. We conducted random-effects meta-analyses on the log risk ratios by WHO geographic region, estimating heterogeneity using the *I*^2^ statistic [21] to give the percentage of between-study variability that was not due to sampling error. The DerSimonian-Laird estimator [22] was used to calculate the heterogeneity variance. All analyses were conducted using the meta package [23] in R [24]. Model code can be found in Additional file 1.

We considered there to be strong evidence of an association between either age group or sex and risk of being affected by the pandemic if the *p*-value for the risk ratio was < 0.01 and the strength of association was meaningful, defined as an effect size of > 10%. We considered there to be some evidence of an association if the *p*-value was < 0.05 and the effect size was > 10%, and weak evidence (but cause for further investigation) if the *p*-value was ≥ 0.05 and < 0.1 but the effect size was very large, in this case > 25%. If the effect size was small (< 10%) or the *p*-value large (> 0.1), we considered that there was no evidence to conclude there was an association between age or sex and risk of being affected by the pandemic. We considered an *I*^2^ < 25% to reflect a small level of heterogeneity, 25% < *I*^2^ < 75% a moderate amount and *I*^2^ > 75% a high amount [25], although due to differences in confounding from a wide range of COVID-19-associated disruptions, a reasonable degree of heterogeneity was to be expected in our results.

### Sensitivity analysis

We conducted meta-analyses of different subgroups of countries to consider the robustness of our results. First, we analysed high TB burden, high TB/HIV burden and high MDR-TB burden countries separately. Second, we excluded countries where there was no evidence of an impact of COVID-19-associated disruptions on TB diagnosis (defined here as countries where notifications for one or more population groups in the analysis were higher than expected). Third, we excluded countries where the model fit was considered to be poor (pseudo *R*^2^ < 0.5) or where there were limited data available (for less than five years prior to 2020).

## Results

### Data

We included TB notification data from 45 countries with a high TB, TB/HIV or MDR-TB burden: 21 countries in the African Region, two in the Region of the Americas, two in the Eastern Mediterranean Region, nine in the European Region, seven in the South-East Asia Region and four in the Western Pacific Region. There were insufficient years of appropriately disaggregated data to include Angola, Mozambique, Papua New Guinea and Uganda. A total of 2,334,656 (6.9% of all notifications disaggregated by age) children (aged < 15 years), 27,448,386 (81.7%) adults (aged 15–64 years), and 3,828,418 (11.4%) elderly (aged ≥ 65 years) people with TB were notified from 2013–2019 across all 45 countries, including 19,961,383 (63.8% of all notifications disaggregated by sex) men (aged ≥ 15 years) and 11,346,972 (36.2%) women (aged ≥ 15 years). A total of 325,964 (6.5%) children, 4,079,324 (81.6%) adults, 595,840 (11.9%) elderly, 2,917,005 (62.4%) men and 1,758,159 (37.6%) women were notified in 2020 (see Table 1).

**Table 1:** Tuberculosis notifications for 2013–2020 by age and sex (children aged < 15 years, adults aged 15–64 years, elderly aged ≥ 65 years, men aged ≥ 15 years and women aged ≥ 15 years), aggregated across 45 high TB, TB/HIV and MDR-TB burden countries. Note that not all countries had notification data for all years

	2013	2014	2015	2016	2017	2018	2019	2020
Children	89,941	432,310	324,383	373,195	386,762	445,492	442,766	325,964
Adults	1,565,284	3,497,613	3,921,764	4,391,616	4,448,079	4,807,836	4,816,194	4,079,324
Elderly	235,275	272,117	517,191	591,488	580,333	721,210	750,611	595,840
Women	647,666	1,399,267	1,599,160	1,779,556	1,816,238	2,040,835	2,064,250	1,758,159
Men	1,152,893	2,530,656	2,871,346	3,203,548	3,212,174	3,488,211	3,503,555	2,917,005

**Fig. 1:**
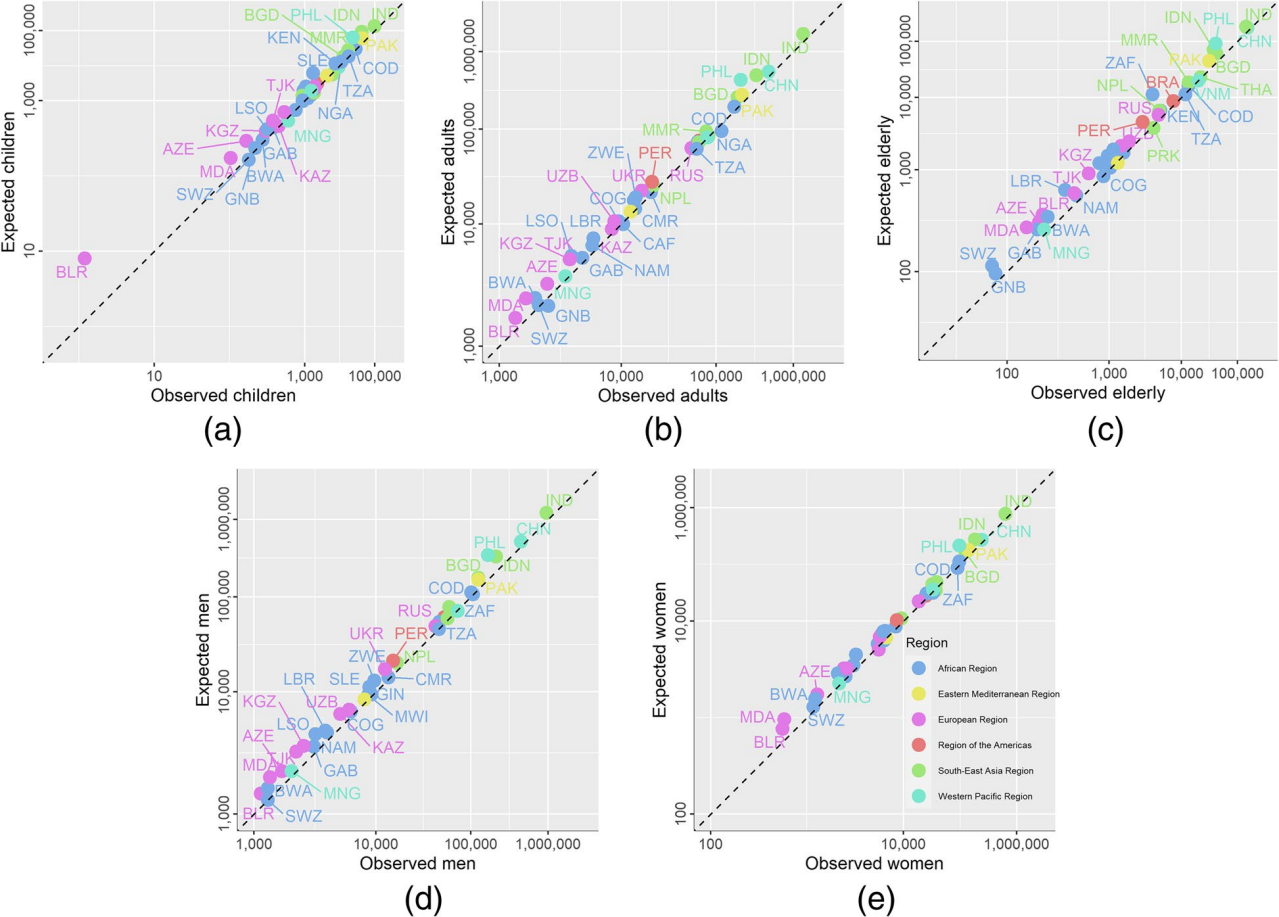
Observed and expected tuberculosis notifications in 2020 for 45 high TB, TB/HIV and MDR-TB burden countries for **a** children aged < 15 years, **b** adults aged 15–64 years, **c** elderly aged ≥ 65 years, **d** men aged ≥ 15 years and **e** women aged ≥ 15 years. Colours indicate the World Health Organization region for each country and labels indicate the iso3 code. 95% confidence intervals have been omitted as these are not visible at this scale

Country-specific Poisson models (see Additional file 1) suggest that compared to these observed notifications, an estimated 195,449 (95% confidence interval, CI: 189,673–201,562) of an expected 517,168 children (37.8%), 1,126,133 (CI: 1,107,146–1,145,704) of an expected 5,170,592 adults (21.8%) and 235,402 (CI: 228,108–243,202) of an expected 826,563 elderly (28.5%) were missed or delayed in their TB diagnosis in 2020 as a result of the pandemic. This included 511,546 (CI: 499,623–523,869) of an expected 2,250,097 women (22.7%) and 863,916 (CI: 847,591–880,515) of an expected 3,763,363 men (23.0%) (Fig. 1).

### Relative impact by age and sex

**Fig. 2:**
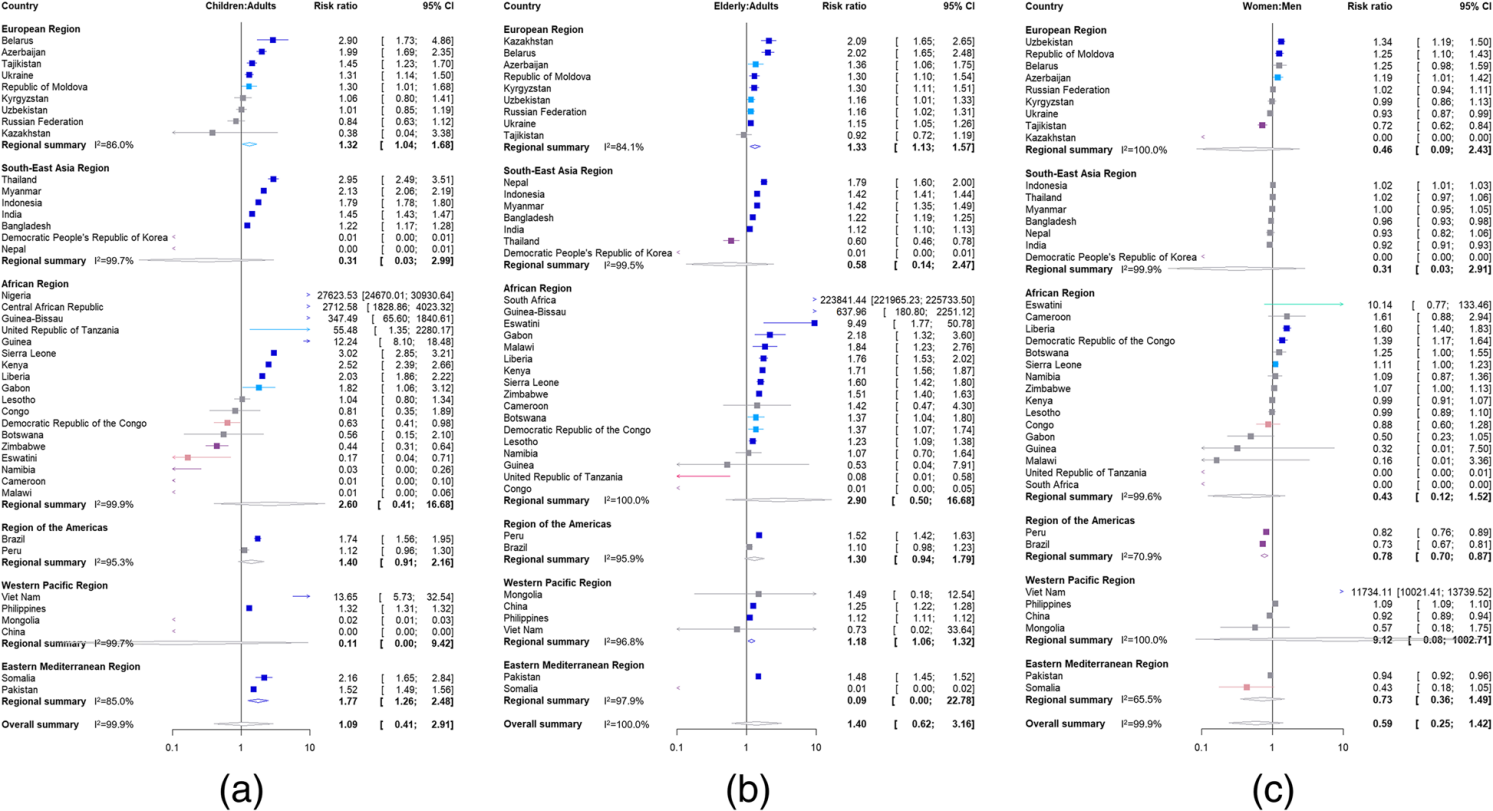
Risk ratios for disruption to tuberculosis notifications due to the pandemic for 45 high TB, TB/HIV and MDR-TB burden countries by WHO region for **a** children aged < 15 years compared to adults aged 15–64 years, **b** elderly aged ≥ 65 years compared to adults aged 15–64 years and **c** women aged ≥ 15 years compared to men aged ≥ 15 years. Risk ratios > 1 imply that the first population (children, the elderly or women) have had a larger proportion of diagnoses missed or delayed in 2020 as a result of the pandemic. Risk ratios < 1 imply that the second population (adults or men) have had a larger proportion of diagnoses missed or delayed in 2020 as a result of the pandemic. Countries where there were more notifications in both comparator and reference group were excluded from the meta-analysis. Colours indicate strength of evidence; no evidence for a risk ratio different to 1 (grey), strong evidence for a risk ratio > 1 (dark blue), evidence for a risk ratio > 1 (light blue), weak evidence for a risk ratio > 1 (green), strong evidence for a risk ratio < 1 (purple), evidence for a risk ratio < 1 (dark pink) and weak evidence for a risk ratio < 1 (light pink)

In 24 of 42 countries (57.1%) with fewer than predicted notifications for either children or adults, there was evidence (strong evidence in 21 countries, evidence in a further three) that missed or delayed diagnoses due to COVID-19 were associated with being a child (i.e. child-to-adult RR > 1). In ten countries (23.8%), there was evidence (strong evidence in eight countries, evidence in a further two) that missed or delayed diagnoses due to COVID-19 were associated with being an adult (i.e. child-to-adult RR < 1). There was no evidence of any association between risk and being either a child or an adult in the remaining eight countries (19.0%) (Fig. 2a).

In 29 of 41 countries (70.1%) with fewer than predicted notifications for either the elderly or adults, there was evidence (strong evidence in 24 countries, evidence in a further five) that missed or delayed diagnoses due to COVID-19 were associated with being elderly (i.e. elderly-to-adult RR > 1). In five countries (12.2%), there was evidence (strong evidence in four countries, evidence in a further one) that missed or delayed diagnoses due to COVID-19 were associated with being an adult (i.e. elderly-to-adult RR < 1). There was no evidence of any association between risk and being either elderly or an adult in the remaining seven countries (17.1%) (Fig. 2b).

In nine of 40 countries (22.5%) with fewer than predicted notifications for either men or women, there was evidence (strong evidence in five countries, evidence in three and weak evidence in a further one) that missed or delayed diagnoses due to COVID-19 were associated with being a woman (i.e. woman-to-man RR > 1). In nine countries (22.5%), there was evidence (strong evidence in seven countries, weak evidence in a further two) that missed or delayed diagnoses due to COVID-19 were associated with being a man (i.e. woman-to-man RR < 1). There was no evidence of any association between risk and being either a woman or a man in the remaining 22 countries (55.0%) (Fig. 2c).

### Meta-analyses

Evaluating the strength of evidence as defined in the methods above, there was strong evidence in the WHO Eastern Mediterranean (RR = 1.77 [95% CI 1.26–2.48, *I*^2^ = 84.9%]) and evidence in the European (RR = 1.32 [95% CI 1.04–1.68, *I*^2^ = 85.7%]) regions that notifications for children had been disproportionately affected compared to adults (see Fig. 2a). However, globally (RR = 1.09 [95% CI 0.41–2.91, *I*^2^ = 99.9%]) and in remaining WHO regions, there was no evidence that notifications for either children or adults have been disproportionately affected relative to one another.

There was strong evidence the WHO European (RR = 1.33 [95% CI 1.13–1.57, *I*^2^ = 84.8%]) and Western Pacific (RR = 1.18 [95% CI 1.06–1.32, *I*^2^ = 96.7%]) regions that notifications for the elderly have been disproportionately affected compared to adults (Fig. 2b). However, globally (RR = 1.40 [95% CI 0.62–3.16, *I*^2^ = 100.0%]) and in remaining WHO regions, there was no evidence that notifications for either the elderly or adults have been disproportionately affected relative to one another.

There was strong evidence that notifications for men have been disproportionately affected compared to women in the WHO Region of the Americas (RR = 0.78 [95% CI 0.70–0.87, *I*^2^ = 70.1%). Globally, and in the remaining WHO regions, there was no evidence that notifications for either men or women have been disproportionately affected (RR = 0.59 [95% CI 0.25–1.42, *I*^2^ = 99.9%) (Fig. 2c).

Further region- and country-specific results can be found in Additional file 1.

### Sensitivity analysis

Although there are some differences in regional RR when considering high TB, high TB/HIV and high MDR-TB burden countries separately, our conclusions are broadly qualitatively similar, with no evidence globally for any difference in risk by age or sex.

Whilst overall and in most age and sex groups there was a reduction in TB cases notified in 2020 compared to expected numbers based on 2013–2019 trends, in 18 countries, there were more notifications than expected in at least one age or sex group; Cameroon, Central African Republic, China, Congo, Democratic People’s Republic of Korea, Eswatini, Ethiopia, Guinea-Bissau, Kazakhstan, Malawi, Mongolia, Nepal, Nigeria, Somalia, South Africa, United Republic of Tanzania, Viet Nam and Zambia. Removing these countries from our analysis, we found that there was strong evidence globally (RR = 1.57 [95% CI 1.22–2.03, *I*^2^ = 99.6%]) that notifications for children have been disproportionately affected compared to adults. There was also strong evidence globally (RR = 1.36 [95% CI 1.25–1.48, *I*^2^ = 98.7%]) that notifications for the elderly have been disproportionately affected compared to adults. However, there remained no evidence globally (RR = 1.02 [95% CI 0.96–1.09, *I*^2^ = 97.5%]) that notifications for either men or women have been disproportionately affected.

We considered there to be a poor model fit or limited data for children compared to adults in 26 countries, for the elderly compared to adults in 24 countries and for women compared to men in 22 countries. Removing these countries from our analysis, we found that there was no evidence globally that notifications for either children or adults (RR = 0.65 [95% CI 0.32–2.03, *I*^2^ = 99.9%]) were disproportionately affected. However, there was strong evidence globally (RR = 1.24 [95% CI 1.08–1.42, *I*^2^ = 99.0%]) that notifications for the elderly were disproportionately affected compared to adults and weak evidence (RR = 0.39 [95% CI 0.13–1.15, *I*^2^ = 100%]) that men were disproportionately affected compared to women.

See additional file 1 for further details.

## Discussion

The COVID-19 pandemic has caused significant disruptions to TB services, as indicated by substantially lower case notifications than expected in 2020. Compared to trends predicted from the pre-pandemic era, our results indicate that global TB notification rates were 21.8% lower than expected for adults (with a similar rate for both men and women), whilst for children and the elderly notification rates were 37.8% and 28.5% lower than expected, respectively. These findings suggest that a large number of individuals are likely suffering from untreated TB disease directly as a result of the pandemic. These individuals are also at risk of transmitting infection to their contacts, which will have long term public health consequences.

Our results are in line with most findings that COVID-19-associated disruptions have led to widespread decreases in TB diagnosis [1] and demonstrate that major inequalities in the impact of COVID-19-associated disruptions on TB diagnosis exist in high TB burden countries. However, these inequalities are extremely setting-specific, and there is no evidence of any systematic inequality in disruptions by age or sex globally. In over a half of countries, including in the WHO Eastern Mediterranean and European regions, the risk of having diagnosis missed or delayed as a result of the pandemic was higher for children than adults. However, high levels of heterogeneity and the presence of a small number of countries where more children than expected were diagnosed mean that there was no evidence for any difference in risk globally. In more than two thirds of countries, and in the WHO European and Western Pacific regions, the risk of having diagnosis missed or delayed was higher for the elderly than adults. Again, however, high levels of heterogeneity mean that this inequality in risk was not evident globally, although in countries that did experience disruptions and those with better model fits the elderly did also appear to be at a higher risk. In nearly a half of countries, the risk of having diagnosis missed or delayed differed by sex. This varied by setting, with men in the Region of the Americas representing a particular concern. However, globally there was no evidence for any difference in risk. Given already low case detection rates for children, and high burden in men and the elderly, it is encouraging that COVID-19-associated disruptions have not worsened this inequality. However, it is concerning that in countries that did appear to experience disruptions, or those with a better model fit, children, the elderly and men are all at a higher risk as a result.

In most of our analyses, heterogeneity between countries was extremely high, which we were unable to account for due to multiple sources of confounding. Although some heterogeneity was attributable to regional differences, differences between countries in population age-structure, gender roles and responsibilities, COVID-19 non-pharmaceutical interventions, COVID-19 burden on health care services, barriers to accessing health care services due to cultural practices, individual’s personal protection measures and timing of all of the above likely all contribute. Given a lack of data, no meta-regression was conducted. It is unclear why some countries demonstrate inequalities in one direction whilst others demonstrate no inequality, or the opposite. Causal drivers are likely to be highly setting specific, where identifying these drivers requires a close examination of a country’s experience of the pandemic. Whilst our analysis is not able to suggest why men in Tajikistan may have been more likely to miss or delay a diagnosis in 2020 whilst women in Uzbekistan were more likely to do so, it does point to a clear need for further, gender-sensitive analyses when planning the response in both of those countries.

Our results have focused on the year 2020. We note that our analysis is not able to capture diagnoses made in 2020 which would have occurred earlier in the year but were delayed because of COVID-19. Similarly, catch-up campaigns in 2020 may mask delays to diagnosis from earlier in the year. Although COVID-19-associated disruptions will have affected different countries at different time points throughout 2020, the use of a risk ratio here allows for a comparison in risk within, and between, countries during those periods. Extrapolating this analysis to future years should be conducted with extreme caution. The long duration of progression from *M.tb* infection to TB disease [26] suggests that changes in transmission of *M.tb* as a result of COVID-19-associated disruptions are unlikely to have had a major influence on TB disease burden and notifications in 2020, although the effect may still be non-negligible. However, from 2021 as COVID-19-related disruptions continue, it is likely that societal changes from earlier in the pandemic, such as an increased proportion of time spent in the household and not the workplace, may affect transmission dynamics (and therefore the underlying TB disease burden) differently in different groups, so it is no longer clear how best to estimate a “missed or delayed diagnosis”.

Our results are limited by both the availability of data and the methods used to estimate missing cases. Although changes to *M.tb* transmission are unlikely to have affected TB burden during the time period under consideration, it is possible that an increase in risk factors such as undernutrition may have, where the extent of this may vary by population group. In addition, an observed increase or decrease in notifications does not necessarily correspond to a change in diagnosis, and could instead reflect changes in reporting, although this would probably not vary by population group. In some countries, including a number in Africa in particular, attempts to counter the effects of COVID-19-associated disruptions, such as a sustained effort to increase case detection, could also have led to more notifications than expected. However, this could also reflect other factors, such as a high uptake of non-pharmaceutical interventions with a corresponding reduction in *M.tb* transmission, or the importance of rapid progression as opposed to remote reactivation in that setting. In general, we are not able to account for country-specific events, such as natural disasters or political unrest, which may also have affected care seeking behaviour in 2020 of one population group over another. Our analysis is predicated on good model predictions, where the use of a simple linear predictor term in our Poisson models may be unrealistic in some countries. However, given the limited data available, any more complicated model may overfit the data.

## Conclusions

Despite these limitations, overall, we found no evidence at a global level of any difference by age or sex in the risk of disruption to TB diagnosis as a result of the pandemic. However, in many specific settings, the consequences of disruptions have been greater for some groups than others, with children and the elderly in particular being disproportionately affected in the majority of countries. It is important to recognise these inequalities when prioritising catch-up campaigns and the cultural and social issues that have contributed to them. For example, in countries where children have been disproportionately affected, active case finding interventions to mitigate the impact could prioritise schools or TB-affected households, whilst in countries where men have been disproportionately affected, out-reach campaigns and extended opening hours for clinical services could improve access to healthcare for men. Our results highlight the importance and utility of tracking age and sex differences in healthcare reporting to explore the impact of disease across these dimensions. Such data would allow for a nuanced, equitable response not just to COVID-19, but to the mitigation of humanitarian crises in general. There is a clear need for context-specific data and evidence-informed action to achieve equity in TB care, with the ultimate aim of ending TB.

## Supplementary Information

**Additional file 1:** Model code and additional tables and Figs. **Table S1.** Country-specific tuberculosis notifications for 2013-2020 by age and sex. **Table S2.** Country-specific linear models for expected notifications. **Fig. S1.** Country-specific linear models for expected notifications. **Table S3.** Country-specific numbers of missed or delayed diagnoses. **Table S4.** Country-specific risk-ratios for disruption to tuberculosis notifications due to the pandemic for men compared to women (both aged ≥ 15 years). **Table S5.** Country-specific risk-ratios for disruption to tuberculosis notifications due to the pandemic for children (aged < 15 years) and the elderly (aged ≥ 65 years) compared to adults (aged 15-64 years).

## Data Availability

All data generated or analysed during this study are included in this published article and its supplementary information files or through the World Health Organization repository: https://www.who.int/tb/country/data/download/en/.

## Abbreviations

CI: 95% Confidence interval
MDR-TB: Multidrug- or rifampicin-resistant tuberculosis
*M.tb*: Mycobacterium tuberculosis
RR: Risk ratio
TB: Tuberculosis
WHO: World Health Organization
