# Supplementary material for: Inequalities in the impact of COVID-19-associated disruptions on tuberculosis diagnosis by age and sex in 45 high TB burden countries
Source: BMC Med. 2022 Nov 14;20:432. doi: 10.1186/s12916-022-02624-6 (PMC9660190; doi:10.1186/s12916-022-02624-6)
Supplement: Supplementary file 1 — Additional file 1: Model code and additional tables and Figs. Table S1. Country-specific tuberculosis notifications for 2013-2020 by age and sex. Table S2. Country-specific linear models for expected notifications. Fig. S1. Country-specific linear models for expected notifications. Table S3. Country-specific numbers of missed or delayed diagnoses. Table S4. Country-specific risk-ratios for disruption to tuberculosis notifications due to the pandemic for men compared to women (both aged ≥ 15 years). Table S5. Country-specific risk-ratios for disruption to tuberculosis notifications due to the pandemic for children (aged < 15 years) and the elderly (aged ≥ 65 years) compared to adults (aged 15-64 years). [file 12916_2022_2624_MOESM1_ESM.html]

Inequalities in the impact of COVID-19-associated disruptions on tuberculosis diagnosis by age and sex in 45 high TB burden countries


Code 

- Show All Code
- Hide All Code

# Inequalities in the impact of COVID-19-associated disruptions on tuberculosis diagnosis by age and sex in 45 high TB burden countries

#### C Finn McQuaid, Marc YR Henrion, Rachael M Burke, Peter MacPherson, Rebecca Nzawa-Soko, Katherine C Horton

#### 30 June 2022

- 1. Background and
  required packages
- 2. Loading data
- 3. Linear models
- 4. Plot models
- 5. Reformat data
- 6. Compute risks
- 7. Missed cases
- 8. Sex risk ratios
- 9. Age risk ratios
- 10. Plot missing cases
- 11. Plot meta-analyses

# 1. Background and required packages

We set the working file directory and load all required R packages
for analysis into R.

# 2. Loading data

We load the required data and select countries of interest

```
# High TB burden country lists
country_HBC<-c('Angola','Bangladesh','Brazil','Central African Republic','China','Congo','Democratic People\'s Republic of Korea','Democratic Republic of the Congo','Ethiopia','Gabon','India','Indonesia','Kenya','Lesotho','Liberia','Mongolia','Mozambique','Myanmar','Namibia','Nigeria','Pakistan','Papua New Guinea','Philippines','Sierra Leone','South Africa','Thailand','Uganda','United Republic of Tanzania','Viet Nam','Zambia')
country_MDR<-c('Angola','Azerbaijan','Bangladesh','Belarus','China','Democratic People\'s Republic of Korea','Democratic Republic of the Congo','India','Indonesia','Kazakhstan','Kyrgyzstan','Mongolia','Mozambique','Myanmar','Nepal','Nigeria','Pakistan','Papua New Guinea','Peru','Philippines','Republic of Moldova','Russian Federation','Somalia','South Africa','Tajikistan','Ukraine','Uzbekistan','Viet Nam','Zambia','Zimbabwe')
country_HIV<-c('Botswana','Brazil','Cameroon','Central African Republic','China','Congo','Democratic Republic of the Congo','Eswatini','Ethiopia','Gabon','Guinea','Guinea-Bissau','India','Indonesia','Kenya','Lesotho','Liberia','Malawi','Mozambique','Myanmar','Namibia','Nigeria','Philippines','Russian Federation','South Africa','Thailand','Uganda','United Republic of Tanzania','Zambia','Zimbabwe')
country_list<-unique(c(country_HBC,country_MDR,country_HIV))
# Remove Mozambique & Uganda as incomplete age data, Angola and Papua New Guinea as only two data points prior to 2020
country_list<-country_list[!country_list %in% c("Angola","Mozambique","Papua New Guinea","Uganda")]
# Read in WHO notification data from https://www.who.int/teams/global-tuberculosis-programme/data
WHO_not<-fread('TB_notifications_2022-06-15.csv')
# Use only HBC countries
WHO_not$g_whoregion<-mgsub(WHO_not$g_whoregion,c("AFR","AMR","EMR","EUR","SEA","WPR"),c("African Region","Region of the Americas","Eastern Mediterranean Region","European Region","South-East Asia Region","Western Pacific Region"))
WHO_not<-WHO_not[country%in%country_list]
# Remove years where new cases only (not new + relapse) are recorded
# Except Azerbaijan where this is always the case, and Mongolia where there is a blank for 2020
WHO_not[country=="Azerbaijan",rel_in_agesex_flg:=1]
WHO_not[country=="Mongolia",rel_in_agesex_flg:=1]
WHO_not<-WHO_not[rel_in_agesex_flg==1]   
WHO_not<-WHO_not[year%in%c(2013:2020)]
# Define categories
WHO_not[,men:=newrel_m15plus]
WHO_not[,women:=newrel_f15plus]
WHO_not[,children:=newrel_m014+newrel_f014]
WHO_not[,adults:=newrel_m1524+newrel_m2534+newrel_m3544+newrel_m4554+newrel_m5564+newrel_f1524+newrel_f2534+newrel_f3544+newrel_f4554+newrel_f5564]
WHO_not[,elderly:=newrel_m65+newrel_f65]
# Simplify data set
dat<-select(WHO_not,country,iso3,g_whoregion,year,men,women,children,adults,elderly)
options(knitr.kable.NA = '')
# Show data
dat %>%
  dplyr::select(!c(country)) %>%
  knitr::kable(col.names=c("Country code","Region","Year","Men (observed)","Women (observed)","Children (observed)","Adults (observed)","Elderly (observed)")) %>%
  kableExtra::kable_styling(full_width = FALSE)
```

| Country code | Region | Year | Men (observed) | Women (observed) | Children (observed) | Adults (observed) | Elderly (observed) |
| --- | --- | --- | --- | --- | --- | --- | --- |
| AZE | European Region | 2013 | 3145 | 1168 | 215 | 4114 | 199 |
| AZE | European Region | 2014 | 3107 | 1103 | 179 | 4010 | 200 |
| AZE | European Region | 2015 | 2656 | 1154 | 179 | 3599 | 211 |
| AZE | European Region | 2016 | 2470 | 1148 | 175 | 3436 | 182 |
| AZE | European Region | 2017 | 2440 | 1265 | 166 | 3473 | 232 |
| AZE | European Region | 2018 | 2382 | 1180 | 200 | 3305 | 257 |
| AZE | European Region | 2019 | 2307 | 1127 | 179 | 3166 | 268 |
| AZE | European Region | 2020 | 1610 | 857 | 91 | 2282 | 185 |
| BGD | South-East Asia Region | 2015 | 118612 | 80222 | 8073 | 169345 | 29489 |
| BGD | South-East Asia Region | 2016 | 125646 | 87336 | 9266 | 179283 | 33699 |
| BGD | South-East Asia Region | 2017 | 134651 | 97817 | 10171 | 193871 | 38597 |
| BGD | South-East Asia Region | 2018 | 147490 | 108351 | 11302 | 211390 | 44451 |
| BGD | South-East Asia Region | 2019 | 159498 | 119767 | 12330 | 228291 | 50974 |
| BGD | South-East Asia Region | 2020 | 123451 | 97264 | 9363 | 181071 | 39644 |
| BLR | European Region | 2013 | 3232 | 1224 | 14 | 3974 | 482 |
| BLR | European Region | 2014 | 2796 | 1038 | 24 | 3404 | 430 |
| BLR | European Region | 2015 | 2740 | 1007 | 18 | 3250 | 497 |
| BLR | European Region | 2016 | 2291 | 907 | 13 | 2778 | 420 |
| BLR | European Region | 2017 | 2007 | 761 | 13 | 2386 | 382 |
| BLR | European Region | 2018 | 1729 | 618 | 12 | 2000 | 347 |
| BLR | European Region | 2019 | 1603 | 596 | 8 | 1853 | 346 |
| BLR | European Region | 2020 | 1121 | 389 | 4 | 1310 | 200 |
| BWA | African Region | 2013 | 3613 | 2665 | 556 | 5783 | 495 |
| BWA | African Region | 2014 | 3274 | 2324 | 419 | 5133 | 465 |
| BWA | African Region | 2015 | 2662 | 2014 | 296 | 4327 | 349 |
| BWA | African Region | 2016 | 2690 | 1825 | 288 | 4097 | 418 |
| BWA | African Region | 2017 | 2205 | 1573 | 319 | 3427 | 351 |
| BWA | African Region | 2018 | 1995 | 1426 | 229 | 3058 | 363 |
| BWA | African Region | 2019 | 1703 | 1154 | 232 | 2523 | 334 |
| BWA | African Region | 2020 | 1258 | 811 | 160 | 1845 | 224 |
| BRA | Region of the Americas | 2013 | 50540 | 24365 | 2501 | 67682 | 7223 |
| BRA | Region of the Americas | 2014 | 50072 | 23641 | 2233 | 66628 | 7085 |
| BRA | Region of the Americas | 2015 | 50669 | 22793 | 2097 | 66145 | 7317 |
| BRA | Region of the Americas | 2016 | 51209 | 22857 | 2173 | 66553 | 7513 |
| BRA | Region of the Americas | 2017 | 53862 | 22990 | 2264 | 68891 | 7961 |
| BRA | Region of the Americas | 2018 | 57314 | 24489 | 2547 | 73560 | 8243 |
| BRA | Region of the Americas | 2019 | 57597 | 24839 | 2681 | 73833 | 8603 |
| BRA | Region of the Americas | 2020 | 49967 | 21522 | 1944 | 64037 | 7452 |
| CMR | African Region | 2016 | 14334 | 9792 | 1401 | 22794 | 1332 |
| CMR | African Region | 2017 | 13868 | 9309 | 1350 | 21696 | 1481 |
| CMR | African Region | 2018 | 13520 | 8705 | 1164 | 20776 | 1449 |
| CMR | African Region | 2019 | 14209 | 8855 | 1255 | 21496 | 1568 |
| CMR | African Region | 2020 | 13194 | 7750 | 1158 | 19432 | 1512 |
| CAF | African Region | 2016 | 5497 | 3703 | 1418 | 8847 | 353 |
| CAF | African Region | 2017 | 4723 | 3516 | 1580 | 7913 | 326 |
| CAF | African Region | 2018 | 5246 | 3888 | 1861 | 8749 | 385 |
| CAF | African Region | 2019 | 5919 | 4492 | 1648 | 9995 | 416 |
| CAF | African Region | 2020 | 6263 | 4643 | 1629 | 10419 | 487 |
| CHN | Western Pacific Region | 2013 | 583598 | 258748 | 4830 | 670432 | 171914 |
| CHN | Western Pacific Region | 2014 | 566364 | 248755 | 4164 | 643948 | 171171 |
| CHN | Western Pacific Region | 2015 | 550996 | 243245 | 4198 | 618127 | 176114 |
| CHN | Western Pacific Region | 2016 | 535618 | 238185 | 4690 | 599657 | 174146 |
| CHN | Western Pacific Region | 2017 | 533295 | 234862 | 4993 | 587319 | 180838 |
| CHN | Western Pacific Region | 2018 | 541876 | 246948 | 6421 | 591395 | 197429 |
| CHN | Western Pacific Region | 2019 | 497696 | 223913 | 6656 | 537497 | 184112 |
| CHN | Western Pacific Region | 2020 | 423296 | 194405 | 7014 | 458615 | 159086 |
| COG | African Region | 2015 | 4388 | 3465 | 783 | 7326 | 527 |
| COG | African Region | 2016 | 5294 | 4037 | 1093 | 8669 | 662 |
| COG | African Region | 2017 | 5126 | 4002 | 877 | 8489 | 639 |
| COG | African Region | 2018 | 5530 | 4256 | 920 | 9093 | 693 |
| COG | African Region | 2019 | 6269 | 4565 | 968 | 10048 | 786 |
| COG | African Region | 2020 | 5902 | 4376 | 897 | 9419 | 859 |
| PRK | South-East Asia Region | 2017 | 59817 | 35525 | 5211 | 90526 | 4816 |
| PRK | South-East Asia Region | 2018 | 54936 | 30250 | 4753 | 81369 | 3817 |
| PRK | South-East Asia Region | 2019 | 59039 | 32057 | 4626 | 87057 | 4039 |
| PRK | South-East Asia Region | 2020 | 54238 | 30786 | 4616 | 81199 | 3825 |
| COD | African Region | 2016 | 64101 | 48684 | 14213 | 105880 | 6905 |
| COD | African Region | 2017 | 75123 | 57708 | 16933 | 123590 | 9241 |
| COD | African Region | 2018 | 85290 | 65908 | 18453 | 140840 | 10358 |
| COD | African Region | 2020 | 101798 | 76418 | 22340 | 165082 | 13134 |
| SWZ | African Region | 2013 | 3318 | 2702 | 671 | 5713 | 307 |
| SWZ | African Region | 2014 | 2830 | 2251 | 502 | 4831 | 250 |
| SWZ | African Region | 2015 | 2473 | 1797 | 297 | 4024 | 246 |
| SWZ | African Region | 2016 | 2065 | 1524 | 217 | 3393 | 196 |
| SWZ | African Region | 2017 | 1833 | 1356 | 178 | 3015 | 174 |
| SWZ | African Region | 2018 | 1622 | 1204 | 161 | 2679 | 147 |
| SWZ | African Region | 2019 | 1580 | 1120 | 176 | 2574 | 126 |
| SWZ | African Region | 2020 | 1258 | 783 | 99 | 1965 | 76 |
| ETH | African Region | 2015 | 64509 | 51722 | 18444 | 110185 | 6046 |
| ETH | African Region | 2016 | 61198 | 48956 | 15100 | 104790 | 5364 |
| ETH | African Region | 2017 | 58941 | 44800 | 12810 | 98316 | 5425 |
| ETH | African Region | 2018 | 56517 | 44866 | 12053 | 95946 | 5437 |
| ETH | African Region | 2019 | 56092 | 43923 | 11024 | 94677 | 5338 |
| ETH | African Region | 2020 | 54956 | 42398 | 10839 | 92146 | 5208 |
| GAB | African Region | 2014 | 2955 | 2190 | 463 | 4758 | 387 |
| GAB | African Region | 2015 | 3166 | 2034 | 483 | 4795 | 405 |
| GAB | African Region | 2016 | 3127 | 2060 | 380 | 4799 | 388 |
| GAB | African Region | 2018 | 3339 | 2059 | 291 | 5128 | 270 |
| GAB | African Region | 2019 | 3138 | 1894 | 367 | 4783 | 249 |
| GAB | African Region | 2020 | 2861 | 1777 | 241 | 4458 | 180 |
| GIN | African Region | 2013 | 6993 | 4135 | 185 | 10467 | 661 |
| GIN | African Region | 2015 | 8231 | 3730 | 193 | 11562 | 399 |
| GIN | African Region | 2016 | 7715 | 4259 | 665 | 11411 | 563 |
| GIN | African Region | 2017 | 8043 | 4808 | 858 | 12133 | 718 |
| GIN | African Region | 2018 | 8363 | 4964 | 923 | 12490 | 837 |
| GIN | African Region | 2019 | 9599 | 5634 | 1157 | 14156 | 1077 |
| GIN | African Region | 2020 | 9066 | 5601 | 945 | 13632 | 1035 |
| GNB | African Region | 2014 | 1348 | 823 | 108 | 2083 | 88 |
| GNB | African Region | 2016 | 1373 | 760 | 90 | 2034 | 99 |
| GNB | African Region | 2017 | 1293 | 808 | 125 | 2007 | 94 |
| GNB | African Region | 2018 | 1233 | 655 | 137 | 1802 | 86 |
| GNB | African Region | 2019 | 1432 | 856 | 119 | 2189 | 99 |
| GNB | African Region | 2020 | 1518 | 903 | 122 | 2340 | 81 |
| IND | South-East Asia Region | 2013 |  |  |  |  |  |
| IND | South-East Asia Region | 2014 | 1010620 | 503218 | 95709 | 1380532 | 133306 |
| IND | South-East Asia Region | 2015 | 1046780 | 521223 | 99133 | 1429927 | 138076 |
| IND | South-East Asia Region | 2016 | 1107520 | 551470 | 104886 | 1512902 | 146088 |
| IND | South-East Asia Region | 2017 | 1020782 | 527742 | 101170 | 1450134 | 98390 |
| IND | South-East Asia Region | 2018 | 1142605 | 641240 | 124839 | 1608054 | 175791 |
| IND | South-East Asia Region | 2019 | 1272074 | 744675 | 145574 | 1805547 | 211202 |
| IND | South-East Asia Region | 2020 | 949552 | 582210 | 97539 | 1383074 | 148688 |
| IDN | South-East Asia Region | 2013 | 175558 | 123970 | 26054 | 276940 | 22588 |
| IDN | South-East Asia Region | 2014 | 177044 | 122592 | 23170 | 274303 | 25333 |
| IDN | South-East Asia Region | 2015 | 178106 | 122377 | 28412 | 272316 | 28167 |
| IDN | South-East Asia Region | 2016 | 192516 | 133490 | 32602 | 294192 | 31814 |
| IDN | South-East Asia Region | 2017 | 226819 | 158867 | 52944 | 344402 | 41284 |
| IDN | South-East Asia Region | 2018 | 288166 | 208253 | 68550 | 439922 | 56497 |
| IDN | South-East Asia Region | 2019 | 284339 | 203886 | 70040 | 433137 | 55088 |
| IDN | South-East Asia Region | 2020 | 203415 | 145149 | 35461 | 313183 | 35381 |
| KAZ | European Region | 2013 | 11604 | 6843 | 511 | 17242 | 1205 |
| KAZ | European Region | 2014 | 9000 | 5792 | 452 | 13795 | 997 |
| KAZ | European Region | 2015 | 8314 | 5300 | 392 | 12469 | 1145 |
| KAZ | European Region | 2016 | 7463 | 4533 | 326 | 10921 | 1075 |
| KAZ | European Region | 2017 | 7342 | 4703 | 404 | 10797 | 1248 |
| KAZ | European Region | 2018 | 7655 | 4815 | 362 | 11096 | 1374 |
| KAZ | European Region | 2019 | 7396 | 4754 | 351 | 10729 | 1421 |
| KAZ | European Region | 2020 | 4756 | 4544 | 303 | 8155 | 1145 |
| KEN | African Region | 2014 | 49810 | 31036 | 8448 | 76448 | 4398 |
| KEN | African Region | 2016 | 44799 | 24865 | 6671 | 65866 | 3798 |
| KEN | African Region | 2017 | 49308 | 26643 | 7648 | 71213 | 4738 |
| KEN | African Region | 2018 | 54719 | 29847 | 9968 | 78022 | 6544 |
| KEN | African Region | 2019 | 50008 | 26038 | 8299 | 70579 | 5467 |
| KEN | African Region | 2020 | 44034 | 22006 | 5606 | 61285 | 4755 |
| KGZ | European Region | 2015 | 3814 | 2676 | 537 | 5966 | 524 |
| KGZ | European Region | 2016 | 3808 | 2811 | 407 | 5965 | 654 |
| KGZ | European Region | 2017 | 3625 | 2626 | 436 | 5541 | 710 |
| KGZ | European Region | 2018 | 3586 | 2442 | 310 | 5277 | 751 |
| KGZ | European Region | 2019 | 3384 | 2449 | 305 | 5028 | 805 |
| KGZ | European Region | 2020 | 2370 | 1691 | 180 | 3480 | 581 |
| LSO | African Region | 2014 | 4803 | 3543 | 368 | 7740 | 606 |
| LSO | African Region | 2015 | 4469 | 2710 | 244 | 6581 | 598 |
| LSO | African Region | 2016 | 4199 | 2538 | 281 | 6075 | 662 |
| LSO | African Region | 2017 | 4324 | 2413 | 380 | 5943 | 794 |
| LSO | African Region | 2018 | 4361 | 2406 | 260 | 5842 | 925 |
| LSO | African Region | 2019 | 4373 | 2402 | 284 | 5678 | 1097 |
| LSO | African Region | 2020 | 2930 | 1444 | 191 | 3607 | 767 |
| LBR | African Region | 2013 | 2277 | 1437 | 121 | 3552 | 162 |
| LBR | African Region | 2016 | 4293 | 2015 | 872 | 6132 | 176 |
| LBR | African Region | 2018 | 3724 | 2858 | 1216 | 6146 | 436 |
| LBR | African Region | 2019 | 3846 | 3022 | 1413 | 6398 | 470 |
| LBR | African Region | 2020 | 3550 | 2349 | 1056 | 5569 | 330 |
| MWI | African Region | 2013 | 8383 | 6585 | 1827 | 13888 | 1080 |
| MWI | African Region | 2014 | 8724 | 5716 | 1827 | 13357 | 1083 |
| MWI | African Region | 2015 | 8576 | 5243 | 1562 | 12830 | 989 |
| MWI | African Region | 2016 | 8553 | 5327 | 1374 | 12903 | 977 |
| MWI | African Region | 2017 | 9283 | 5528 | 1701 | 13546 | 1265 |
| MWI | African Region | 2018 | 8837 | 5396 | 1399 | 12839 | 1394 |
| MWI | African Region | 2019 | 9431 | 5944 | 1527 | 13733 | 1642 |
| MWI | African Region | 2020 | 8468 | 5270 | 1395 | 12316 | 1422 |
| MNG | Western Pacific Region | 2013 | 2255 | 1717 | 359 | 3788 | 184 |
| MNG | Western Pacific Region | 2014 | 2361 | 1733 | 389 | 3894 | 200 |
| MNG | Western Pacific Region | 2015 | 2429 | 1832 | 424 | 4029 | 232 |
| MNG | Western Pacific Region | 2016 | 2152 | 1754 | 519 | 3696 | 210 |
| MNG | Western Pacific Region | 2017 | 2142 | 1651 | 427 | 3578 | 215 |
| MNG | Western Pacific Region | 2018 | 2052 | 1533 | 295 | 3367 | 218 |
| MNG | Western Pacific Region | 2019 | 2235 | 1636 | 406 | 3646 | 225 |
| MNG | Western Pacific Region | 2020 | 1908 | 1498 | 455 | 3198 | 208 |
| MMR | South-East Asia Region | 2014 | 65260 | 36727 | 36301 | 89237 | 12750 |
| MMR | South-East Asia Region | 2015 | 66193 | 37282 | 34930 | 90262 | 13213 |
| MMR | South-East Asia Region | 2016 | 67911 | 37977 | 31633 | 91649 | 14239 |
| MMR | South-East Asia Region | 2017 | 65783 | 35908 | 28723 | 87626 | 14065 |
| MMR | South-East Asia Region | 2018 | 71961 | 39740 | 26262 | 95308 | 16393 |
| MMR | South-East Asia Region | 2019 | 71566 | 39208 | 23703 | 93884 | 16890 |
| MMR | South-East Asia Region | 2020 | 58384 | 31724 | 13244 | 77017 | 13091 |
| NAM | African Region | 2013 | 4936 | 3402 | 1094 | 7827 | 511 |
| NAM | African Region | 2016 | 4710 | 3306 | 841 | 7433 | 583 |
| NAM | African Region | 2017 | 4658 | 3105 | 812 | 7188 | 575 |
| NAM | African Region | 2018 | 4430 | 2665 | 713 | 6630 | 465 |
| NAM | African Region | 2019 | 4406 | 2662 | 733 | 6561 | 507 |
| NAM | African Region | 2020 | 3684 | 2181 | 644 | 5435 | 430 |
| NPL | South-East Asia Region | 2015 | 18624 | 10255 | 2106 | 25099 | 3780 |
| NPL | South-East Asia Region | 2016 | 18367 | 10197 | 1776 | 24709 | 3855 |
| NPL | South-East Asia Region | 2017 | 18706 | 10400 | 1785 | 24543 | 4563 |
| NPL | South-East Asia Region | 2018 | 19216 | 10880 | 1625 | 25018 | 5078 |
| NPL | South-East Asia Region | 2019 | 18964 | 10710 | 1723 | 24040 | 5634 |
| NPL | South-East Asia Region | 2020 | 15950 | 9221 | 1619 | 20567 | 4604 |
| NGA | African Region | 2013 | 56513 | 38112 | 5776 | 87935 | 6690 |
| NGA | African Region | 2014 | 52028 | 33863 | 5463 | 79889 | 6002 |
| NGA | African Region | 2015 | 53131 | 32680 | 4773 | 79658 | 6153 |
| NGA | African Region | 2016 | 57163 | 34872 | 5244 | 85230 | 6805 |
| NGA | African Region | 2017 | 60115 | 35022 | 7250 | 88186 | 6951 |
| NGA | African Region | 2018 | 60238 | 35512 | 8171 | 88250 | 7500 |
| NGA | African Region | 2019 | 66842 | 40845 | 9462 | 98550 | 9137 |
| NGA | African Region | 2020 | 77890 | 49417 | 8349 | 116084 | 11223 |
| PAK | Eastern Mediterranean Region | 2014 | 141052 | 140120 | 27245 | 252774 | 28398 |
| PAK | Eastern Mediterranean Region | 2015 | 145515 | 143382 | 34370 | 258425 | 30472 |
| PAK | Eastern Mediterranean Region | 2016 | 160044 | 154588 | 41758 | 279189 | 35443 |
| PAK | Eastern Mediterranean Region | 2017 | 162517 | 152038 | 44373 | 276395 | 38160 |
| PAK | Eastern Mediterranean Region | 2018 | 162451 | 149764 | 47804 | 273128 | 39087 |
| PAK | Eastern Mediterranean Region | 2019 | 148072 | 134450 | 45447 | 244869 | 37653 |
| PAK | Eastern Mediterranean Region | 2020 | 123720 | 112219 | 37051 | 206818 | 29121 |
| PER | Region of the Americas | 2015 | 17421 | 10853 | 1559 | 24856 | 3418 |
| PER | Region of the Americas | 2016 | 16685 | 11464 | 1584 | 24567 | 3582 |
| PER | Region of the Americas | 2017 | 17587 | 10736 | 1517 | 24839 | 3484 |
| PER | Region of the Americas | 2018 | 19571 | 10521 | 1329 | 26045 | 4047 |
| PER | Region of the Americas | 2019 | 19458 | 10521 | 1363 | 25931 | 4048 |
| PER | Region of the Americas | 2020 | 14605 | 7952 | 933 | 19889 | 2668 |
| PHL | Western Pacific Region | 2014 | 32479 | 14486 | 12191 | 42634 | 4331 |
| PHL | Western Pacific Region | 2015 | 154050 | 78808 | 32813 | 197972 | 34886 |
| PHL | Western Pacific Region | 2016 | 188226 | 96016 | 48699 | 239847 | 44395 |
| PHL | Western Pacific Region | 2017 | 184405 | 93626 | 39235 | 235151 | 42880 |
| PHL | Western Pacific Region | 2018 | 214771 | 111467 | 45402 | 271039 | 55199 |
| PHL | Western Pacific Region | 2019 | 240296 | 126178 | 42669 | 302963 | 63511 |
| PHL | Western Pacific Region | 2020 | 161117 | 76964 | 18449 | 199643 | 38438 |
| MDA | European Region | 2013 | 3174 | 1177 | 134 | 4103 | 248 |
| MDA | European Region | 2014 | 2845 | 1099 | 114 | 3695 | 249 |
| MDA | European Region | 2015 | 2568 | 926 | 114 | 3276 | 218 |
| MDA | European Region | 2016 | 2523 | 945 | 103 | 3206 | 262 |
| MDA | European Region | 2017 | 2385 | 850 | 123 | 2981 | 254 |
| MDA | European Region | 2018 | 2180 | 747 | 95 | 2702 | 225 |
| MDA | European Region | 2019 | 1997 | 711 | 101 | 2456 | 252 |
| MDA | European Region | 2020 | 1309 | 402 | 56 | 1567 | 144 |
| RUS | European Region | 2014 | 69153 | 29280 | 3195 | 92327 | 6106 |
| RUS | European Region | 2015 | 67280 | 28446 | 3061 | 89443 | 6283 |
| RUS | European Region | 2016 | 62462 | 26672 | 2876 | 83289 | 5845 |
| RUS | European Region | 2017 | 57261 | 24272 | 2494 | 75763 | 5770 |
| RUS | European Region | 2018 | 53180 | 22591 | 2169 | 70105 | 5666 |
| RUS | European Region | 2019 | 49853 | 20874 | 2028 | 65187 | 5540 |
| RUS | European Region | 2020 | 39917 | 16774 | 1627 | 52191 | 4500 |
| SLE | African Region | 2013 | 4558 | 2729 | 103 | 6926 | 361 |
| SLE | African Region | 2014 | 4635 | 2683 | 135 | 6988 | 330 |
| SLE | African Region | 2018 | 9104 | 5674 | 2365 | 13757 | 1021 |
| SLE | African Region | 2019 | 9344 | 6095 | 2350 | 14283 | 1156 |
| SLE | African Region | 2020 | 8668 | 5489 | 1547 | 13198 | 959 |
| SOM | Eastern Mediterranean Region | 2014 | 6196 | 3924 | 2783 | 9029 | 1091 |
| SOM | Eastern Mediterranean Region | 2015 | 6675 | 4151 | 3156 | 9660 | 1166 |
| SOM | Eastern Mediterranean Region | 2016 | 6701 | 4099 | 3370 | 9637 | 1163 |
| SOM | Eastern Mediterranean Region | 2017 | 7664 | 4742 | 4079 | 11164 | 1242 |
| SOM | Eastern Mediterranean Region | 2018 | 7757 | 5173 | 3684 | 11713 | 1217 |
| SOM | Eastern Mediterranean Region | 2019 | 7898 | 5560 | 3460 | 12319 | 1139 |
| SOM | Eastern Mediterranean Region | 2020 | 7802 | 5709 | 3377 | 12236 | 1275 |
| ZAF | African Region | 2013 | 154786 | 120923 | 36671 | 263398 | 12311 |
| ZAF | African Region | 2014 | 157748 | 116441 | 31977 | 261379 | 12810 |
| ZAF | African Region | 2015 | 150761 | 107324 | 29137 | 245795 | 12290 |
| ZAF | African Region | 2016 | 128842 | 87642 | 20546 | 205789 | 10695 |
| ZAF | African Region | 2017 | 122945 | 81590 | 15628 | 193646 | 10889 |
| ZAF | African Region | 2018 | 126936 | 83464 | 17561 | 198634 | 11766 |
| ZAF | African Region | 2019 | 112911 | 80172 | 16461 | 181020 | 12063 |
| ZAF | African Region | 2020 | 106181 | 71335 | 13558 | 173830 | 3686 |
| TJK | European Region | 2014 | 3028 | 2445 | 334 | 5024 | 449 |
| TJK | European Region | 2015 | 3023 | 2543 | 328 | 5089 | 477 |
| TJK | European Region | 2016 | 3070 | 2506 | 389 | 5103 | 473 |
| TJK | European Region | 2017 | 3027 | 2515 | 353 | 5056 | 486 |
| TJK | European Region | 2018 | 2932 | 2448 | 346 | 4853 | 527 |
| TJK | European Region | 2019 | 3038 | 2313 | 404 | 4828 | 523 |
| TJK | European Region | 2020 | 2068 | 1840 | 240 | 3501 | 407 |
| THA | South-East Asia Region | 2015 | 22441 | 9110 | 109 |  |  |
| THA | South-East Asia Region | 2016 | 46870 | 22324 | 811 | 53113 | 16081 |
| THA | South-East Asia Region | 2017 | 50810 | 23493 | 759 | 57240 | 17063 |
| THA | South-East Asia Region | 2018 | 56221 | 25858 | 840 | 62469 | 19610 |
| THA | South-East Asia Region | 2019 | 57942 | 27480 | 874 | 65410 | 20012 |
| THA | South-East Asia Region | 2020 | 56707 | 27124 | 885 | 63197 | 20634 |
| UKR | European Region | 2013 | 23317 | 10189 | 638 | 30830 | 2676 |
| UKR | European Region | 2014 | 22245 | 8924 | 532 | 28699 | 2470 |
| UKR | European Region | 2015 | 21210 | 8373 | 568 | 27220 | 2363 |
| UKR | European Region | 2016 | 20807 | 7674 | 571 | 26185 | 2296 |
| UKR | European Region | 2017 | 18734 | 7884 | 611 | 24296 | 2322 |
| UKR | European Region | 2018 | 18326 | 7606 | 580 | 23742 | 2190 |
| UKR | European Region | 2019 | 17573 | 7221 | 585 | 22736 | 2058 |
| UKR | European Region | 2020 | 12150 | 5001 | 382 | 15747 | 1404 |
| TZA | African Region | 2014 | 33213 | 21895 | 6463 | 49490 | 5618 |
| TZA | African Region | 2015 | 34156 | 21036 | 5703 | 49282 | 5910 |
| TZA | African Region | 2016 | 36162 | 21973 | 6474 | 51700 | 6435 |
| TZA | African Region | 2017 | 36216 | 23238 | 8819 | 52006 | 7448 |
| TZA | African Region | 2018 | 39642 | 24538 | 10512 | 55633 | 8547 |
| TZA | African Region | 2019 | 42576 | 26392 | 12240 | 58626 | 10342 |
| TZA | African Region | 2020 | 43439 | 27762 | 13590 | 59574 | 11627 |
| UZB | European Region | 2013 | 11492 | 7360 | 1960 | 16588 | 2264 |
| UZB | European Region | 2014 | 9471 | 6961 | 1913 | 14195 | 2237 |
| UZB | European Region | 2015 | 8435 | 6085 | 1795 | 12563 | 1957 |
| UZB | European Region | 2016 | 7849 | 6191 | 2010 | 11931 | 2109 |
| UZB | European Region | 2017 | 8428 | 6425 | 1989 | 12654 | 2199 |
| UZB | European Region | 2018 | 7852 | 6532 | 2029 | 12115 | 2269 |
| UZB | European Region | 2019 | 7752 | 6330 | 2190 | 11701 | 2381 |
| UZB | European Region | 2020 | 5696 | 4682 | 1733 | 8591 | 1787 |
| VNM | Western Pacific Region | 2016 | 76979 | 23275 | 1691 | 83925 | 16329 |
| VNM | Western Pacific Region | 2017 | 73065 | 27817 | 1716 | 82332 | 18550 |
| VNM | Western Pacific Region | 2018 | 71043 | 26859 | 1656 | 79965 | 17937 |
| VNM | Western Pacific Region | 2019 | 72500 | 28216 | 1704 | 82384 | 18332 |
| VNM | Western Pacific Region | 2020 | 70754 | 27651 | 1396 | 79404 | 19001 |
| ZMB | African Region | 2013 | 22502 | 14982 | 3154 | 35703 | 1781 |
| ZMB | African Region | 2014 | 20472 | 13024 | 2726 | 31822 | 1674 |
| ZMB | African Region | 2015 | 21762 | 12703 | 2276 | 32847 | 1618 |
| ZMB | African Region | 2016 | 23197 | 12960 | 2169 | 34670 | 1487 |
| ZMB | African Region | 2017 | 22051 | 11820 | 2139 | 32193 | 1678 |
| ZMB | African Region | 2018 | 21759 | 11106 | 2206 | 31026 | 1839 |
| ZMB | African Region | 2019 | 22118 | 11559 | 2473 | 31607 | 2070 |
| ZMB | African Region | 2020 | 24602 | 12674 | 2724 | 34026 | 3250 |
| ZWE | African Region | 2013 | 17099 | 13233 | 2567 | 28399 | 1933 |
| ZWE | African Region | 2014 | 15723 | 11640 | 2290 | 25567 | 1796 |
| ZWE | African Region | 2015 | 14511 | 10659 | 1820 | 23514 | 1656 |
| ZWE | African Region | 2016 | 15049 | 10039 | 1530 | 23371 | 1717 |
| ZWE | African Region | 2017 | 14965 | 9484 | 1399 | 22614 | 1835 |
| ZWE | African Region | 2018 | 14554 | 9133 | 1517 | 21559 | 2128 |
| ZWE | African Region | 2019 | 12682 | 7155 | 1171 | 18226 | 1611 |
| ZWE | African Region | 2020 | 9622 | 5194 | 912 | 13690 | 1126 |

```
# Total notifications
#sum(WHO_not[year%in%c(2013:2019),men],na.rm=TRUE)
#sum(WHO_not[year%in%c(2013:2019),women],na.rm=TRUE)
#sum(WHO_not[year%in%c(2013:2019),children],na.rm=TRUE)
#sum(WHO_not[year%in%c(2013:2019),adults],na.rm=TRUE)
#sum(WHO_not[year%in%c(2013:2019),elderly],na.rm=TRUE)

#sum(WHO_not[year%in%c(2020),men],na.rm=TRUE)
#sum(WHO_not[year%in%c(2020),women],na.rm=TRUE)
#sum(WHO_not[year%in%c(2020),children],na.rm=TRUE)
#sum(WHO_not[year%in%c(2020),adults],na.rm=TRUE)
#sum(WHO_not[year%in%c(2020),elderly],na.rm=TRUE)
```

# 3. Linear models

We run country-specific linear models

```
mod<-list()
gof<-list()
countries<-unique(dat$iso3)
gr<-expand.grid(countries,2013:2020)
datPred<-data.frame(iso3=gr[,1],year=gr[,2],men=NA,women=NA,children=NA,adults=NA,elderly=NA,men_SE=NA,women_SE=NA,children_SE=NA,adults_SE=NA,elderly_SE=NA)
for(c in countries){
  for(var in c("men","women","children","adults","elderly")){
    mod[[paste(sep="_",c,var)]]<-glm(as.formula(paste(sep="",var," ~ year")), data=dat %>% dplyr::filter(iso3==c & year<2020),family=poisson)
    gof[[paste(sep="_",c,var)]]<-1-mod[[paste(sep="_",c,var)]]$deviance/mod[[paste(sep="_",c,var)]]$null.deviance
    tmpFit<-predict(mod[[paste(sep="_",c,var)]],newdata=datPred %>% dplyr::filter(iso3==c),se.fit=TRUE,type="response")
    datPred[datPred$iso3==c,var]<-tmpFit$fit
    tmpFit<-predict(mod[[paste(sep="_",c,var)]],newdata=datPred %>% dplyr::filter(iso3==c),se.fit=TRUE,type="link")
    datPred[datPred$iso3==c,paste(sep="_",var,"SE")]<-tmpFit$se.fit
  }
}
# Show data
datPred %>%
  dplyr::select(!contains("SE")) %>%
  knitr::kable(col.names=c("Country code","Year","Men (Expected)","Women (expected)","Children (expected)","Adults (expected)","Elderly (expected)")) %>%
  kableExtra::kable_styling(full_width = FALSE)
```

| Country code | Year | Men (Expected) | Women (expected) | Children (expected) | Adults (expected) | Elderly (expected) |
| --- | --- | --- | --- | --- | --- | --- |
| AZE | 2013 | 3113.303 | 1148.4123 | 1.932870e+02 | 4072.803 | 186.29383 |
| BGD | 2013 | 100753.833 | 64987.3691 | 6.685641e+03 | 143777.604 | 22349.28316 |
| BLR | 2013 | 3259.769 | 1230.7701 | 2.011674e+01 | 4003.023 | 492.42148 |
| BWA | 2013 | 3628.144 | 2663.5362 | 4.945617e+02 | 5822.708 | 473.69726 |
| BRA | 2013 | 48965.105 | 23356.8678 | 2.215928e+03 | 65341.344 | 6966.31057 |
| CMR | 2013 | 14311.678 | 10829.6561 | 1.604027e+03 | 23961.625 | 1181.20610 |
| CAF | 2013 | 4595.443 | 2832.9579 | 1.240456e+03 | 7133.226 | 272.78485 |
| CHN | 2013 | 579583.433 | 254756.1051 | 4.050537e+03 | 665724.062 | 169490.87228 |
| COG | 2013 | 3914.981 | 3191.4208 | 8.522540e+02 | 6633.329 | 470.79309 |
| PRK | 2013 | 59907.801 | 42507.7168 | 6.562807e+03 | 95428.270 | 6675.73216 |
| COD | 2013 | 42116.790 | 31219.2381 | 9.830887e+03 | 69453.783 | 3971.35320 |
| SWZ | 2013 | 3235.184 | 2594.2919 | 6.205233e+02 | 5522.506 | 305.66258 |
| ETH | 2013 | 68627.101 | 55338.3588 | 2.294536e+04 | 117885.148 | 6082.75014 |
| GAB | 2013 | 3004.032 | 2191.3802 | 5.109991e+02 | 4737.301 | 469.49372 |
| GIN | 2013 | 7045.191 | 3724.9717 | 1.999814e+02 | 10304.780 | 470.67079 |
| GNB | 2013 | 1331.747 | 801.5251 | 9.709450e+01 | 2043.605 | 89.59776 |
| IND | 2013 | 956871.742 | 441857.1902 | 8.291735e+04 | 1287594.830 | 109849.99356 |
| IDN | 2013 | 158561.374 | 109409.6958 | 2.086127e+04 | 247210.215 | 21148.04690 |
| KAZ | 2013 | 10245.019 | 6241.8308 | 4.725768e+02 | 15474.266 | 1053.90846 |
| KEN | 2013 | 47143.432 | 29885.1452 | 7.517334e+03 | 73354.701 | 3701.32707 |
| KGZ | 2013 | 4099.522 | 2948.9480 | 6.896792e+02 | 6668.675 | 464.75961 |
| LSO | 2013 | 4661.220 | 3412.3302 | 3.310932e+02 | 7677.809 | 479.28516 |
| LBR | 2013 | 2713.628 | 1419.3507 | 2.511182e+02 | 3999.078 | 132.92224 |
| MWI | 2013 | 8395.859 | 5924.0033 | 1.780961e+03 | 13383.684 | 944.62041 |
| MNG | 2013 | 2337.017 | 1783.2874 | 4.074439e+02 | 3924.119 | 197.08620 |
| MMR | 2013 | 63561.636 | 36067.2283 | 4.060423e+04 | 87941.414 | 11809.31936 |
| NAM | 2013 | 4963.391 | 3525.6234 | 1.077943e+03 | 7943.786 | 543.81761 |
| NPL | 2013 | 18172.432 | 9867.8329 | 2.204560e+03 | 25414.763 | 2938.27031 |
| NGA | 2013 | 52337.234 | 34377.9101 | 4.728366e+03 | 80815.327 | 5895.99626 |
| PAK | 2013 | 144628.380 | 146902.6859 | 2.834249e+04 | 263951.046 | 27965.36026 |
| PER | 2013 | 15539.038 | 11478.8116 | 1.750235e+03 | 23832.274 | 3078.88845 |
| PHL | 2013 | 75640.370 | 37189.9563 | 2.177264e+04 | 97998.116 | 15030.54566 |
| MDA | 2013 | 3098.148 | 1172.5262 | 1.261888e+02 | 4030.302 | 244.00000 |
| RUS | 2013 | 75672.760 | 32172.0847 | 3.651786e+03 | 101558.243 | 6358.93114 |
| SLE | 2013 | 4359.948 | 2545.1341 | 1.350429e+02 | 6591.695 | 316.83755 |
| SOM | 2013 | 5959.074 | 3528.9422 | 2.888635e+03 | 8371.544 | 1123.18482 |
| ZAF | 2013 | 160568.594 | 121276.9798 | 3.654614e+04 | 269568.848 | 12290.38989 |
| TJK | 2013 | 3046.356 | 2556.6359 | 3.236003e+02 | 5167.985 | 438.01914 |
| THA | 2013 | 22697.198 | 9819.0815 | 2.507811e+02 | 43168.956 | 12701.34691 |
| UKR | 2013 | 23388.922 | 9613.1648 | 5.857165e+02 | 30392.192 | 2610.66683 |
| TZA | 2013 | 31057.176 | 19938.9258 | 4.637898e+03 | 46581.547 | 4620.92227 |
| UZB | 2013 | 10382.133 | 6947.8125 | 1.866129e+03 | 15200.720 | 2132.51523 |
| VNM | 2013 | 80672.225 | 20941.9939 | 1.701225e+03 | 85354.200 | 15507.77302 |
| ZMB | 2013 | 21797.246 | 14257.1924 | 2.807771e+03 | 34416.145 | 1603.48166 |
| ZWE | 2013 | 16611.670 | 13002.4299 | 2.488640e+03 | 27764.826 | 1824.06238 |
| AZE | 2014 | 2941.937 | 1153.4287 | 1.903565e+02 | 3898.695 | 196.89346 |
| BGD | 2014 | 108679.256 | 71957.3294 | 7.415596e+03 | 155216.616 | 25638.85024 |
| BLR | 2014 | 2892.259 | 1089.1887 | 1.790463e+01 | 3516.870 | 463.98191 |
| BWA | 2014 | 3211.462 | 2333.5292 | 4.280198e+02 | 5099.143 | 445.26066 |
| BRA | 2014 | 50263.652 | 23473.7887 | 2.261234e+03 | 66537.623 | 7199.55607 |
| CMR | 2014 | 14237.868 | 10433.4119 | 1.528377e+03 | 23435.605 | 1237.31674 |
| CAF | 2014 | 4751.872 | 3039.3845 | 1.316833e+03 | 7485.856 | 291.72043 |
| CHN | 2014 | 567371.048 | 250414.0457 | 4.367600e+03 | 645085.700 | 172687.87337 |
| COG | 2014 | 4221.226 | 3387.4104 | 8.705390e+02 | 7095.736 | 511.66672 |
| PRK | 2014 | 59506.869 | 40305.4932 | 6.179505e+03 | 93529.702 | 6088.33392 |
| COD | 2014 | 48544.835 | 36290.2023 | 1.117982e+04 | 80057.780 | 4834.54846 |
| SWZ | 2014 | 2831.010 | 2221.1076 | 4.710685e+02 | 4787.771 | 264.56562 |
| ETH | 2014 | 66186.581 | 53059.4878 | 2.015328e+04 | 113310.699 | 5936.55966 |
| GAB | 2014 | 3044.548 | 2147.5706 | 4.729433e+02 | 4770.805 | 424.77959 |
| GIN | 2014 | 7354.068 | 3957.0418 | 2.724173e+02 | 10783.515 | 528.26282 |
| GNB | 2014 | 1332.812 | 795.8956 | 1.016208e+02 | 2038.157 | 90.52824 |
| IND | 2014 | 995129.840 | 476795.8867 | 9.007158e+04 | 1351614.455 | 119808.55457 |
| IDN | 2014 | 175039.880 | 121545.9817 | 2.579846e+04 | 271583.353 | 25060.49082 |
| KAZ | 2014 | 9556.667 | 5877.2557 | 4.459199e+02 | 14330.412 | 1101.88978 |
| KEN | 2014 | 47806.842 | 29285.2623 | 7.691387e+03 | 73108.730 | 3994.93959 |
| KGZ | 2014 | 3979.521 | 2857.0529 | 5.984863e+02 | 6367.616 | 511.61679 |
| LSO | 2014 | 4590.991 | 3173.9622 | 3.226708e+02 | 7249.241 | 546.93751 |
| LBR | 2014 | 2915.631 | 1616.4472 | 3.411620e+02 | 4368.525 | 164.37322 |
| MWI | 2014 | 8535.539 | 5839.6975 | 1.717852e+03 | 13355.500 | 1020.23901 |
| MNG | 2014 | 2301.206 | 1752.5625 | 4.058571e+02 | 3851.905 | 201.86001 |
| MMR | 2014 | 64819.515 | 36553.2424 | 3.721407e+04 | 88891.617 | 12527.00142 |
| NAM | 2014 | 4866.742 | 3375.8957 | 1.002072e+03 | 7703.852 | 539.41255 |
| NPL | 2014 | 18321.030 | 10018.8667 | 2.095121e+03 | 25229.168 | 3273.90142 |
| NGA | 2014 | 54121.359 | 34855.3076 | 5.244496e+03 | 82736.898 | 6239.88266 |
| PAK | 2014 | 147031.239 | 146564.5289 | 3.119215e+04 | 264002.159 | 29738.14530 |
| PER | 2014 | 16147.074 | 11309.5550 | 1.674830e+03 | 24177.240 | 3225.33054 |
| PHL | 2014 | 93445.125 | 46426.8604 | 2.509185e+04 | 120512.451 | 19464.22701 |
| MDA | 2014 | 2883.901 | 1077.1325 | 1.211351e+02 | 3716.818 | 244.00000 |
| RUS | 2014 | 70632.501 | 29995.0019 | 3.314516e+03 | 94437.534 | 6213.32937 |
| SLE | 2014 | 4986.703 | 2958.3716 | 2.247430e+02 | 7546.965 | 395.39619 |
| SOM | 2014 | 6270.350 | 3799.8453 | 3.029075e+03 | 8936.518 | 1136.21064 |
| ZAF | 2014 | 151755.084 | 112025.3497 | 3.125662e+04 | 251646.974 | 12134.33230 |
| TJK | 2014 | 3038.699 | 2529.0096 | 3.332232e+02 | 5116.918 | 451.87830 |
| THA | 2014 | 27003.309 | 11860.1382 | 3.172773e+02 | 46337.105 | 13745.00186 |
| UKR | 2014 | 22281.202 | 9125.3059 | 5.850000e+02 | 28891.950 | 2514.51717 |
| TZA | 2014 | 32616.265 | 20799.8635 | 5.433238e+03 | 48251.778 | 5247.65800 |
| UZB | 2014 | 9785.497 | 6812.5211 | 1.904016e+03 | 14442.779 | 2155.35979 |
| VNM | 2014 | 78990.647 | 22065.9416 | 1.699114e+03 | 84631.015 | 15985.56392 |
| ZMB | 2014 | 21857.929 | 13663.0102 | 2.678740e+03 | 33876.298 | 1645.52968 |
| ZWE | 2014 | 16020.779 | 11929.6357 | 2.192198e+03 | 26132.900 | 1819.64283 |
| AZE | 2015 | 2780.004 | 1158.4669 | 1.874704e+02 | 3732.030 | 208.09618 |
| BGD | 2015 | 117228.104 | 79674.8250 | 8.225250e+03 | 167565.721 | 29412.60517 |
| BLR | 2015 | 2566.182 | 963.8941 | 1.593577e+01 | 3089.759 | 437.18485 |
| BWA | 2015 | 2842.635 | 2044.4093 | 3.704308e+02 | 4465.492 | 418.53114 |
| BRA | 2015 | 51596.636 | 23591.2948 | 2.307465e+03 | 67755.803 | 7440.61109 |
| CMR | 2015 | 14164.438 | 10051.6657 | 1.456296e+03 | 22921.133 | 1296.09281 |
| CAF | 2015 | 4913.625 | 3260.8526 | 1.397911e+03 | 7855.917 | 311.97044 |
| CHN | 2015 | 555415.991 | 246145.9923 | 4.709481e+03 | 625087.156 | 175945.17751 |
| COG | 2015 | 4551.427 | 3595.4361 | 8.892164e+02 | 7590.378 | 556.08895 |
| PRK | 2015 | 59108.621 | 38217.3616 | 5.818590e+03 | 91668.907 | 5552.62091 |
| COD | 2015 | 55953.956 | 42184.8469 | 1.271385e+04 | 92280.765 | 5885.36392 |
| SWZ | 2015 | 2477.330 | 1901.6053 | 3.576103e+02 | 4150.789 | 228.99424 |
| ETH | 2015 | 63832.851 | 50874.4623 | 1.770096e+04 | 108913.758 | 5793.88267 |
| GAB | 2015 | 3085.610 | 2104.6367 | 4.377216e+02 | 4804.547 | 384.32399 |
| GIN | 2015 | 7676.488 | 4203.5701 | 3.710905e+02 | 11284.491 | 592.90191 |
| GNB | 2015 | 1333.878 | 790.3057 | 1.063581e+02 | 2032.723 | 91.46838 |
| IND | 2015 | 1034917.592 | 514497.2687 | 9.784309e+04 | 1418817.156 | 130669.91887 |
| IDN | 2015 | 193230.917 | 135028.4868 | 3.190413e+04 | 298359.506 | 29696.74708 |
| KAZ | 2015 | 8914.564 | 5533.9747 | 4.207666e+02 | 13271.111 | 1152.05555 |
| KEN | 2015 | 48479.588 | 28697.4208 | 7.869469e+03 | 72863.583 | 4311.84330 |
| KGZ | 2015 | 3863.033 | 2768.0215 | 5.193513e+02 | 6080.149 | 563.19813 |
| LSO | 2015 | 4521.821 | 2952.2453 | 3.144627e+02 | 6844.595 | 624.13916 |
| LBR | 2015 | 3132.671 | 1840.9132 | 4.634929e+02 | 4772.103 | 203.26589 |
| MWI | 2015 | 8677.543 | 5756.5914 | 1.656980e+03 | 13327.376 | 1101.91103 |
| MNG | 2015 | 2265.944 | 1722.3669 | 4.042765e+02 | 3781.019 | 206.74946 |
| MMR | 2015 | 66102.288 | 37045.8057 | 3.410696e+04 | 89852.087 | 13288.29883 |
| NAM | 2015 | 4771.976 | 3232.5267 | 9.315423e+02 | 7471.166 | 535.04316 |
| NPL | 2015 | 18470.843 | 10172.2123 | 1.991114e+03 | 25044.928 | 3647.87081 |
| NGA | 2015 | 55966.303 | 35339.3346 | 5.816964e+03 | 84704.159 | 6603.82638 |
| PAK | 2015 | 149474.018 | 146227.1503 | 3.432832e+04 | 264053.282 | 31623.31104 |
| PER | 2015 | 16778.903 | 11142.7942 | 1.602673e+03 | 24527.199 | 3378.73791 |
| PHL | 2015 | 115440.886 | 57957.9429 | 2.891705e+04 | 148199.286 | 25205.74713 |
| MDA | 2015 | 2684.470 | 989.4997 | 1.162837e+02 | 3427.717 | 244.00000 |
| RUS | 2015 | 65927.954 | 27965.2422 | 3.008396e+03 | 87816.090 | 6071.06148 |
| SLE | 2015 | 5703.556 | 3438.7038 | 3.740251e+02 | 8640.673 | 493.43313 |
| SOM | 2015 | 6597.885 | 4091.5446 | 3.176344e+03 | 9539.620 | 1149.38752 |
| ZAF | 2015 | 143425.342 | 103479.4814 | 2.673268e+04 | 234916.608 | 11980.25626 |
| TJK | 2015 | 3031.060 | 2501.6819 | 3.431322e+02 | 5066.356 | 466.17597 |
| THA | 2015 | 32126.375 | 14325.4618 | 4.014054e+02 | 49737.764 | 14874.41272 |
| UKR | 2015 | 21225.945 | 8662.2054 | 5.842844e+02 | 27465.763 | 2421.90865 |
| TZA | 2015 | 34253.621 | 21697.9754 | 6.364970e+03 | 49981.897 | 5959.39790 |
| UZB | 2015 | 9223.149 | 6679.8642 | 1.942672e+03 | 13722.630 | 2178.44907 |
| VNM | 2015 | 77344.121 | 23250.2111 | 1.697007e+03 | 83913.958 | 16478.07545 |
| ZMB | 2015 | 21918.781 | 13093.5912 | 2.555638e+03 | 33344.919 | 1688.68033 |
| ZWE | 2015 | 15450.905 | 10945.3547 | 1.931068e+03 | 24596.892 | 1815.23399 |
| AZE | 2016 | 2626.985 | 1163.5272 | 1.846281e+02 | 3572.489 | 219.93631 |
| BGD | 2016 | 126449.414 | 88220.0298 | 9.123304e+03 | 180897.327 | 33741.81506 |
| BLR | 2016 | 2276.868 | 853.0127 | 1.418341e+01 | 2714.518 | 411.93544 |
| BWA | 2016 | 2516.167 | 1791.1109 | 3.205904e+02 | 3910.582 | 393.40623 |
| BRA | 2016 | 52964.971 | 23709.3891 | 2.354642e+03 | 68996.285 | 7689.73708 |
| CMR | 2016 | 14091.387 | 9683.8872 | 1.387614e+03 | 22417.954 | 1357.66090 |
| CAF | 2016 | 5080.884 | 3498.4582 | 1.483982e+03 | 8244.273 | 333.62612 |
| CHN | 2016 | 543712.837 | 241950.6836 | 5.078124e+03 | 605708.594 | 179263.92216 |
| COG | 2016 | 4907.457 | 3816.2369 | 9.082945e+02 | 8119.500 | 604.36785 |
| PRK | 2016 | 58713.038 | 36237.4112 | 5.478754e+03 | 89845.133 | 5064.04532 |
| COD | 2016 | 64493.889 | 49036.9631 | 1.445837e+04 | 106369.919 | 7164.57984 |
| SWZ | 2016 | 2167.835 | 1628.0628 | 2.714789e+02 | 3598.552 | 198.20550 |
| ETH | 2016 | 61562.824 | 48779.4176 | 1.554704e+04 | 104687.437 | 5654.63473 |
| GAB | 2016 | 3127.225 | 2062.5612 | 4.051230e+02 | 4838.526 | 347.72135 |
| GIN | 2016 | 8013.043 | 4465.4574 | 5.055044e+02 | 11808.741 | 665.45033 |
| GNB | 2016 | 1334.945 | 784.7550 | 1.113162e+02 | 2027.304 | 92.41829 |
| IND | 2016 | 1076296.156 | 555179.7885 | 1.062851e+05 | 1489361.197 | 142515.93102 |
| IDN | 2016 | 213312.458 | 150006.5407 | 3.945481e+04 | 327775.594 | 35190.72286 |
| KAZ | 2016 | 8315.604 | 5210.7443 | 3.970322e+02 | 12290.113 | 1204.50522 |
| KEN | 2016 | 49161.800 | 28121.3790 | 8.051675e+03 | 72619.259 | 4653.88581 |
| KGZ | 2016 | 3749.955 | 2681.7644 | 4.506800e+02 | 5805.660 | 619.97991 |
| LSO | 2016 | 4453.693 | 2746.0165 | 3.064634e+02 | 6462.536 | 712.23803 |
| LBR | 2016 | 3365.867 | 2096.5495 | 6.296883e+02 | 5212.966 | 251.36101 |
| MWI | 2016 | 8821.910 | 5674.6680 | 1.598265e+03 | 13299.310 | 1190.12103 |
| MNG | 2016 | 2231.221 | 1692.6916 | 4.027020e+02 | 3711.439 | 211.75733 |
| MMR | 2016 | 67410.447 | 37545.0063 | 3.125927e+04 | 90822.935 | 14095.86220 |
| NAM | 2016 | 4679.055 | 3095.2463 | 8.659765e+02 | 7245.508 | 530.70917 |
| NPL | 2016 | 18621.881 | 10327.9049 | 1.892271e+03 | 24862.033 | 4064.55777 |
| NGA | 2016 | 57874.140 | 35830.0832 | 6.451921e+03 | 86718.197 | 6988.99726 |
| PAK | 2016 | 151957.383 | 145890.5483 | 3.777982e+04 | 264104.415 | 33627.98155 |
| PER | 2016 | 17435.454 | 10978.4922 | 1.533625e+03 | 24882.223 | 3539.44184 |
| PHL | 2016 | 142614.162 | 72353.0111 | 3.332541e+04 | 182246.965 | 32640.88977 |
| MDA | 2016 | 2498.830 | 908.9966 | 1.116267e+02 | 3161.103 | 244.00000 |
| RUS | 2016 | 61536.758 | 26072.8361 | 2.730548e+03 | 81658.906 | 5932.05113 |
| SLE | 2016 | 6523.458 | 3997.0246 | 6.224654e+02 | 9892.881 | 615.77794 |
| SOM | 2016 | 6942.530 | 4405.6365 | 3.330772e+03 | 10183.424 | 1162.71721 |
| ZAF | 2016 | 135552.814 | 95585.5357 | 2.286351e+04 | 219298.536 | 11828.13660 |
| TJK | 2016 | 3023.441 | 2474.6495 | 3.533358e+02 | 5016.294 | 480.92602 |
| THA | 2016 | 38221.389 | 17303.2432 | 5.078407e+02 | 53387.995 | 16096.62596 |
| UKR | 2016 | 20220.666 | 8222.6067 | 5.835697e+02 | 26109.978 | 2332.71086 |
| TZA | 2016 | 35973.173 | 22634.8667 | 7.456481e+03 | 51774.051 | 6767.67109 |
| UZB | 2016 | 8693.117 | 6549.7904 | 1.982112e+03 | 13038.389 | 2201.78570 |
| VNM | 2016 | 75731.915 | 24498.0398 | 1.694901e+03 | 83202.976 | 16985.76115 |
| ZMB | 2016 | 21979.803 | 12547.9032 | 2.438194e+03 | 32821.875 | 1732.96252 |
| ZWE | 2016 | 14901.303 | 10042.2840 | 1.701043e+03 | 23151.166 | 1810.83583 |
| AZE | 2017 | 2482.388 | 1168.6096 | 1.818289e+02 | 3419.769 | 232.45010 |
| BGD | 2017 | 136396.085 | 97681.7162 | 1.011941e+04 | 195289.601 | 38708.23672 |
| BLR | 2017 | 2020.171 | 754.8866 | 1.262376e+01 | 2384.850 | 388.14430 |
| BWA | 2017 | 2227.192 | 1569.1957 | 2.774558e+02 | 3424.629 | 369.78959 |
| BRA | 2017 | 54369.593 | 23828.0746 | 2.402783e+03 | 70259.478 | 7947.20430 |
| CMR | 2017 | 14018.713 | 9329.5653 | 1.322171e+03 | 21925.822 | 1422.15365 |
| CAF | 2017 | 5253.836 | 3753.3772 | 1.575352e+03 | 8651.827 | 356.78504 |
| CHN | 2017 | 532256.281 | 237826.8796 | 5.475622e+03 | 586930.794 | 182645.26622 |
| COG | 2017 | 5291.337 | 4050.5974 | 9.277819e+02 | 8685.508 | 656.83827 |
| PRK | 2017 | 58320.102 | 34360.0372 | 5.158766e+03 | 88057.643 | 4618.45955 |
| COD | 2017 | 74337.223 | 57002.0737 | 1.644226e+04 | 122610.163 | 8721.84032 |
| SWZ | 2017 | 1897.006 | 1393.8689 | 2.060924e+02 | 3119.788 | 171.55636 |
| ETH | 2017 | 59373.524 | 46770.6483 | 1.365522e+04 | 100625.115 | 5518.73341 |
| GAB | 2017 | 3169.402 | 2021.3268 | 3.749522e+02 | 4872.746 | 314.60470 |
| GIN | 2017 | 8364.354 | 4743.6606 | 6.886048e+02 | 12357.347 | 746.87590 |
| GNB | 2017 | 1336.012 | 779.2433 | 1.165054e+02 | 2021.899 | 93.37806 |
| IND | 2017 | 1119329.138 | 599079.1716 | 1.154556e+05 | 1563412.710 | 155435.85525 |
| IDN | 2017 | 235480.976 | 166646.0374 | 4.879250e+04 | 360091.895 | 41701.09850 |
| KAZ | 2017 | 7756.887 | 4906.3932 | 3.746365e+02 | 11381.630 | 1259.34277 |
| KEN | 2017 | 49853.613 | 27556.9000 | 8.238100e+03 | 72375.754 | 5023.06128 |
| KGZ | 2017 | 3640.186 | 2598.1953 | 3.910888e+02 | 5543.563 | 682.48644 |
| LSO | 2017 | 4386.591 | 2554.1937 | 2.986675e+02 | 6101.803 | 812.77227 |
| LBR | 2017 | 3616.422 | 2387.6844 | 8.554766e+02 | 5694.556 | 310.83602 |
| MWI | 2017 | 8968.678 | 5593.9105 | 1.541630e+03 | 13271.304 | 1285.39242 |
| MNG | 2017 | 2197.031 | 1663.5275 | 4.011337e+02 | 3643.138 | 216.88650 |
| MMR | 2017 | 68744.494 | 38050.9338 | 2.864934e+04 | 91804.273 | 14952.50323 |
| NAM | 2017 | 4587.943 | 2963.7961 | 8.050254e+02 | 7026.665 | 526.41029 |
| NPL | 2017 | 18774.155 | 10485.9804 | 1.798334e+03 | 24680.474 | 4528.84181 |
| NGA | 2017 | 59847.013 | 36327.6466 | 7.156188e+03 | 88780.123 | 7396.63339 |
| PAK | 2017 | 154482.006 | 145554.7212 | 4.157835e+04 | 264155.558 | 35759.73247 |
| PER | 2017 | 18117.696 | 10816.6130 | 1.467552e+03 | 25242.387 | 3707.78938 |
| PHL | 2017 | 176183.672 | 90323.3958 | 3.840581e+04 | 224116.844 | 42269.23644 |
| MDA | 2017 | 2326.028 | 835.0429 | 1.071561e+02 | 2915.226 | 244.00000 |
| RUS | 2017 | 57438.041 | 24308.4891 | 2.478362e+03 | 75933.430 | 5796.22372 |
| SLE | 2017 | 7461.224 | 4645.9964 | 1.035928e+03 | 11326.559 | 768.45768 |
| SOM | 2017 | 7305.177 | 4743.8400 | 3.492708e+03 | 10870.676 | 1176.20149 |
| ZAF | 2017 | 128112.404 | 88293.7807 | 1.955435e+04 | 204718.807 | 11677.94848 |
| TJK | 2017 | 3015.841 | 2447.9091 | 3.638429e+02 | 4966.727 | 496.14278 |
| THA | 2017 | 45472.748 | 20900.0052 | 6.424979e+02 | 57306.115 | 17419.26705 |
| UKR | 2017 | 19262.998 | 7805.3173 | 5.828558e+02 | 24821.117 | 2246.79818 |
| TZA | 2017 | 37779.048 | 23612.2117 | 8.735172e+03 | 53630.464 | 7685.57039 |
| UZB | 2017 | 8193.545 | 6422.2495 | 2.022354e+03 | 12388.267 | 2225.37232 |
| VNM | 2017 | 74153.316 | 25812.8389 | 1.692799e+03 | 82498.019 | 17509.08853 |
| ZMB | 2017 | 22040.995 | 12024.9573 | 2.326146e+03 | 32307.035 | 1778.40591 |
| ZWE | 2017 | 14371.251 | 9213.7232 | 1.498418e+03 | 21790.416 | 1806.44833 |
| AZE | 2018 | 2345.750 | 1173.7142 | 1.790721e+02 | 3273.578 | 245.67591 |
| BGD | 2018 | 147125.173 | 108158.1781 | 1.122427e+04 | 210826.932 | 44405.66068 |
| BLR | 2018 | 1792.415 | 668.0483 | 1.123560e+01 | 2095.218 | 365.72721 |
| BWA | 2018 | 1971.406 | 1374.7753 | 2.401248e+02 | 2999.064 | 347.59069 |
| BRA | 2018 | 55811.466 | 23947.3542 | 2.451909e+03 | 71545.798 | 8213.29202 |
| CMR | 2018 | 13946.413 | 8988.2076 | 1.259815e+03 | 21444.493 | 1489.70999 |
| CAF | 2018 | 5432.676 | 4026.8711 | 1.672349e+03 | 9079.528 | 381.55157 |
| CHN | 2018 | 521041.126 | 233773.3617 | 5.904236e+03 | 568735.132 | 186090.39048 |
| COG | 2018 | 5705.246 | 4299.3503 | 9.476874e+02 | 9290.972 | 713.86410 |
| PRK | 2018 | 57929.796 | 32579.9256 | 4.857468e+03 | 86305.715 | 4212.08090 |
| COD | 2018 | 85682.889 | 66260.9631 | 1.869837e+04 | 141329.919 | 10617.57984 |
| SWZ | 2018 | 1660.011 | 1193.3634 | 1.564545e+02 | 2704.720 | 148.49026 |
| ETH | 2018 | 57262.079 | 44844.6014 | 1.199360e+04 | 96720.429 | 5386.09830 |
| GAB | 2018 | 3212.148 | 1980.9168 | 3.470282e+02 | 4907.208 | 284.64205 |
| GIN | 2018 | 8731.067 | 5039.1962 | 9.380266e+02 | 12931.439 | 838.26482 |
| GNB | 2018 | 1337.081 | 773.7703 | 1.219366e+02 | 2016.508 | 94.34780 |
| IND | 2018 | 1164082.685 | 646449.7832 | 1.254172e+05 | 1641146.088 | 169527.04812 |
| IDN | 2018 | 259953.359 | 185131.2726 | 6.034013e+04 | 395594.349 | 49415.91065 |
| KAZ | 2018 | 7235.710 | 4619.8187 | 3.535042e+02 | 10540.303 | 1316.67691 |
| KEN | 2018 | 50555.162 | 27003.7518 | 8.428841e+03 | 72133.066 | 5421.52206 |
| KGZ | 2018 | 3533.631 | 2517.2304 | 3.393770e+02 | 5293.297 | 751.29489 |
| LSO | 2018 | 4320.500 | 2375.7708 | 2.910699e+02 | 5761.206 | 927.49720 |
| LBR | 2018 | 3885.629 | 2719.2474 | 1.162226e+03 | 6220.637 | 384.38352 |
| MWI | 2018 | 9117.889 | 5514.3023 | 1.487002e+03 | 13243.357 | 1388.29045 |
| MNG | 2018 | 2163.365 | 1634.8660 | 3.995715e+02 | 3576.095 | 222.13992 |
| MMR | 2018 | 70104.942 | 38563.6788 | 2.625733e+04 | 92796.214 | 15861.20449 |
| NAM | 2018 | 4498.605 | 2837.9283 | 7.483643e+02 | 6814.432 | 522.14622 |
| NPL | 2018 | 18927.673 | 10646.4755 | 1.709061e+03 | 24500.241 | 5046.15984 |
| NGA | 2018 | 61887.139 | 36832.1196 | 7.937329e+03 | 90891.075 | 7828.04506 |
| PAK | 2018 | 157048.573 | 145219.6671 | 4.575880e+04 | 264206.711 | 38026.61973 |
| PER | 2018 | 18826.634 | 10657.1206 | 1.404326e+03 | 25607.763 | 3884.14408 |
| PHL | 2018 | 217655.006 | 112757.1017 | 4.426071e+04 | 275606.015 | 54737.73422 |
| MDA | 2018 | 2165.175 | 767.1060 | 1.028646e+02 | 2688.475 | 244.00000 |
| RUS | 2018 | 53612.324 | 22663.5353 | 2.249466e+03 | 70609.393 | 5663.50639 |
| SLE | 2018 | 8533.796 | 5400.3377 | 1.724028e+03 | 12968.007 | 958.99375 |
| SOM | 2018 | 7686.768 | 5108.0060 | 3.662518e+03 | 11604.309 | 1189.84215 |
| ZAF | 2018 | 121080.394 | 81558.2782 | 1.672414e+04 | 191108.389 | 11529.66738 |
| TJK | 2018 | 3008.260 | 2421.4577 | 3.746624e+02 | 4917.649 | 511.84100 |
| THA | 2018 | 54099.835 | 25244.4130 | 8.128604e+02 | 61511.784 | 18850.58802 |
| UKR | 2018 | 18350.686 | 7409.2049 | 5.821429e+02 | 23595.879 | 2164.04963 |
| TZA | 2018 | 39675.578 | 24631.7572 | 1.023314e+04 | 55553.441 | 8727.96437 |
| UZB | 2018 | 7722.682 | 6297.1922 | 2.063412e+03 | 11770.560 | 2249.21161 |
| VNM | 2018 | 72607.622 | 27198.2028 | 1.690699e+03 | 81799.034 | 18048.53950 |
| ZMB | 2018 | 22102.357 | 11523.8057 | 2.219248e+03 | 31800.272 | 1825.04097 |
| ZWE | 2018 | 13860.053 | 8453.5246 | 1.319930e+03 | 20509.645 | 1802.07145 |
| AZE | 2019 | 2216.633 | 1178.8411 | 1.763571e+02 | 3133.635 | 259.65422 |
| BGD | 2019 | 158698.224 | 119758.2509 | 1.244977e+04 | 227600.419 | 50941.68237 |
| BLR | 2019 | 1590.336 | 591.1995 | 1.000009e+01 | 1840.761 | 344.60480 |
| BWA | 2019 | 1744.995 | 1204.4433 | 2.078167e+02 | 2626.382 | 326.72442 |
| BRA | 2019 | 57291.577 | 24067.2309 | 2.502039e+03 | 72855.669 | 8488.28887 |
| CMR | 2019 | 13874.487 | 8659.3398 | 1.200400e+03 | 20973.731 | 1560.47545 |
| CAF | 2019 | 5617.604 | 4320.2935 | 1.775317e+03 | 9528.372 | 408.03728 |
| CHN | 2019 | 510062.284 | 229788.9319 | 6.366400e+03 | 551103.561 | 189600.49798 |
| COG | 2019 | 6151.532 | 4563.3794 | 9.680199e+02 | 9938.642 | 775.84083 |
| PRK | 2019 | 57542.102 | 30892.0372 | 4.573766e+03 | 84588.643 | 3841.45955 |
| COD | 2019 | 98760.179 | 77023.7809 | 2.126405e+04 | 162907.752 | 12925.36868 |
| SWZ | 2019 | 1452.625 | 1021.7002 | 1.187720e+02 | 2344.874 | 128.52544 |
| ETH | 2019 | 55225.723 | 42997.8704 | 1.053418e+04 | 92967.262 | 5256.65089 |
| GAB | 2019 | 3255.470 | 1941.3147 | 3.211839e+02 | 4941.914 | 257.53302 |
| GIN | 2019 | 9113.857 | 5353.1440 | 1.277792e+03 | 13532.202 | 940.83625 |
| GNB | 2019 | 1338.150 | 768.3358 | 1.276210e+02 | 2011.132 | 95.32761 |
| IND | 2019 | 1210625.589 | 697566.1014 | 1.362384e+05 | 1722744.394 | 184895.69217 |
| IDN | 2019 | 286969.036 | 205666.9850 | 7.462070e+04 | 434597.088 | 58557.98320 |
| KAZ | 2019 | 6749.550 | 4349.9826 | 3.335639e+02 | 9761.166 | 1376.62130 |
| KEN | 2019 | 51266.582 | 26461.7070 | 8.623998e+03 | 71891.191 | 5851.59126 |
| KGZ | 2019 | 3430.195 | 2438.7885 | 2.945028e+02 | 5054.331 | 827.04063 |
| LSO | 2019 | 4255.405 | 2209.8115 | 2.836657e+02 | 5439.621 | 1058.41584 |
| LBR | 2019 | 4174.876 | 3096.8525 | 1.578967e+03 | 6795.320 | 475.33323 |
| MWI | 2019 | 9269.581 | 5435.8270 | 1.434310e+03 | 13215.469 | 1499.42566 |
| MNG | 2019 | 2130.215 | 1606.6982 | 3.980153e+02 | 3510.285 | 227.52058 |
| MMR | 2019 | 71492.313 | 39083.3331 | 2.406503e+04 | 93798.873 | 16825.12982 |
| NAM | 2019 | 4411.007 | 2717.4059 | 6.956913e+02 | 6608.610 | 517.91670 |
| NPL | 2019 | 19082.447 | 10809.4270 | 1.624219e+03 | 24321.324 | 5622.56978 |
| NGA | 2019 | 63996.811 | 37343.5981 | 8.803736e+03 | 93052.221 | 8284.61900 |
| PAK | 2019 | 159657.781 | 144885.3842 | 5.035956e+04 | 264257.874 | 40437.20990 |
| PER | 2019 | 19563.313 | 10499.9800 | 1.343823e+03 | 25978.429 | 4068.88679 |
| PHL | 2019 | 268888.150 | 140762.6881 | 5.100817e+04 | 338924.438 | 70884.16542 |
| MDA | 2019 | 2015.446 | 704.6962 | 9.874498e+01 | 2479.360 | 244.00000 |
| RUS | 2019 | 50041.422 | 21129.8954 | 2.041711e+03 | 65658.648 | 5533.82791 |
| SLE | 2019 | 9760.553 | 6277.1567 | 2.869186e+03 | 14847.333 | 1196.77251 |
| SOM | 2019 | 8088.291 | 5500.1277 | 3.840583e+03 | 12387.454 | 1203.64100 |
| ZAF | 2019 | 114434.367 | 75336.5944 | 1.430357e+04 | 178402.839 | 11383.26909 |
| TJK | 2019 | 3000.699 | 2395.2922 | 3.858036e+02 | 4869.056 | 528.03593 |
| THA | 2019 | 64363.652 | 30491.8769 | 1.028396e+03 | 66026.105 | 20399.51897 |
| UKR | 2019 | 17481.581 | 7033.1949 | 5.814308e+02 | 22431.121 | 2084.34868 |
| TZA | 2019 | 41667.316 | 25695.3255 | 1.198800e+04 | 57545.369 | 9911.73826 |
| UZB | 2019 | 7278.878 | 6174.5700 | 2.105304e+03 | 11183.654 | 2273.30628 |
| VNM | 2019 | 71094.147 | 28657.9185 | 1.688601e+03 | 81105.971 | 18604.61083 |
| ZMB | 2019 | 22163.889 | 11043.5400 | 2.117263e+03 | 31301.457 | 1872.89893 |
| ZWE | 2019 | 13367.039 | 7756.0478 | 1.162702e+03 | 19304.155 | 1797.70519 |
| AZE | 2020 | 2094.623 | 1183.9903 | 1.736833e+02 | 2999.676 | 274.42786 |
| BGD | 2020 | 171181.627 | 132602.4431 | 1.380906e+04 | 245708.413 | 58439.73409 |
| BLR | 2020 | 1411.039 | 523.1910 | 8.900447e+00 | 1617.207 | 324.70231 |
| BWA | 2020 | 1544.588 | 1055.2151 | 1.798555e+02 | 2300.011 | 307.11077 |
| BRA | 2020 | 58810.940 | 24187.7077 | 2.553194e+03 | 74189.520 | 8772.49314 |
| CMR | 2020 | 13802.931 | 8342.5049 | 1.143786e+03 | 20513.303 | 1634.60247 |
| CAF | 2020 | 5808.826 | 4635.0965 | 1.884625e+03 | 9999.406 | 436.36151 |
| CHN | 2020 | 499314.778 | 225872.4127 | 6.864740e+03 | 534018.592 | 193176.81445 |
| COG | 2020 | 6632.729 | 4843.6230 | 9.887887e+02 | 10631.461 | 843.19830 |
| PRK | 2020 | 57157.003 | 29291.5944 | 4.306635e+03 | 82905.732 | 3503.44920 |
| COD | 2020 | 113833.381 | 89534.8112 | 2.418177e+04 | 187780.024 | 15734.76801 |
| SWZ | 2020 | 1271.147 | 874.7304 | 9.016542e+01 | 2032.903 | 111.24494 |
| ETH | 2020 | 53261.783 | 41227.1891 | 9.252340e+03 | 89359.733 | 5130.31456 |
| GAB | 2020 | 3299.376 | 1902.5043 | 2.972643e+02 | 4976.865 | 233.00582 |
| GIN | 2020 | 9513.430 | 5686.6511 | 1.740626e+03 | 14160.876 | 1055.95847 |
| GNB | 2020 | 1339.220 | 762.9394 | 1.335703e+02 | 2005.771 | 96.31759 |
| IND | 2020 | 1259029.393 | 752724.3081 | 1.479933e+05 | 1808399.794 | 201657.59602 |
| IDN | 2020 | 316792.321 | 228480.6243 | 9.228103e+04 | 477445.216 | 69391.36305 |
| KAZ | 2020 | 6296.054 | 4095.9072 | 3.147484e+02 | 9039.622 | 1439.29478 |
| KEN | 2020 | 51988.014 | 25930.5425 | 8.823673e+03 | 71650.128 | 6315.77625 |
| KGZ | 2020 | 3329.786 | 2362.7910 | 2.555622e+02 | 4826.152 | 910.42308 |
| LSO | 2020 | 4191.290 | 2055.4453 | 2.764497e+02 | 5135.986 | 1207.81398 |
| LBR | 2020 | 4485.654 | 3526.8933 | 2.145140e+03 | 7423.093 | 587.80273 |
| MWI | 2020 | 9423.797 | 5358.4685 | 1.383485e+03 | 13187.640 | 1619.45744 |
| MNG | 2020 | 2097.573 | 1579.0158 | 3.964652e+02 | 3445.687 | 233.03157 |
| MMR | 2020 | 72907.140 | 39609.9899 | 2.205577e+04 | 94812.366 | 17847.63531 |
| NAM | 2020 | 4325.114 | 2602.0019 | 6.467256e+02 | 6409.004 | 513.72144 |
| NPL | 2020 | 19238.487 | 10974.8726 | 1.543589e+03 | 24143.714 | 6264.82155 |
| NGA | 2020 | 66178.400 | 37862.1794 | 9.764717e+03 | 95264.753 | 8767.82279 |
| PAK | 2020 | 162310.339 | 144551.8709 | 5.542290e+04 | 264309.046 | 43000.61263 |
| PER | 2020 | 20328.817 | 10345.1564 | 1.285927e+03 | 26354.459 | 4262.41648 |
| PHL | 2020 | 332180.907 | 175724.0480 | 5.878428e+04 | 416789.796 | 91793.43973 |
| MDA | 2020 | 1876.072 | 647.3639 | 9.479034e+01 | 2286.511 | 244.00000 |
| RUS | 2020 | 46708.364 | 19700.0368 | 1.853144e+03 | 61055.022 | 5407.11870 |
| SLE | 2020 | 11163.660 | 7296.3392 | 4.774999e+03 | 16999.012 | 1493.50758 |
| SOM | 2020 | 8510.788 | 5922.3510 | 4.027306e+03 | 13223.450 | 1217.59988 |
| ZAF | 2020 | 108153.136 | 69589.5325 | 1.223333e+04 | 166541.998 | 11238.72969 |
| TJK | 2020 | 2993.156 | 2369.4094 | 3.972762e+02 | 4820.943 | 544.74326 |
| THA | 2020 | 76574.720 | 36830.1120 | 1.301081e+03 | 70871.731 | 22075.72379 |
| UKR | 2020 | 16653.639 | 6676.2670 | 5.807195e+02 | 21323.859 | 2007.58307 |
| TZA | 2020 | 43759.040 | 26804.8172 | 1.404378e+04 | 59608.719 | 11256.06741 |
| UZB | 2020 | 6860.579 | 6054.3356 | 2.148047e+03 | 10626.012 | 2297.65906 |
| VNM | 2020 | 69612.220 | 30195.9765 | 1.686507e+03 | 80418.781 | 19177.81459 |
| ZMB | 2020 | 22225.594 | 10583.2899 | 2.019964e+03 | 30810.466 | 1922.01188 |
| ZWE | 2020 | 12891.562 | 7116.1180 | 1.024204e+03 | 18169.519 | 1793.34950 |

# 4. Plot models

We get the following predictions for mean number of notified
cases

```
datObsLong<-dat %>%
  dplyr::select(iso3,year,men,women,adults,children,elderly) %>%
  tidyr::pivot_longer(cols=c(men,women,adults,children,elderly),names_to="group",values_to="observed")
datExpLong<-datPred %>%
  dplyr::select(iso3,year,men,women,adults,children,elderly) %>%
  tidyr::pivot_longer(cols=c(men,women,adults,children,elderly),names_to="group",values_to="expected")
datExpSELong<-datPred %>%
  dplyr::select(iso3,year,men_SE,women_SE,adults_SE,children_SE,elderly_SE) %>%
  tidyr::pivot_longer(cols=c(men_SE,women_SE,adults_SE,children_SE,elderly_SE),names_to="group",values_to="SE")
datExpSELong$group<-gsub(pattern="_SE",replacement="",datExpSELong$group)
datFullLong<-datObsLong %>%
  mutate(
    expected=datExpLong$expected[match(paste(sep="_",iso3,year,group),paste(sep="_",datExpLong$iso3,datExpLong$year,datExpLong$group))],
    expectedLow=exp(log(datExpLong$expected[match(paste(sep="_",iso3,year,group),paste(sep="_",datExpLong$iso3,datExpLong$year,datExpLong$group))])-qnorm(0.975)*datExpSELong$SE[match(paste(sep="_",iso3,year,group),paste(sep="_",datExpLong$iso3,datExpLong$year,datExpLong$group))]),
    expectedUpp=exp(log(datExpLong$expected[match(paste(sep="_",iso3,year,group),paste(sep="_",datExpLong$iso3,datExpLong$year,datExpLong$group))])+qnorm(0.975)*datExpSELong$SE[match(paste(sep="_",iso3,year,group),paste(sep="_",datExpLong$iso3,datExpLong$year,datExpLong$group))]),
      annot=paste(sep="",iso3," ",group)
)
for(j in 1:nrow(datFullLong)){datFullLong$annot[j]<-paste(sep="",datFullLong$annot[j]," (pseudo R2 ",format(nsmall=2,round(digits=2,gof[[paste(sep="_",datFullLong$iso3[j],datFullLong$group[j])]])),")")}
```

```
  datFullLong %>%
  ggplot(mapping=aes(x=year)) +
  geom_ribbon(mapping=aes(ymin=expectedLow,ymax=expectedUpp),fill="orange",alpha=0.5) +
  geom_line(mapping=aes(y=expected),col="orange",lwd=3) +
  geom_point(mapping=aes(y=observed),col="steelblue",size=10) + 
  facet_wrap(~paste(sep=" ",iso3,group),ncol = 5,scales = "free_y") +
  theme(axis.text.x=element_text(size=25),axis.text.y=element_text(size=25),axis.title.x=element_text(size=25),axis.title.y=element_text(size=25),strip.text = element_text(size=40))
```

# 5. Reformat data

We reformat the data for further analysis

```
datPredLong<-datPred %>%
  dplyr::filter(year==2020) %>%
  dplyr::select(!contains("SE")) %>%
  tidyr::pivot_longer(cols=c(men,women,children,adults,elderly),names_to="group",values_to="expected")
datPredSELong<-datPred %>%
  dplyr::filter(year==2020) %>%
  dplyr::select(c(iso3,year,contains("SE"))) %>%
  tidyr::pivot_longer(cols=c(men_SE,women_SE,children_SE,adults_SE,elderly_SE),names_to="group",values_to="expected")
datLong<-dat %>% 
  dplyr::filter(year==2020) %>%
  dplyr::select(iso3,men,women,children,adults,elderly) %>%
  tidyr::pivot_longer(cols=c(men,women,children,adults,elderly),names_to="group",values_to="observed") %>%
  dplyr::mutate(
    expected=datPredLong$expected[match(paste(sep="_",iso3,group),paste(sep="_",datPredLong$iso3,datPredLong$group))],
    expected_SE=datPredSELong$expected[match(paste(sep="_",iso3,group,"SE"),paste(sep="_",datPredSELong$iso3,datPredSELong$group))]
  )
```

# 6. Compute risks

We create a function to compute relative risks and standard
errors

```
analysisFun<-function(refDat,compDat,B=1e4){
  # refDat = data frame with observed, expected and expected SEs for the reference group for each country
  # compDat = same dataframe but including all comparator groups
  # B = number of parametric bootstrap replicates to sample
  # filter out countries with complete data
  allCountries<-intersect(refDat$iso3[!is.na(refDat$observed) & !is.na(refDat$expected) & !is.na(refDat$expected_SE)],compDat$iso3[!is.na(compDat$observed) & !is.na(compDat$expected) & !is.na(compDat$expected_SE)])
  refDat<-refDat %>%
    dplyr::filter(iso3 %in% allCountries) %>%
    dplyr::mutate(
      observedCC=case_when(
        observed>expected~expected-0.5, # "continuity correction" for cases where observed exceeds expected; should be near 0 risk, but not quite
        TRUE~as.numeric(observed)
      ),
      moreObsThanExp=factor(levels=c("no","yes"),case_when(
        observed>expected~"yes",
        TRUE~"no"
      ))) %>%
    dplyr::mutate(risk=(expected-observedCC)/expected)
  compDat<-compDat %>%
    dplyr::filter(iso3 %in% allCountries) %>%
    dplyr::mutate(
      RR=NA,
      RR_low=NA,
      RR_upp=NA,
      RR_logSE=NA,
      observedCC=case_when(
        observed>expected~expected-0.5, # "continuity correction" for cases where observed exceeds expected; should be near 0 risk, but not quite
        TRUE~as.numeric(observed)
      ),
      moreObsThanExp=factor(levels=c("no","yes"),case_when(
        observed>expected~"yes",
        TRUE~"no"
      ))) %>%
    dplyr::mutate(risk=(expected-observedCC)/expected)
  # compute RRs
  for(c in allCountries){
    idx<-which(compDat$iso3==c) # will be a number of groups for that country
    R0<-refDat$risk[refDat$iso3==c] # should be only 1 such value
    if(refDat$moreObsThanExp[refDat$iso3==c]=="no"){
      compDat$RR[idx]<-compDat$risk[idx]/R0
    }else{
      idx<-which(compDat$iso3==c & compDat$moreObsThanExp=="no")
      compDat$RR[idx]<-compDat$risk[idx]/R0
      idx<-which(compDat$iso3==c & compDat$moreObsThanExp!="no")
      compDat$RR[idx]<-NA # cannot compute RRs where both the reference and the comparator have more observed notifications than expected
    }
  }
  # parametric boostrapping using the estimated SEs
  bootRR<-compDat %>% dplyr::select(iso3,group)
  for(b in 1:B){
    refDat_BS<-refDat
    refDat_BS$expected<-exp(rnorm(n=nrow(refDat),mean=log(refDat$expected),sd=refDat$expected_SE))
    refDat_BS<-refDat_BS %>%
      dplyr::mutate(
        missed=case_when(
          expected-observed>0~expected-observed,
          TRUE~0),
        observedCC=case_when(
          observed>expected~expected-0.5, # "continuity correction" for cases where observed exceeds expected; should be near 0 risk, but not quite
          TRUE~as.numeric(observed)
        ),
        moreObsThanExp=case_when(
          observed>expected~"yes",
          TRUE~"no"
        )) %>%
      dplyr::mutate(risk=(expected-observedCC)/expected)
    compDat_BS<-compDat
    compDat_BS$expected<-exp(rnorm(n=nrow(compDat),mean=log(compDat$expected),sd=compDat$expected_SE))
    compDat_BS<-compDat_BS %>%
      dplyr::mutate(
        missed=case_when(
          expected-observed>0~expected-observed,
          TRUE~0),
        observedCC=case_when(
          observed>expected~expected-0.5, # "continuity correction" for cases where observed exceeds expected; should be near 0 risk, but not quite
          TRUE~as.numeric(observed)
        ),
        moreObsThanExp=case_when(
          observed>expected~"yes",
          TRUE~"no"
        )) %>%
      dplyr::mutate(risk=(expected-observedCC)/expected)   
    for(c in allCountries){
      idx<-which(compDat_BS$iso3==c) # will be a number of groups for that country
      R0<-refDat_BS$risk[refDat_BS$iso3==c] # should be only 1 such value
      if(refDat_BS$moreObsThanExp[refDat_BS$iso3==c]=="no"){
        compDat_BS$RR[idx]<-compDat_BS$risk[idx]/R0
      }else{
        idx<-which(compDat_BS$iso3==c & compDat_BS$moreObsThanExp=="no")
        compDat_BS$RR[idx]<-compDat_BS$risk[idx]/R0
        idx<-which(compDat_BS$iso3==c & compDat_BS$moreObsThanExp!="no")
        compDat_BS$RR[idx]<-NA # cannot compute RRs where both the reference and the comparator have more observed notifications than expected
      }
    }
    bootRR[,paste(sep="_","RR",b)]<-compDat_BS$RR
  }
  compDat[,c("RR_low","RR_upp")]<-t(apply(X=bootRR %>% dplyr::select(contains("RR_")),MARGIN=1,FUN=quantile,probs=c(0.025,0.975),na.rm=TRUE))
  logsd<-function(x){
    x<-x[!is.na(x)] # removes NaNs due to 0/0
    # x[x<1e-4]<-1e-4 # avoids -Inf values when logged
    # x[x>1e4]<-1e4 # avoids +Inf values when logged
    res<-sd(log(x),na.rm=TRUE)
    return(res)
  }
  compDat[,"RR_logSE"]<-apply(X=bootRR %>% dplyr::select(contains("RR_")),MARGIN=1,FUN=logsd)
  return(list=list(compDat=compDat,reference=refDat,bootRR=bootRR))
}
plotFun<-function(analysisObj,maxRR=25){
  # analysisObj = output from analysisFun()
   g1<-analysisObj$compDat %>%
    mutate(iso3=paste(sep="",iso3," (",format(nsmall=2,round(digits=1,100*analysisObj$reference$risk[match(iso3,analysisObj$reference$iso3)])),"%)")) %>%
    ggplot() +
    geom_segment(mapping=aes(y=group,yend=group,x=RR_low,xend=RR_upp)) +
    geom_point(mapping=aes(x=RR,y=group)) +
    geom_vline(xintercept=1,lty=2,col="darkgrey") +
    facet_wrap(~iso3,scales="free_x") +
    labs(caption="Estimated risk ratios with 95% confidence intervals obtained from parametric bootstrapping from fitted linear models.\nRisks of being missed in the reference group are shown in brackets in the panel titles.",title=paste(sep="","Reference group: ",unique(analysisObj$reference$group,".")))
  tmp<-analysisObj$compDat %>%
    mutate(iso3=paste(sep="",iso3," (",format(nsmall=2,round(digits=1,100*analysisObj$reference$risk[match(iso3,analysisObj$reference$iso3)])),"%)"))
  g2<-analysisObj$bootRR %>%
    mutate(iso3=paste(sep="",iso3," (",format(nsmall=2,round(digits=1,100*analysisObj$reference$risk[match(iso3,analysisObj$reference$iso3)])),"%)")) %>%
    pivot_longer(cols=contains("RR_"),names_to = "bootNum",values_to="RR") %>%
    dplyr::filter(abs(RR)<maxRR) %>%
    suppressMessages(
    ggplot(mapping=aes(x = RR, y = group, fill = group)) +
    geom_segment(data=tmp,mapping=aes(y=group,yend=group,x=RR_low,xend=RR_upp),col="grey50",size=1,position = position_nudge(y = -0.1)) +
    geom_point(data=tmp,mapping=aes(x=RR,y=group),col="grey50",size=1.5,position = position_nudge(y = -0.1)) +
    geom_density_ridges(alpha=0.75) +
    geom_vline(xintercept=1,lty=2,col="darkgrey") +
    scale_fill_manual(values=viridis(length(unique(analysisObj$bootRR$group)))) +
    theme_ridges() +
    theme(legend.position = "none",axis.title.x=element_text(hjust=0.5)) +
    ylab("") +
    xlab("relative risk") +
    facet_wrap(~iso3,scales="free_x") +
    labs(caption="Estimated risk ratios with 95% confidence intervals obtained from parametric bootstrapping from fitted linear models.\nDensity histograms show the empirical distributions for the risk ratio computed from the parametric boostrapped samples.\nGrey bars below each histogram show the derived 95% confidence intervals for the risk ratio estimates.\nRisks of being missed in the reference group are shown in brackets in the panel titles.",title=paste(sep="","Reference group: ",unique(analysisObj$reference$group,"."))))
  return(list(forestPlot=g1,ridgePlot=g2))
}
```

# 7. Missed cases

We show missed cases

```
refDat<-datLong
refDat<-refDat %>%
  dplyr::mutate(
    missed=case_when(
      expected-observed>0~round(expected-observed),
      TRUE~0),
    missedLow=case_when(
      exp(log(expected)-qnorm(0.975)*expected_SE)-observed>0~round(exp(log(expected)-qnorm(0.975)*expected_SE)-observed),
      TRUE~0),
    missedUpp=case_when(
      exp(log(expected)+qnorm(0.975)*expected_SE)-observed>0~round(exp(log(expected)+qnorm(0.975)*expected_SE)-observed),
      TRUE~0)
  ) %>%
  mutate(
    missed_95CI=paste(sep="",format(big.mark=",",round(missed))," (",format(big.mark=",",missedLow),", ",format(big.mark=",",missedUpp),")")
  ) %>%
  mutate(
    observed=format(big.mark=",",observed,scientific=FALSE),
  )
refDat %>%
  dplyr::select(c(iso3,group,observed,expected,expected_SE,missed_95CI)) %>%
  knitr::kable(col.names=c("Country code","Group","Observed","Expected","SE for log(expected)","Missed (95% CI)"),caption = "Observed and expected counts for 2020 for different groups.\nExpected counts are derived from a Poisson regression model fitted to data from 2013-2019.\nThe 95% CIs for missed cases are derived using a normal approximation of the log(count) scale.") %>%
  kableExtra::kable_styling(full_width = FALSE)
```

Observed and expected counts for 2020 for different groups. Expected
counts are derived from a Poisson regression model fitted to data from
2013-2019. The 95% CIs for missed cases are derived using a normal
approximation of the log(count) scale.

| Country code | Group | Observed | Expected | SE for log(expected) | Missed (95% CI) |
| --- | --- | --- | --- | --- | --- |
| AZE | men | 1,610 | 2.094623e+03 | 0.0172395 | 485 ( 415, 557) |
| AZE | women | 857 | 1.183990e+03 | 0.0246906 | 327 ( 271, 386) |
| AZE | children | 91 | 1.736833e+02 | 0.0629607 | 83 ( 63, 105) |
| AZE | adults | 2,282 | 2.999676e+03 | 0.0146360 | 718 ( 633, 805) |
| AZE | elderly | 185 | 2.744279e+02 | 0.0544826 | 89 ( 62, 120) |
| BGD | men | 123,451 | 1.711816e+05 | 0.0027237 | 47,731 ( 46,819, 48,647) |
| BGD | women | 97,264 | 1.326024e+05 | 0.0031719 | 35,338 ( 34,517, 36,165) |
| BGD | children | 9,363 | 1.380906e+04 | 0.0098452 | 4,446 ( 4,182, 4,715) |
| BGD | adults | 181,071 | 2.457084e+05 | 0.0022752 | 64,637 ( 63,544, 65,736) |
| BGD | elderly | 39,644 | 5.843973e+04 | 0.0049395 | 18,796 ( 18,233, 19,364) |
| BLR | men | 1,121 | 1.411039e+03 | 0.0194163 | 290 ( 237, 345) |
| BLR | women | 389 | 5.231910e+02 | 0.0317820 | 134 ( 103, 168) |
| BLR | children | 4 | 8.900447e+00 | 0.2454425 | 5 ( 2, 10) |
| BLR | adults | 1,310 | 1.617207e+03 | 0.0179108 | 307 ( 251, 365) |
| BLR | elderly | 200 | 3.247023e+02 | 0.0436315 | 125 ( 98, 154) |
| BWA | men | 1,258 | 1.544588e+03 | 0.0185022 | 287 ( 232, 344) |
| BWA | women | 811 | 1.055215e+03 | 0.0220943 | 244 ( 199, 291) |
| BWA | children | 160 | 1.798555e+02 | 0.0526858 | 20 ( 2, 39) |
| BWA | adults | 1,845 | 2.300011e+03 | 0.0149573 | 455 ( 389, 523) |
| BWA | elderly | 224 | 3.071108e+02 | 0.0447302 | 83 ( 57, 111) |
| BRA | men | 49,967 | 5.881094e+04 | 0.0035957 | 8,844 ( 8,431, 9,260) |
| BRA | women | 21,522 | 2.418771e+04 | 0.0054669 | 2,666 ( 2,408, 2,926) |
| BRA | children | 1,944 | 2.553194e+03 | 0.0171356 | 609 ( 525, 696) |
| BRA | adults | 64,037 | 7.418952e+04 | 0.0031709 | 10,153 ( 9,693, 10,615) |
| BRA | elderly | 7,452 | 8.772493e+03 | 0.0093849 | 1,320 ( 1,161, 1,483) |
| CMR | men | 13,194 | 1.380293e+04 | 0.0103798 | 609 ( 331, 893) |
| CMR | women | 7,750 | 8.342505e+03 | 0.0129984 | 593 ( 383, 808) |
| CMR | children | 1,158 | 1.143786e+03 | 0.0347823 | 0 ( 0, 66) |
| CMR | adults | 19,432 | 2.051330e+04 | 0.0083944 | 1,081 ( 747, 1,422) |
| CMR | elderly | 1,512 | 1.634602e+03 | 0.0314856 | 123 ( 25, 227) |
| CAF | men | 6,263 | 5.808826e+03 | 0.0165235 | 0 ( 0, 0) |
| CAF | women | 4,643 | 4.635096e+03 | 0.0190730 | 0 ( 0, 169) |
| CAF | children | 1,629 | 1.884625e+03 | 0.0296495 | 256 ( 149, 368) |
| CAF | adults | 10,419 | 9.999406e+03 | 0.0127496 | 0 ( 0, 0) |
| CAF | elderly | 487 | 4.363615e+02 | 0.0619959 | 0 ( 0, 6) |
| CHN | men | 423,296 | 4.993148e+05 | 0.0011657 | 76,019 ( 74,879, 77,161) |
| CHN | women | 194,405 | 2.258724e+05 | 0.0017419 | 31,467 ( 30,698, 32,240) |
| CHN | children | 7,014 | 6.864740e+03 | 0.0111519 | 0 ( 0, 2) |
| CHN | adults | 458,615 | 5.340186e+05 | 0.0011134 | 75,404 ( 74,240, 76,570) |
| CHN | elderly | 159,086 | 1.931768e+05 | 0.0019664 | 34,091 ( 33,348, 34,837) |
| COG | men | 5,902 | 6.632729e+03 | 0.0138319 | 731 ( 553, 913) |
| COG | women | 4,376 | 4.843623e+03 | 0.0159477 | 468 ( 319, 621) |
| COG | children | 897 | 9.887887e+02 | 0.0340352 | 92 ( 28, 160) |
| COG | adults | 9,419 | 1.063146e+04 | 0.0108438 | 1,212 ( 989, 1,441) |
| COG | elderly | 859 | 8.431983e+02 | 0.0390854 | 0 ( 0, 51) |
| PRK | men | 54,238 | 5.715700e+04 | 0.0063587 | 2,919 ( 2,211, 3,636) |
| PRK | women | 30,786 | 2.929159e+04 | 0.0085927 | 0 ( 0, 0) |
| PRK | children | 4,616 | 4.306635e+03 | 0.0222982 | 0 ( 0, 0) |
| PRK | adults | 81,199 | 8.290573e+04 | 0.0052296 | 1,707 ( 861, 2,561) |
| PRK | elderly | 3,825 | 3.503449e+03 | 0.0241663 | 0 ( 0, 0) |
| COD | men | 101,798 | 1.138334e+05 | 0.0078378 | 12,035 ( 10,300, 13,798) |
| COD | women | 76,418 | 8.953481e+04 | 0.0089360 | 13,117 ( 11,562, 14,699) |
| COD | children | 22,340 | 2.418177e+04 | 0.0167087 | 1,842 ( 1,063, 2,647) |
| COD | adults | 165,082 | 1.877800e+05 | 0.0061028 | 22,698 ( 20,465, 24,958) |
| COD | elderly | 13,134 | 1.573477e+04 | 0.0226466 | 2,601 ( 1,918, 3,315) |
| SWZ | men | 1,258 | 1.271147e+03 | 0.0201001 | 13 ( 0, 64) |
| SWZ | women | 783 | 8.747304e+02 | 0.0235601 | 92 ( 52, 133) |
| SWZ | children | 99 | 9.016542e+01 | 0.0624948 | 0 ( 0, 3) |
| SWZ | adults | 1,965 | 2.032903e+03 | 0.0157059 | 68 ( 6, 131) |
| SWZ | elderly | 76 | 1.112449e+02 | 0.0670003 | 35 ( 22, 51) |
| ETH | men | 54,956 | 5.326178e+04 | 0.0043896 | 0 ( 0, 0) |
| ETH | women | 42,398 | 4.122719e+04 | 0.0049614 | 0 ( 0, 0) |
| ETH | children | 10,839 | 9.252340e+03 | 0.0096180 | 0 ( 0, 0) |
| ETH | adults | 92,146 | 8.935973e+04 | 0.0033780 | 0 ( 0, 0) |
| ETH | elderly | 5,208 | 5.130315e+03 | 0.0143060 | 0 ( 0, 68) |
| GAB | men | 2,861 | 3.299376e+03 | 0.0172123 | 438 ( 329, 552) |
| GAB | women | 1,777 | 1.902504e+03 | 0.0219699 | 126 ( 45, 209) |
| GAB | children | 241 | 2.972643e+02 | 0.0526682 | 56 ( 27, 89) |
| GAB | adults | 4,458 | 4.976865e+03 | 0.0139323 | 519 ( 385, 657) |
| GAB | elderly | 180 | 2.330058e+02 | 0.0582165 | 53 ( 28, 81) |
| GIN | men | 9,066 | 9.513430e+03 | 0.0093273 | 447 ( 275, 623) |
| GIN | women | 5,601 | 5.686651e+03 | 0.0123305 | 86 ( 0, 225) |
| GIN | children | 945 | 1.740626e+03 | 0.0300024 | 796 ( 696, 901) |
| GIN | adults | 13,632 | 1.416088e+04 | 0.0076689 | 529 ( 318, 743) |
| GIN | elderly | 1,035 | 1.055958e+03 | 0.0306188 | 21 ( 0, 86) |
| GNB | men | 1,518 | 1.339220e+03 | 0.0258302 | 0 ( 0, 0) |
| GNB | women | 903 | 7.629394e+02 | 0.0339156 | 0 ( 0, 0) |
| GNB | children | 122 | 1.335703e+02 | 0.0860798 | 12 ( 0, 36) |
| GNB | adults | 2,340 | 2.005771e+03 | 0.0210225 | 0 ( 0, 0) |
| GNB | elderly | 81 | 9.631759e+01 | 0.0973740 | 15 ( 0, 36) |
| IND | men | 949,552 | 1.259029e+06 | 0.0008653 | 309,477 (307,344, 311,614) |
| IND | women | 582,210 | 7.527243e+05 | 0.0011637 | 170,514 (168,799, 172,233) |
| IND | children | 97,539 | 1.479933e+05 | 0.0026429 | 50,454 ( 49,690, 51,223) |
| IND | adults | 1,383,074 | 1.808400e+06 | 0.0007292 | 425,326 (422,743, 427,912) |
| IND | elderly | 148,688 | 2.016576e+05 | 0.0022737 | 52,970 ( 52,073, 53,870) |
| IDN | men | 203,415 | 3.167923e+05 | 0.0016871 | 113,377 (112,332, 114,427) |
| IDN | women | 145,149 | 2.284806e+05 | 0.0020012 | 83,332 ( 82,437, 84,230) |
| IDN | children | 35,461 | 9.228103e+04 | 0.0035586 | 56,820 ( 56,179, 57,466) |
| IDN | adults | 313,183 | 4.774452e+05 | 0.0013666 | 164,262 (162,985, 165,543) |
| IDN | elderly | 35,381 | 6.939136e+04 | 0.0039103 | 34,010 ( 33,481, 34,544) |
| KAZ | men | 4,756 | 6.296054e+03 | 0.0097862 | 1,540 ( 1,420, 1,662) |
| KAZ | women | 4,544 | 4.095907e+03 | 0.0122743 | 0 ( 0, 0) |
| KAZ | children | 303 | 3.147484e+02 | 0.0443939 | 12 ( 0, 40) |
| KAZ | adults | 8,155 | 9.039622e+03 | 0.0080943 | 885 ( 742, 1,029) |
| KAZ | elderly | 1,145 | 1.439295e+03 | 0.0234890 | 294 ( 230, 362) |
| KEN | men | 44,034 | 5.198801e+04 | 0.0042088 | 7,954 ( 7,527, 8,385) |
| KEN | women | 22,006 | 2.593054e+04 | 0.0057297 | 3,925 ( 3,635, 4,217) |
| KEN | children | 5,606 | 8.823673e+03 | 0.0103206 | 3,218 ( 3,041, 3,398) |
| KEN | adults | 61,285 | 7.165013e+04 | 0.0035146 | 10,365 ( 9,873, 10,860) |
| KEN | elderly | 4,755 | 6.315776e+03 | 0.0129631 | 1,561 ( 1,402, 1,723) |
| KGZ | men | 2,370 | 3.329786e+03 | 0.0176660 | 960 ( 846, 1,077) |
| KGZ | women | 1,691 | 2.362791e+03 | 0.0209325 | 672 ( 577, 771) |
| KGZ | children | 180 | 2.555622e+02 | 0.0571887 | 76 ( 48, 106) |
| KGZ | adults | 3,480 | 4.826152e+03 | 0.0144432 | 1,346 ( 1,211, 1,485) |
| KGZ | elderly | 581 | 9.104231e+02 | 0.0380709 | 329 ( 264, 400) |
| LSO | men | 2,930 | 4.191290e+03 | 0.0141461 | 1,261 ( 1,147, 1,379) |
| LSO | women | 1,444 | 2.055445e+03 | 0.0189770 | 611 ( 536, 689) |
| LSO | children | 191 | 2.764497e+02 | 0.0544529 | 85 ( 57, 117) |
| LSO | adults | 3,607 | 5.135986e+03 | 0.0122044 | 1,529 ( 1,408, 1,653) |
| LSO | elderly | 767 | 1.207814e+03 | 0.0308074 | 441 ( 370, 516) |
| LBR | men | 3,550 | 4.485654e+03 | 0.0147552 | 936 ( 808, 1,067) |
| LBR | women | 2,349 | 3.526893e+03 | 0.0177595 | 1,178 ( 1,057, 1,303) |
| LBR | children | 1,056 | 2.145140e+03 | 0.0279176 | 1,089 ( 975, 1,210) |
| LBR | adults | 5,569 | 7.423093e+03 | 0.0116834 | 1,854 ( 1,686, 2,026) |
| LBR | elderly | 330 | 5.878027e+02 | 0.0477768 | 258 ( 205, 316) |
| MWI | men | 8,468 | 9.423797e+03 | 0.0088796 | 956 ( 793, 1,121) |
| MWI | women | 5,270 | 5.358469e+03 | 0.0113481 | 88 ( 0, 209) |
| MWI | children | 1,395 | 1.383485e+03 | 0.0217523 | 0 ( 0, 49) |
| MWI | adults | 12,316 | 1.318764e+04 | 0.0073410 | 872 ( 683, 1,063) |
| MWI | elderly | 1,422 | 1.619457e+03 | 0.0230044 | 197 ( 126, 272) |
| MNG | men | 1,908 | 2.097573e+03 | 0.0181136 | 190 ( 116, 265) |
| MNG | women | 1,498 | 1.579016e+03 | 0.0208283 | 81 ( 18, 147) |
| MNG | children | 455 | 3.964652e+02 | 0.0422472 | 0 ( 0, 0) |
| MNG | adults | 3,198 | 3.445687e+03 | 0.0140793 | 248 ( 154, 344) |
| MNG | elderly | 208 | 2.330316e+02 | 0.0569696 | 25 ( 0, 53) |
| MMR | men | 58,384 | 7.290714e+04 | 0.0035211 | 14,523 ( 14,022, 15,028) |
| MMR | women | 31,724 | 3.960999e+04 | 0.0047454 | 7,886 ( 7,519, 8,256) |
| MMR | children | 13,244 | 2.205577e+04 | 0.0056995 | 8,812 ( 8,567, 9,060) |
| MMR | adults | 77,017 | 9.481237e+04 | 0.0030585 | 17,795 ( 17,229, 18,365) |
| MMR | elderly | 13,091 | 1.784764e+04 | 0.0074218 | 4,757 ( 4,499, 5,018) |
| NAM | men | 3,684 | 4.325114e+03 | 0.0127932 | 641 ( 534, 751) |
| NAM | women | 2,181 | 2.602002e+03 | 0.0159895 | 421 ( 341, 504) |
| NAM | children | 644 | 6.467256e+02 | 0.0308540 | 3 ( 0, 43) |
| NAM | adults | 5,435 | 6.409004e+03 | 0.0103592 | 974 ( 845, 1,105) |
| NAM | elderly | 430 | 5.137214e+02 | 0.0376852 | 84 ( 47, 123) |
| NPL | men | 15,950 | 1.923849e+04 | 0.0076205 | 3,288 ( 3,003, 3,578) |
| NPL | women | 9,221 | 1.097487e+04 | 0.0101575 | 1,754 ( 1,538, 1,975) |
| NPL | children | 1,619 | 1.543589e+03 | 0.0254228 | 0 ( 0, 3) |
| NPL | adults | 20,567 | 2.414371e+04 | 0.0067028 | 3,577 ( 3,262, 3,896) |
| NPL | elderly | 4,604 | 6.264822e+03 | 0.0146792 | 1,661 ( 1,483, 1,844) |
| NGA | men | 77,890 | 6.617840e+04 | 0.0034193 | 0 ( 0, 0) |
| NGA | women | 49,417 | 3.786218e+04 | 0.0044157 | 0 ( 0, 0) |
| NGA | children | 8,349 | 9.764717e+03 | 0.0096624 | 1,416 ( 1,233, 1,602) |
| NGA | adults | 116,084 | 9.526475e+04 | 0.0028162 | 0 ( 0, 0) |
| NGA | elderly | 11,223 | 8.767823e+03 | 0.0096542 | 0 ( 0, 0) |
| PAK | men | 123,720 | 1.623103e+05 | 0.0023520 | 38,590 ( 37,844, 39,340) |
| PAK | women | 112,219 | 1.445519e+05 | 0.0024425 | 32,333 ( 31,643, 33,027) |
| PAK | children | 37,051 | 5.542290e+04 | 0.0043785 | 18,372 ( 17,898, 18,850) |
| PAK | adults | 206,818 | 2.643090e+05 | 0.0018112 | 57,491 ( 56,554, 58,431) |
| PAK | elderly | 29,121 | 4.300061e+04 | 0.0047940 | 13,880 ( 13,477, 14,286) |
| PER | men | 14,605 | 2.032882e+04 | 0.0076296 | 5,724 ( 5,422, 6,030) |
| PER | women | 7,952 | 1.034516e+04 | 0.0101663 | 2,393 ( 2,189, 2,601) |
| PER | children | 933 | 1.285927e+03 | 0.0280387 | 353 ( 284, 426) |
| PER | adults | 19,889 | 2.635446e+04 | 0.0065497 | 6,465 ( 6,129, 6,806) |
| PER | elderly | 2,668 | 4.262416e+03 | 0.0167902 | 1,594 ( 1,456, 1,737) |
| PHL | men | 161,117 | 3.321809e+05 | 0.0020169 | 171,064 (169,753, 172,380) |
| PHL | women | 76,964 | 1.757240e+05 | 0.0028031 | 98,760 ( 97,797, 99,728) |
| PHL | children | 18,449 | 5.878428e+04 | 0.0044615 | 40,335 ( 39,823, 40,852) |
| PHL | adults | 199,643 | 4.167898e+05 | 0.0017921 | 217,147 (215,685, 218,613) |
| PHL | elderly | 38,438 | 9.179344e+04 | 0.0040271 | 53,355 ( 52,634, 54,083) |
| MDA | men | 1,309 | 1.876072e+03 | 0.0178811 | 567 ( 502, 634) |
| MDA | women | 402 | 6.473639e+02 | 0.0299452 | 245 ( 208, 284) |
| MDA | children | 56 | 9.479034e+01 | 0.0826172 | 39 ( 25, 55) |
| MDA | adults | 1,567 | 2.286511e+03 | 0.0160108 | 720 ( 649, 792) |
| MDA | elderly | 144 | 2.440000e+02 | 0.0541054 | 100 ( 75, 127) |
| RUS | men | 39,917 | 4.670836e+04 | 0.0039962 | 6,791 ( 6,427, 7,159) |
| RUS | women | 16,774 | 1.970004e+04 | 0.0061457 | 2,926 ( 2,690, 3,165) |
| RUS | children | 1,627 | 1.853144e+03 | 0.0194518 | 226 ( 157, 298) |
| RUS | adults | 52,191 | 6.105502e+04 | 0.0034809 | 8,864 ( 8,449, 9,282) |
| RUS | elderly | 4,500 | 5.407119e+03 | 0.0123473 | 907 ( 778, 1,040) |
| SLE | men | 8,668 | 1.116366e+04 | 0.0098937 | 2,496 ( 2,281, 2,714) |
| SLE | women | 5,489 | 7.296339e+03 | 0.0124184 | 1,807 ( 1,632, 1,987) |
| SLE | children | 1,547 | 4.774999e+03 | 0.0228029 | 3,228 ( 3,019, 3,446) |
| SLE | adults | 13,198 | 1.699901e+04 | 0.0080251 | 3,801 ( 3,536, 4,071) |
| SLE | elderly | 959 | 1.493508e+03 | 0.0293400 | 535 ( 451, 623) |
| SOM | men | 7,802 | 8.510788e+03 | 0.0106559 | 709 ( 533, 888) |
| SOM | women | 5,709 | 5.922351e+03 | 0.0130897 | 213 ( 63, 367) |
| SOM | children | 3,377 | 4.027306e+03 | 0.0154339 | 650 ( 530, 774) |
| SOM | adults | 12,236 | 1.322345e+04 | 0.0086802 | 987 ( 764, 1,214) |
| SOM | elderly | 1,275 | 1.217600e+03 | 0.0270121 | 0 ( 0, 9) |
| ZAF | men | 106,181 | 1.081531e+05 | 0.0023996 | 1,972 ( 1,465, 2,482) |
| ZAF | women | 71,335 | 6.958953e+04 | 0.0029081 | 0 ( 0, 0) |
| ZAF | children | 13,558 | 1.223333e+04 | 0.0062916 | 0 ( 0, 0) |
| ZAF | adults | 173,830 | 1.665420e+05 | 0.0019046 | 0 ( 0, 0) |
| ZAF | elderly | 3,686 | 1.123873e+04 | 0.0078505 | 7,553 ( 7,381, 7,727) |
| TJK | men | 2,068 | 2.993156e+03 | 0.0169701 | 925 ( 827, 1,026) |
| TJK | women | 1,840 | 2.369409e+03 | 0.0189024 | 529 ( 443, 619) |
| TJK | children | 240 | 3.972762e+02 | 0.0481979 | 157 ( 121, 197) |
| TJK | adults | 3,501 | 4.820943e+03 | 0.0132650 | 1,320 ( 1,196, 1,447) |
| TJK | elderly | 407 | 5.447433e+02 | 0.0412416 | 138 ( 95, 184) |
| THA | men | 56,707 | 7.657472e+04 | 0.0044645 | 19,868 ( 19,201, 20,541) |
| THA | women | 27,124 | 3.683011e+04 | 0.0065290 | 9,706 ( 9,238, 10,180) |
| THA | children | 885 | 1.301081e+03 | 0.0362683 | 416 ( 327, 512) |
| THA | adults | 63,197 | 7.087173e+04 | 0.0048796 | 7,675 ( 7,000, 8,356) |
| THA | elderly | 20,634 | 2.207572e+04 | 0.0088024 | 1,442 ( 1,064, 1,826) |
| UKR | men | 12,150 | 1.665364e+04 | 0.0061750 | 4,504 ( 4,303, 4,706) |
| UKR | women | 5,001 | 6.676267e+03 | 0.0097102 | 1,675 ( 1,549, 1,804) |
| UKR | children | 382 | 5.807195e+02 | 0.0350199 | 199 ( 160, 240) |
| UKR | adults | 15,747 | 2.132386e+04 | 0.0054430 | 5,577 ( 5,351, 5,806) |
| UKR | elderly | 1,404 | 2.007583e+03 | 0.0180257 | 604 ( 534, 676) |
| TZA | men | 43,439 | 4.375904e+04 | 0.0046897 | 320 ( 0, 724) |
| TZA | women | 27,762 | 2.680482e+04 | 0.0059494 | 0 ( 0, 0) |
| TZA | children | 13,590 | 1.404378e+04 | 0.0092849 | 454 ( 201, 712) |
| TZA | adults | 59,574 | 5.960872e+04 | 0.0039597 | 35 ( 0, 499) |
| TZA | elderly | 11,627 | 1.125607e+04 | 0.0100406 | 0 ( 0, 0) |
| UZB | men | 5,696 | 6.860579e+03 | 0.0094957 | 1,165 ( 1,038, 1,293) |
| UZB | women | 4,682 | 6.054336e+03 | 0.0106075 | 1,372 ( 1,248, 1,500) |
| UZB | children | 1,733 | 2.148047e+03 | 0.0186787 | 415 ( 338, 495) |
| UZB | adults | 8,591 | 1.062601e+04 | 0.0077056 | 2,035 ( 1,876, 2,197) |
| UZB | elderly | 1,787 | 2.297659e+03 | 0.0178581 | 511 ( 432, 593) |
| VNM | men | 70,754 | 6.961222e+04 | 0.0045611 | 0 ( 0, 0) |
| VNM | women | 27,651 | 3.019598e+04 | 0.0073614 | 2,545 ( 2,112, 2,984) |
| VNM | children | 1,396 | 1.686507e+03 | 0.0297922 | 291 ( 195, 392) |
| VNM | adults | 79,404 | 8.041878e+04 | 0.0042883 | 1,015 ( 342, 1,694) |
| VNM | elderly | 19,001 | 1.917781e+04 | 0.0090702 | 177 ( 0, 521) |
| ZMB | men | 24,602 | 2.222559e+04 | 0.0056880 | 0 ( 0, 0) |
| ZMB | women | 12,674 | 1.058329e+04 | 0.0078027 | 0 ( 0, 0) |
| ZMB | children | 2,724 | 2.019964e+03 | 0.0177626 | 0 ( 0, 0) |
| ZMB | adults | 34,026 | 3.081047e+04 | 0.0047241 | 0 ( 0, 0) |
| ZMB | elderly | 3,250 | 1.922012e+03 | 0.0198828 | 0 ( 0, 0) |
| ZWE | men | 9,622 | 1.289156e+04 | 0.0071247 | 3,270 ( 3,091, 3,451) |
| ZWE | women | 5,194 | 7.116118e+03 | 0.0090178 | 1,922 ( 1,797, 2,049) |
| ZWE | children | 912 | 1.024204e+03 | 0.0225822 | 112 ( 68, 159) |
| ZWE | adults | 13,690 | 1.816952e+04 | 0.0058249 | 4,480 ( 4,273, 4,688) |
| ZWE | elderly | 1,126 | 1.793349e+03 | 0.0198993 | 667 ( 599, 739) |

```
# Total expected cases
#sum(refDat[refDat$group=="men",6])
#sum(refDat[refDat$group=="men",7])
#sum(refDat[refDat$group=="men",8])
#sum(refDat[refDat$group=="men",6])/sum(refDat[refDat$group=="men",4])
#sum(refDat[refDat$group=="women",6])
#sum(refDat[refDat$group=="women",7])
#sum(refDat[refDat$group=="women",8])
#sum(refDat[refDat$group=="women",6])/sum(refDat[refDat$group=="women",4])
#sum(refDat[refDat$group=="children",6])
#sum(refDat[refDat$group=="children",7])
#sum(refDat[refDat$group=="children",8])
#sum(refDat[refDat$group=="children",6])/sum(refDat[refDat$group=="children",4])
#sum(refDat[refDat$group=="adults",6])
#sum(refDat[refDat$group=="adults",7])
#sum(refDat[refDat$group=="adults",8])
#sum(refDat[refDat$group=="adults",6])/sum(refDat[refDat$group=="adults",4])
#sum(refDat[refDat$group=="elderly",6])
#sum(refDat[refDat$group=="elderly",7])
#sum(refDat[refDat$group=="elderly",8])
#sum(refDat[refDat$group=="elderly",6])/sum(refDat[refDat$group=="elderly",4])
```

# 8. Sex risk ratios

We calculate the risk ratios for women compared to men and conduct a
meta analysis on these

```
resm<-analysisFun(refDat=datLong %>% filter(group=="men"),compDat=datLong %>% filter(group!="men"))
# Show data
resm$compDat %>%
  dplyr::select(c(iso3,group,contains("RR"))) %>%
  knitr::kable(col.names=c("Country code","Group","RR","RR (95% CI lower bound)","RR (95% CI upper bound)","SE of log(RR)"),caption = "Relative risks of being missed using men as reference. 10,000 parametric bootstrap samples for the CI and SE.") %>%
  kableExtra::kable_styling(full_width = FALSE)
```

Relative risks of being missed using men as reference. 10,000 parametric
bootstrap samples for the CI and SE.

| Country code | Group | RR | RR (95% CI lower bound) | RR (95% CI upper bound) | SE of log(RR) |
| --- | --- | --- | --- | --- | --- |
| AZE | women | 1.193682e+00 | 1.001955e+00 | 1.408608e+00 | 0.0870072 |
| AZE | children | 2.057603e+00 | 1.705694e+00 | 2.443084e+00 | 0.0913656 |
| AZE | adults | 1.034084e+00 | 8.975691e-01 | 1.200304e+00 | 0.0739524 |
| AZE | elderly | 1.408466e+00 | 1.060039e+00 | 1.769766e+00 | 0.1304519 |
| BGD | women | 9.557755e-01 | 9.352435e-01 | 9.766346e-01 | 0.0110833 |
| BGD | children | 1.154706e+00 | 1.104512e+00 | 1.205207e+00 | 0.0220094 |
| BGD | adults | 9.434610e-01 | 9.262861e-01 | 9.612703e-01 | 0.0094257 |
| BGD | elderly | 1.153483e+00 | 1.126038e+00 | 1.182149e+00 | 0.0125272 |
| BLR | women | 1.247801e+00 | 9.748168e-01 | 1.561338e+00 | 0.1191922 |
| BLR | children | 2.678588e+00 | 1.306514e+00 | 3.682142e+00 | 0.2809714 |
| BLR | adults | 9.241615e-01 | 7.500054e-01 | 1.139925e+00 | 0.1070713 |
| BLR | elderly | 1.868406e+00 | 1.521454e+00 | 2.278709e+00 | 0.1035250 |
| BWA | women | 1.247345e+00 | 9.991516e-01 | 1.557783e+00 | 0.1110844 |
| BWA | children | 5.949934e-01 | 6.772710e-02 | 1.094420e+00 | 0.7088888 |
| BWA | adults | 1.066221e+00 | 8.755808e-01 | 1.307831e+00 | 0.1019580 |
| BWA | elderly | 1.458536e+00 | 1.054672e+00 | 1.909480e+00 | 0.1497480 |
| BRA | women | 7.328754e-01 | 6.628154e-01 | 8.025268e-01 | 0.0488382 |
| BRA | children | 1.586660e+00 | 1.405642e+00 | 1.765083e+00 | 0.0584694 |
| BRA | adults | 9.100046e-01 | 8.606409e-01 | 9.631436e-01 | 0.0286872 |
| BRA | elderly | 1.000980e+00 | 8.900398e-01 | 1.113609e+00 | 0.0577789 |
| CMR | women | 1.609899e+00 | 9.262728e-01 | 3.048622e+00 | 0.3108608 |
| CMR | children | 9.909000e-03 | 7.187900e-03 | 1.299841e+00 | 1.8398693 |
| CMR | adults | 1.194854e+00 | 7.165704e-01 | 2.218753e+00 | 0.2942349 |
| CMR | elderly | 1.700161e+00 | 3.352149e-01 | 3.785752e+00 | 0.7399166 |
| CAF | women |  | 7.488586e+00 | 4.955032e+02 | 1.0942112 |
| CAF | children | 1.575784e+03 | 9.821770e+02 | 2.130498e+03 | 0.2000206 |
| CAF | adults |  | 1.867809e+00 | 8.895899e+01 | 3.6149436 |
| CAF | elderly |  | 1.214052e+01 | 9.099386e+02 | 1.1323784 |
| CHN | women | 9.150638e-01 | 8.928570e-01 | 9.377251e-01 | 0.0125140 |
| CHN | children | 4.784000e-04 | 4.680000e-04 | 2.195400e-03 | 0.6200523 |
| CHN | adults | 9.274472e-01 | 9.106181e-01 | 9.449529e-01 | 0.0094298 |
| CHN | elderly | 1.159140e+00 | 1.133681e+00 | 1.184667e+00 | 0.0112423 |
| COG | women | 8.763173e-01 | 5.903512e-01 | 1.257231e+00 | 0.1917196 |
| COG | children | 8.426006e-01 | 2.731990e-01 | 1.462911e+00 | 0.4958565 |
| COG | adults | 1.035168e+00 | 7.918574e-01 | 1.377976e+00 | 0.1403882 |
| COG | elderly | 5.382400e-03 | 4.515200e-03 | 5.261971e-01 | 1.6440386 |
| PRK | women | 3.342000e-04 | 2.706000e-04 | 4.344000e-04 | 0.1204795 |
| PRK | children | 2.273400e-03 | 1.838900e-03 | 2.976500e-03 | 0.1671605 |
| PRK | adults | 4.031026e-01 | 1.978464e-01 | 6.502549e-01 | 0.3056966 |
| PRK | elderly | 2.794500e-03 | 2.257200e-03 | 3.665900e-03 | 0.1504137 |
| COD | women | 1.385626e+00 | 1.181318e+00 | 1.645328e+00 | 0.0854904 |
| COD | children | 7.203739e-01 | 4.250734e-01 | 1.023613e+00 | 0.2256163 |
| COD | adults | 1.143269e+00 | 9.837396e-01 | 1.349116e+00 | 0.0808495 |
| COD | elderly | 1.563331e+00 | 1.177949e+00 | 1.996138e+00 | 0.1351917 |
| SWZ | women | 1.013923e+01 | 1.888278e+00 | 3.415949e+02 | 1.8767335 |
| SWZ | children | 5.361616e-01 | 1.029050e-01 | 3.284567e+01 | 1.3513578 |
| SWZ | adults | 3.229532e+00 | 2.578537e-01 | 1.383537e+02 | 2.0375766 |
| SWZ | elderly | 3.063248e+01 | 6.025797e+00 | 9.543311e+02 | 1.8737540 |
| ETH | women |  |  |  |  |
| ETH | children |  |  |  |  |
| ETH | adults |  |  |  |  |
| ETH | elderly |  | 2.603498e+01 | 2.413710e+03 | 1.1905232 |
| GAB | women | 4.964982e-01 | 1.804933e-01 | 8.486829e-01 | 0.4261691 |
| GAB | children | 1.424540e+00 | 7.492687e-01 | 2.185137e+00 | 0.2795860 |
| GAB | adults | 7.846630e-01 | 5.596834e-01 | 1.084601e+00 | 0.1684168 |
| GAB | elderly | 1.712149e+00 | 9.773615e-01 | 2.571470e+00 | 0.2455091 |
| GIN | women | 3.202494e-01 | 1.639800e-03 | 9.422380e-01 | 1.7976663 |
| GIN | children | 9.718855e+00 | 7.059799e+00 | 1.578999e+01 | 0.2059289 |
| GIN | adults | 7.940998e-01 | 4.429750e-01 | 1.406916e+00 | 0.2939224 |
| GIN | elderly | 4.220118e-01 | 8.296200e-03 | 1.807958e+00 | 1.8969709 |
| GNB | women |  |  |  |  |
| GNB | children | 2.320155e+02 | 2.107883e+01 | 6.297608e+02 | 0.8881165 |
| GNB | adults |  |  |  |  |
| GNB | elderly | 4.259581e+02 | 6.309046e+01 | 8.179293e+02 | 0.6956712 |
| IND | women | 9.215774e-01 | 9.129602e-01 | 9.303505e-01 | 0.0047707 |
| IND | children | 1.386956e+00 | 1.371362e+00 | 1.402409e+00 | 0.0057322 |
| IND | adults | 9.568287e-01 | 9.501945e-01 | 9.635319e-01 | 0.0035651 |
| IND | elderly | 1.068609e+00 | 1.054348e+00 | 1.082811e+00 | 0.0068504 |
| IDN | women | 1.019081e+00 | 1.010104e+00 | 1.028330e+00 | 0.0046113 |
| IDN | children | 1.720432e+00 | 1.707679e+00 | 1.733257e+00 | 0.0037611 |
| IDN | adults | 9.613081e-01 | 9.537588e-01 | 9.687264e-01 | 0.0039846 |
| IDN | elderly | 1.369476e+00 | 1.356110e+00 | 1.382707e+00 | 0.0050123 |
| KAZ | women | 4.991000e-04 | 4.691000e-04 | 5.332000e-04 | 0.0326378 |
| KAZ | children | 1.525970e-01 | 6.577900e-03 | 4.867228e-01 | 1.5165506 |
| KAZ | adults | 4.000737e-01 | 3.381065e-01 | 4.653684e-01 | 0.0810427 |
| KAZ | elderly | 8.359210e-01 | 6.789684e-01 | 9.934088e-01 | 0.0977814 |
| KEN | women | 9.892232e-01 | 9.156356e-01 | 1.066625e+00 | 0.0393251 |
| KEN | children | 2.383469e+00 | 2.250461e+00 | 2.525131e+00 | 0.0294159 |
| KEN | adults | 9.455283e-01 | 8.893136e-01 | 1.004247e+00 | 0.0311357 |
| KEN | elderly | 1.615217e+00 | 1.471113e+00 | 1.759753e+00 | 0.0459229 |
| KGZ | women | 9.863944e-01 | 8.602553e-01 | 1.131344e+00 | 0.0693882 |
| KGZ | children | 1.025769e+00 | 7.273809e-01 | 1.312800e+00 | 0.1508088 |
| KGZ | adults | 9.676869e-01 | 8.630849e-01 | 1.086273e+00 | 0.0580410 |
| KGZ | elderly | 1.255314e+00 | 1.055931e+00 | 1.459626e+00 | 0.0823629 |
| LSO | women | 9.885176e-01 | 8.853580e-01 | 1.099347e+00 | 0.0558217 |
| LSO | children | 1.027134e+00 | 7.626470e-01 | 1.271844e+00 | 0.1296853 |
| LSO | adults | 9.892644e-01 | 9.090073e-01 | 1.078137e+00 | 0.0441308 |
| LSO | elderly | 1.212797e+00 | 1.065789e+00 | 1.364899e+00 | 0.0636423 |
| LBR | women | 1.601121e+00 | 1.408553e+00 | 1.831896e+00 | 0.0670985 |
| LBR | children | 2.434102e+00 | 2.161623e+00 | 2.769356e+00 | 0.0630118 |
| LBR | adults | 1.197450e+00 | 1.055306e+00 | 1.370933e+00 | 0.0665573 |
| LBR | elderly | 2.102648e+00 | 1.782815e+00 | 2.475901e+00 | 0.0832979 |
| MWI | women | 1.627827e-01 | 9.061000e-04 | 3.802753e-01 | 1.5231965 |
| MWI | children | 3.563300e-03 | 3.135900e-03 | 3.220863e-01 | 1.6671740 |
| MWI | adults | 6.516734e-01 | 4.959253e-01 | 8.351899e-01 | 0.1323920 |
| MWI | elderly | 1.202165e+00 | 7.779107e-01 | 1.658640e+00 | 0.1893889 |
| MNG | women | 5.677070e-01 | 1.210853e-01 | 1.160483e+00 | 0.6511140 |
| MNG | children | 1.395420e-02 | 1.020330e-02 | 2.233450e-02 | 0.2001713 |
| MNG | adults | 7.953683e-01 | 4.590566e-01 | 1.381772e+00 | 0.2726535 |
| MNG | elderly | 1.188542e+00 | 4.333010e-02 | 2.554623e+00 | 0.8523718 |
| MMR | women | 9.994499e-01 | 9.538812e-01 | 1.046781e+00 | 0.0240347 |
| MMR | children | 2.005629e+00 | 1.941352e+00 | 2.073615e+00 | 0.0166614 |
| MMR | adults | 9.422182e-01 | 9.067895e-01 | 9.791686e-01 | 0.0196746 |
| MMR | elderly | 1.337916e+00 | 1.274480e+00 | 1.403827e+00 | 0.0246966 |
| NAM | women | 1.091537e+00 | 8.769346e-01 | 1.348825e+00 | 0.1104978 |
| NAM | children | 2.843150e-02 | 4.731600e-03 | 4.349893e-01 | 1.7355207 |
| NAM | adults | 1.025256e+00 | 8.562132e-01 | 1.233319e+00 | 0.0930277 |
| NAM | elderly | 1.099439e+00 | 6.680033e-01 | 1.558078e+00 | 0.2172009 |
| NPL | women | 9.349177e-01 | 8.211641e-01 | 1.055746e+00 | 0.0651265 |
| NPL | children | 1.895000e-03 | 1.746500e-03 | 1.651390e-02 | 0.5712275 |
| NPL | adults | 8.666723e-01 | 7.806919e-01 | 9.634449e-01 | 0.0535397 |
| NPL | elderly | 1.550919e+00 | 1.392259e+00 | 1.726821e+00 | 0.0551275 |
| NGA | women |  |  |  |  |
| NGA | children | 1.918948e+04 | 1.697794e+04 | 2.130096e+04 | 0.0578601 |
| NGA | adults |  |  |  |  |
| NGA | elderly |  |  |  |  |
| PAK | women | 9.407801e-01 | 9.203454e-01 | 9.620620e-01 | 0.0113008 |
| PAK | children | 1.394224e+00 | 1.362992e+00 | 1.426032e+00 | 0.0115418 |
| PAK | adults | 9.148624e-01 | 8.967227e-01 | 9.324683e-01 | 0.0098928 |
| PAK | elderly | 1.357595e+00 | 1.324693e+00 | 1.391518e+00 | 0.0125036 |
| PER | women | 8.215999e-01 | 7.585097e-01 | 8.856285e-01 | 0.0393470 |
| PER | children | 9.747544e-01 | 8.234499e-01 | 1.119762e+00 | 0.0781909 |
| PER | adults | 8.713079e-01 | 8.237959e-01 | 9.210173e-01 | 0.0285529 |
| PER | elderly | 1.328533e+00 | 1.237909e+00 | 1.418465e+00 | 0.0345280 |
| PHL | women | 1.091356e+00 | 1.085049e+00 | 1.097388e+00 | 0.0028763 |
| PHL | children | 1.332417e+00 | 1.325085e+00 | 1.339535e+00 | 0.0027841 |
| PHL | adults | 1.011702e+00 | 1.006729e+00 | 1.016641e+00 | 0.0024928 |
| PHL | elderly | 1.128713e+00 | 1.121126e+00 | 1.136260e+00 | 0.0034491 |
| MDA | women | 1.253931e+00 | 1.101588e+00 | 1.417488e+00 | 0.0641176 |
| MDA | children | 1.353851e+00 | 1.002455e+00 | 1.681099e+00 | 0.1326358 |
| MDA | adults | 1.041060e+00 | 9.365804e-01 | 1.158527e+00 | 0.0538946 |
| MDA | elderly | 1.355881e+00 | 1.125435e+00 | 1.591946e+00 | 0.0882860 |
| RUS | women | 1.021528e+00 | 9.395748e-01 | 1.106693e+00 | 0.0417572 |
| RUS | children | 8.392932e-01 | 6.027518e-01 | 1.071001e+00 | 0.1487473 |
| RUS | adults | 9.984977e-01 | 9.403414e-01 | 1.061270e+00 | 0.0310953 |
| RUS | elderly | 1.153814e+00 | 1.006202e+00 | 1.303564e+00 | 0.0669990 |
| SLE | women | 1.108041e+00 | 9.994532e-01 | 1.223483e+00 | 0.0515152 |
| SLE | children | 3.023996e+00 | 2.824332e+00 | 3.259361e+00 | 0.0364038 |
| SLE | adults | 1.000223e+00 | 9.176158e-01 | 1.092928e+00 | 0.0445189 |
| SLE | elderly | 1.600912e+00 | 1.405905e+00 | 1.806659e+00 | 0.0641489 |
| SOM | women | 4.325679e-01 | 1.347415e-01 | 7.731734e-01 | 0.5281230 |
| SOM | children | 1.938906e+00 | 1.483613e+00 | 2.606386e+00 | 0.1435784 |
| SOM | adults | 8.966523e-01 | 6.534684e-01 | 1.233676e+00 | 0.1625686 |
| SOM | elderly | 4.930800e-03 | 4.012900e-03 | 7.749990e-02 | 0.6317912 |
| ZAF | women | 3.940000e-04 | 3.133000e-04 | 5.311000e-04 | 0.1337049 |
| ZAF | children | 2.241400e-03 | 1.782100e-03 | 3.021500e-03 | 0.1338670 |
| ZAF | adults | 1.646000e-04 | 1.309000e-04 | 2.220000e-04 | 0.1336548 |
| ZAF | elderly | 3.685438e+01 | 2.930516e+01 | 4.963667e+01 | 0.1336770 |
| TJK | women | 7.228796e-01 | 6.175481e-01 | 8.317301e-01 | 0.0762151 |
| TJK | children | 1.280811e+00 | 1.067804e+00 | 1.491767e+00 | 0.0853703 |
| TJK | adults | 8.858043e-01 | 7.987998e-01 | 9.807546e-01 | 0.0522577 |
| TJK | elderly | 8.180747e-01 | 6.086520e-01 | 1.025387e+00 | 0.1320042 |
| THA | women | 1.015733e+00 | 9.725716e-01 | 1.060790e+00 | 0.0221437 |
| THA | children | 1.232569e+00 | 1.034194e+00 | 1.413724e+00 | 0.0804505 |
| THA | adults | 4.173760e-01 | 3.826797e-01 | 4.517589e-01 | 0.0425852 |
| THA | elderly | 2.517123e-01 | 1.895550e-01 | 3.146770e-01 | 0.1298036 |
| UKR | women | 9.278888e-01 | 8.676755e-01 | 9.883492e-01 | 0.0330985 |
| UKR | children | 1.265376e+00 | 1.088402e+00 | 1.430384e+00 | 0.0693940 |
| UKR | adults | 9.670957e-01 | 9.254166e-01 | 1.010856e+00 | 0.0225587 |
| UKR | elderly | 1.111755e+00 | 1.013587e+00 | 1.210329e+00 | 0.0452636 |
| TZA | women | 2.550500e-03 | 1.132900e-03 | 2.556650e-02 | 0.8083680 |
| TZA | children | 4.418042e+00 | 1.433025e+00 | 2.881162e+03 | 1.6867019 |
| TZA | adults | 7.963820e-02 | 5.642000e-04 | 1.156770e+02 | 3.3592309 |
| TZA | elderly | 6.073600e-03 | 2.691700e-03 | 6.210980e-02 | 0.8167581 |
| UZB | women | 1.335321e+00 | 1.194121e+00 | 1.505541e+00 | 0.0591016 |
| UZB | children | 1.138270e+00 | 9.439278e-01 | 1.354221e+00 | 0.0922904 |
| UZB | adults | 1.128207e+00 | 1.012837e+00 | 1.269313e+00 | 0.0569501 |
| UZB | elderly | 1.309295e+00 | 1.122108e+00 | 1.521507e+00 | 0.0778157 |
| VNM | women | 1.173411e+04 | 9.874507e+03 | 1.356217e+04 | 0.0801398 |
| VNM | children | 2.398189e+04 | 1.717093e+04 | 3.041928e+04 | 0.1473955 |
| VNM | adults | 1.756832e+03 | 6.135858e+02 | 2.897260e+03 | 0.4115728 |
| VNM | elderly | 1.283614e+03 | 1.172528e+02 | 3.816411e+03 | 0.9069722 |
| ZMB | women |  |  |  |  |
| ZMB | children |  |  |  |  |
| ZMB | adults |  |  |  |  |
| ZMB | elderly |  |  |  |  |
| ZWE | women | 1.065008e+00 | 1.000053e+00 | 1.134312e+00 | 0.0322325 |
| ZWE | children | 4.319531e-01 | 2.740941e-01 | 5.901081e-01 | 0.1950339 |
| ZWE | adults | 9.720844e-01 | 9.218511e-01 | 1.026355e+00 | 0.0274470 |
| ZWE | elderly | 1.467251e+00 | 1.355819e+00 | 1.584467e+00 | 0.0397051 |

```
gm<-plotFun(resm)
# print(gm$ridgePlot)
# Prep data for meta-analysis
resm1<-as.data.frame(resm$compDat)
resm1<-resm1[resm1$group=="women",]
resm1$Region<-dat[year=="2020",g_whoregion]
resm1$Country<-dat[year=="2020",country]
resm1$observed_M<-as.data.frame(resm$reference[,3])
resm1$expected_M<-as.data.frame(resm$reference[,4])
# Remove RR of Na
resm1<-subset(resm1,!is.na(RR))
# Conduct meta-analysis
Risk_sex<-metagen(studlab=Country,sm="RR",TE=log(RR),subgroup=Region,fixed=FALSE,data=resm1,backtransf=TRUE,lower=log(RR_low),upper=log(RR_upp))
# Countries with evidence of effect
MF_evidence_hi_strong<-sum(exp(Risk_sex$TE)>1.1&(Risk_sex$pval<0.01))
MF_evidence_hi_med<-sum(exp(Risk_sex$TE)>1.1&Risk_sex$pval>0.01&Risk_sex$pval<0.05)
MF_evidence_hi_weak<-sum(exp(Risk_sex$TE)>1.25&Risk_sex$pval>0.05&Risk_sex$pval<0.1)
MF_evidence_lo_strong<-sum(exp(Risk_sex$TE)<1/1.1&(Risk_sex$pval<0.01))
MF_evidence_lo_med<-sum(exp(Risk_sex$TE)<1/1.1&Risk_sex$pval>0.01&Risk_sex$pval<0.05)
MF_evidence_lo_weak<-sum(exp(Risk_sex$TE)<1/1.25&Risk_sex$pval>0.05&Risk_sex$pval<0.1)
# P-value for regional subgroups
# Risk_sex$pval.random.w
print("Meta-analysis for women/men")
```

```
## [1] "Meta-analysis for women/men"
```

```
Risk_sex
```

```
## Number of studies combined: k = 40
## 
##                          RR           95%-CI     z p-value
## Random effects model 0.5908 [0.2456; 1.4210] -1.18  0.2399
## 
## Quantifying heterogeneity:
##  tau^2 = 7.8480 [5.1877; 12.8961]; tau = 2.8014 [2.2777; 3.5911]
##  I^2 = 99.9% [99.9%; 100.0%]; H = 44.44 [43.58; 45.31]
## 
## Test of heterogeneity:
##         Q d.f. p-value
##  77013.97   39       0
## 
## Results for subgroups (random effects model):
##                                         k     RR              95%-CI   tau^2
## Region = European Region                9 0.4551 [0.0852;    2.4306]  6.5715
## Region = South-East Asia Region         7 0.3120 [0.0335;    2.9052]  9.0694
## Region = African Region                16 0.4319 [0.1229;    1.5175]  6.1889
## Region = Region of the Americas         2 0.7788 [0.6964;    0.8708]  0.0046
## Region = Western Pacific Region         4 9.1167 [0.0829; 1002.9025] 22.9231
## Region = Eastern Mediterranean Region   2 0.7249 [0.3532;    1.4878]  0.2024
##                                          tau        Q    I^2
## Region = European Region              2.5635 40112.18 100.0%
## Region = South-East Asia Region       3.0115  4596.85  99.9%
## Region = African Region               2.4877  3524.96  99.6%
## Region = Region of the Americas       0.0675     3.31  69.8%
## Region = Western Pacific Region       4.7878 13348.27 100.0%
## Region = Eastern Mediterranean Region 0.4499     3.04  67.1%
## 
## Test for subgroup differences (random effects model):
##                     Q d.f. p-value
## Between groups   2.95    5  0.7079
## 
## Details on meta-analytical method:
## - Inverse variance method
## - Restricted maximum-likelihood estimator for tau^2
## - Q-profile method for confidence interval of tau^2 and tau
```

```
# Sensitivity analysis removing countries with more cases than expected
resm1s<-resm1[resm1$moreObsThanExp=="no"&(resm1$expected_M>resm1$observed_M),]
Risk_sexS<-metagen(studlab=Country,sm="RR",TE=log(RR),subgroup=Region,fixed=FALSE,data=resm1s,backtransf=TRUE,lower=log(RR_low),upper=log(RR_upp))
print("Sensitivity analysis for women/men removing countries with more cases than expected")
```

```
## [1] "Sensitivity analysis for women/men removing countries with more cases than expected"
```

```
Risk_sexS
```

```
## Number of studies combined: k = 35
## 
##                          RR           95%-CI    z p-value
## Random effects model 1.0214 [0.9574; 1.0897] 0.64  0.5210
## 
## Quantifying heterogeneity:
##  tau^2 = 0.0275 [0.0183; 0.0966]; tau = 0.1659 [0.1354; 0.3108]
##  I^2 = 97.5% [97.1%; 97.9%]; H = 6.35 [5.85; 6.89]
## 
## Test of heterogeneity:
##        Q d.f.  p-value
##  1370.64   34 < 0.0001
## 
## Results for subgroups (random effects model):
##                                         k     RR           95%-CI  tau^2    tau
## Region = European Region                8 1.0627 [0.9234; 1.2231] 0.0361 0.1899
## Region = South-East Asia Region         6 0.9766 [0.9404; 1.0142] 0.0018 0.0420
## Region = African Region                14 1.1346 [1.0073; 1.2779] 0.0281 0.1676
## Region = Region of the Americas         2 0.7788 [0.6964; 0.8708] 0.0046 0.0675
## Region = Western Pacific Region         3 0.9875 [0.8329; 1.1709] 0.0154 0.1239
## Region = Eastern Mediterranean Region   2 0.7249 [0.3532; 1.4878] 0.2024 0.4499
##                                            Q   I^2
## Region = European Region               66.01 89.4%
## Region = South-East Asia Region       237.97 97.9%
## Region = African Region                64.11 79.7%
## Region = Region of the Americas         3.31 69.8%
## Region = Western Pacific Region       189.63 98.9%
## Region = Eastern Mediterranean Region   3.04 67.1%
## 
## Test for subgroup differences (random effects model):
##                      Q d.f. p-value
## Between groups   23.88    5  0.0002
## 
## Details on meta-analytical method:
## - Inverse variance method
## - Restricted maximum-likelihood estimator for tau^2
## - Q-profile method for confidence interval of tau^2 and tau
```

```
# Sensitivity analysis removing countries with poor fit or limited data
resm1p<-resm1[!resm1$iso3%in%c("AZE","BRA","CMR","CAF","COD","LBR","SLE","PRK","GAB","GNB","KEN","LSO","MWI","MNG","MMR","NGA","PAK","PER","TJK","UZB","VNM","ZMB"),]
Risk_sexP<-metagen(studlab=Country,sm="RR",TE=log(RR),subgroup=Region,fixed=FALSE,data=resm1p,backtransf=TRUE,lower=log(RR_low),upper=log(RR_upp))
print("Sensitivity analysis for women/men removing countries with a poor fit or limited data")
```

```
## [1] "Sensitivity analysis for women/men removing countries with a poor fit or limited data"
```

```
Risk_sexP
```

```
## Number of studies combined: k = 22
## 
##                          RR           95%-CI     z p-value
## Random effects model 0.3884 [0.1307; 1.1545] -1.70  0.0889
## 
## Quantifying heterogeneity:
##  tau^2 = 6.6012 [3.8327; 13.6897]; tau = 2.5693 [1.9577; 3.7000]
##  I^2 = 100.0%; H = 53.01 [51.80; 54.24]
## 
## Test of heterogeneity:
##         Q d.f. p-value
##  59004.61   21       0
## 
## Results for subgroups (random effects model):
##                                         k     RR           95%-CI   tau^2
## Region = South-East Asia Region         5 0.9722 [0.9299; 1.0164]  0.0021
## Region = European Region                6 0.2999 [0.0243; 3.6944]  9.8438
## Region = African Region                 8 0.2029 [0.0177; 2.3259] 11.8174
## Region = Western Pacific Region         2 0.9998 [0.8412; 1.1882]  0.0154
## Region = Eastern Mediterranean Region   1 0.4326 [0.1806; 1.0362]      --
##                                          tau        Q    I^2
## Region = South-East Asia Region       0.0458   236.54  98.3%
## Region = European Region              3.1375 36962.60 100.0%
## Region = African Region               3.4376  3354.44  99.8%
## Region = Western Pacific Region       0.1242   188.38  99.5%
## Region = Eastern Mediterranean Region     --     0.00     --
## 
## Test for subgroup differences (random effects model):
##                     Q d.f. p-value
## Between groups   5.83    4  0.2124
## 
## Details on meta-analytical method:
## - Inverse variance method
## - Restricted maximum-likelihood estimator for tau^2
## - Q-profile method for confidence interval of tau^2 and tau
```

# 9. Age risk ratios

We calculate the risk ratios for children compared to adults and
elderly compared to adults, and conduct a meta analysis on these

```
resa<-analysisFun(refDat=datLong %>% filter(group=="adults"),compDat=datLong %>% filter(group!="adults"))
# Show data
resa$compDat %>%
  dplyr::select(c(iso3,group,contains("RR"))) %>%
  knitr::kable(col.names=c("Country code","Group","RR","RR (95% CI lower bound)","RR (95% CI upper bound)","SE of log(RR)"),caption = "Relative risks of being missed using adults as reference. 10,000 parametric bootstrap samples for the CI and SE.") %>%
  kableExtra::kable_styling(full_width = FALSE)
```

Relative risks of being missed using adults as reference. 10,000
parametric bootstrap samples for the CI and SE.

| Country code | Group | RR | RR (95% CI lower bound) | RR (95% CI upper bound) | SE of log(RR) |
| --- | --- | --- | --- | --- | --- |
| AZE | men | 9.670396e-01 | 8.335580e-01 | 1.120931e+00 | 0.0750656 |
| AZE | women | 1.154338e+00 | 9.807447e-01 | 1.343334e+00 | 0.0801841 |
| AZE | children | 1.989783e+00 | 1.657591e+00 | 2.325858e+00 | 0.0853989 |
| AZE | elderly | 1.362043e+00 | 1.025529e+00 | 1.681152e+00 | 0.1246770 |
| BGD | men | 1.059927e+00 | 1.040510e+00 | 1.079828e+00 | 0.0094761 |
| BGD | women | 1.013052e+00 | 9.917791e-01 | 1.034772e+00 | 0.0107885 |
| BGD | children | 1.223904e+00 | 1.171877e+00 | 1.275803e+00 | 0.0215547 |
| BGD | elderly | 1.222608e+00 | 1.193556e+00 | 1.251993e+00 | 0.0120883 |
| BLR | men | 1.082062e+00 | 8.779121e-01 | 1.341827e+00 | 0.1073190 |
| BLR | women | 1.350198e+00 | 1.044975e+00 | 1.698142e+00 | 0.1229167 |
| BLR | children | 2.898398e+00 | 1.462525e+00 | 3.979088e+00 | 0.2836551 |
| BLR | elderly | 2.021731e+00 | 1.641547e+00 | 2.483821e+00 | 0.1046487 |
| BWA | men | 9.378920e-01 | 7.614955e-01 | 1.136710e+00 | 0.1030039 |
| BWA | women | 1.169874e+00 | 9.693681e-01 | 1.409736e+00 | 0.0958380 |
| BWA | children | 5.580395e-01 | 5.804150e-02 | 1.017538e+00 | 0.7181688 |
| BWA | elderly | 1.367949e+00 | 1.005567e+00 | 1.742552e+00 | 0.1408893 |
| BRA | men | 1.098896e+00 | 1.039891e+00 | 1.160838e+00 | 0.0279068 |
| BRA | women | 8.053534e-01 | 7.309247e-01 | 8.829873e-01 | 0.0486179 |
| BRA | children | 1.743574e+00 | 1.543135e+00 | 1.937767e+00 | 0.0582623 |
| BRA | elderly | 1.099973e+00 | 9.798241e-01 | 1.221955e+00 | 0.0562379 |
| CMR | men | 8.369225e-01 | 4.381949e-01 | 1.385915e+00 | 0.2913913 |
| CMR | women | 1.347360e+00 | 8.320907e-01 | 2.114714e+00 | 0.2341106 |
| CMR | children | 8.293000e-03 | 6.638400e-03 | 1.054499e+00 | 1.8258801 |
| CMR | elderly | 1.422903e+00 | 3.094842e-01 | 2.749634e+00 | 0.6680740 |
| CAF | men |  | 9.595200e-03 | 7.279761e-01 | 1.9257339 |
| CAF | women |  | 1.268497e+01 | 8.181859e+02 | 1.1181117 |
| CAF | children | 2.712579e+03 | 1.677898e+03 | 3.700503e+03 | 0.2306963 |
| CAF | elderly |  | 7.274097e+00 | 1.421827e+03 | 1.4995680 |
| CHN | men | 1.078229e+00 | 1.058838e+00 | 1.098451e+00 | 0.0093712 |
| CHN | women | 9.866479e-01 | 9.625331e-01 | 1.011289e+00 | 0.0125958 |
| CHN | children | 5.158000e-04 | 5.045000e-04 | 1.048200e-03 | 0.5988659 |
| CHN | elderly | 1.249818e+00 | 1.221533e+00 | 1.278209e+00 | 0.0115452 |
| COG | men | 9.660267e-01 | 7.189437e-01 | 1.265732e+00 | 0.1431177 |
| COG | women | 8.465459e-01 | 5.746739e-01 | 1.153060e+00 | 0.1806671 |
| COG | children | 8.139747e-01 | 2.638435e-01 | 1.385971e+00 | 0.4960815 |
| COG | elderly | 5.199500e-03 | 4.543300e-03 | 4.931879e-01 | 1.6612002 |
| PRK | men | 2.480758e+00 | 1.552197e+00 | 4.953659e+00 | 0.2978724 |
| PRK | women | 8.292000e-04 | 5.570000e-04 | 1.609200e-03 | 0.2734747 |
| PRK | children | 5.639600e-03 | 3.784900e-03 | 1.095670e-02 | 0.2783957 |
| PRK | elderly | 6.932600e-03 | 4.638000e-03 | 1.350250e-02 | 0.2745305 |
| COD | men | 8.746847e-01 | 7.445653e-01 | 1.020798e+00 | 0.0812312 |
| COD | women | 1.211986e+00 | 1.059529e+00 | 1.384901e+00 | 0.0684886 |
| COD | children | 6.301000e-01 | 3.823373e-01 | 8.867338e-01 | 0.2185283 |
| COD | elderly | 1.367422e+00 | 1.037898e+00 | 1.702424e+00 | 0.1259531 |
| SWZ | men | 3.096424e-01 | 7.454100e-03 | 3.894012e+00 | 2.0100504 |
| SWZ | women | 3.139535e+00 | 1.382589e+00 | 3.005383e+01 | 0.8418939 |
| SWZ | children | 1.660183e-01 | 8.735010e-02 | 1.493523e+00 | 0.7413539 |
| SWZ | elderly | 9.485113e+00 | 4.676456e+00 | 9.301572e+01 | 0.8280835 |
| ETH | men |  |  |  |  |
| ETH | women |  |  |  |  |
| ETH | children |  |  |  |  |
| ETH | elderly |  | 3.047626e+01 | 4.132809e+03 | 1.2530387 |
| GAB | men | 1.274433e+00 | 9.111950e-01 | 1.775929e+00 | 0.1683078 |
| GAB | women | 6.327534e-01 | 2.280833e-01 | 1.091246e+00 | 0.4560411 |
| GAB | children | 1.815480e+00 | 9.510292e-01 | 2.840146e+00 | 0.2799027 |
| GAB | elderly | 2.182018e+00 | 1.261462e+00 | 3.345034e+00 | 0.2494564 |
| GIN | men | 1.259288e+00 | 7.199042e-01 | 2.256353e+00 | 0.2936421 |
| GIN | women | 4.032861e-01 | 2.089500e-03 | 1.192335e+00 | 1.7644720 |
| GIN | children | 1.223883e+01 | 8.723926e+00 | 2.036576e+01 | 0.2180688 |
| GIN | elderly | 5.314341e-01 | 1.020960e-02 | 2.318303e+00 | 1.9010092 |
| GNB | men |  |  |  |  |
| GNB | women |  |  |  |  |
| GNB | children | 3.474931e+02 | 2.827332e+01 | 9.276847e+02 | 0.9096955 |
| GNB | elderly | 6.379640e+02 | 9.344468e+01 | 1.222367e+03 | 0.6807544 |
| IND | men | 1.045119e+00 | 1.037848e+00 | 1.052518e+00 | 0.0035559 |
| IND | women | 9.631582e-01 | 9.544110e-01 | 9.720959e-01 | 0.0046619 |
| IND | children | 1.449535e+00 | 1.433491e+00 | 1.465197e+00 | 0.0055723 |
| IND | elderly | 1.116824e+00 | 1.102207e+00 | 1.131984e+00 | 0.0067493 |
| IDN | men | 1.040249e+00 | 1.032163e+00 | 1.048425e+00 | 0.0039641 |
| IDN | women | 1.060099e+00 | 1.051169e+00 | 1.069076e+00 | 0.0043071 |
| IDN | children | 1.789678e+00 | 1.777922e+00 | 1.801433e+00 | 0.0033837 |
| IDN | elderly | 1.424596e+00 | 1.411127e+00 | 1.438136e+00 | 0.0048561 |
| KAZ | men | 2.499539e+00 | 2.152426e+00 | 2.958003e+00 | 0.0808195 |
| KAZ | women | 1.247400e-03 | 1.087600e-03 | 1.462400e-03 | 0.0757314 |
| KAZ | children | 3.814221e-01 | 1.553190e-02 | 1.224367e+00 | 1.5050375 |
| KAZ | elderly | 2.089417e+00 | 1.631628e+00 | 2.617447e+00 | 0.1191640 |
| KEN | men | 1.057610e+00 | 9.941846e-01 | 1.124127e+00 | 0.0315348 |
| KEN | women | 1.046212e+00 | 9.689061e-01 | 1.126316e+00 | 0.0384311 |
| KEN | children | 2.520780e+00 | 2.387248e+00 | 2.659032e+00 | 0.0276715 |
| KEN | elderly | 1.708269e+00 | 1.560234e+00 | 1.861918e+00 | 0.0451897 |
| KGZ | men | 1.033392e+00 | 9.201873e-01 | 1.154048e+00 | 0.0575366 |
| KGZ | women | 1.019332e+00 | 8.944801e-01 | 1.152769e+00 | 0.0647225 |
| KGZ | children | 1.060021e+00 | 7.536534e-01 | 1.342994e+00 | 0.1460420 |
| KGZ | elderly | 1.297232e+00 | 1.105398e+00 | 1.493438e+00 | 0.0766613 |
| LSO | men | 1.010852e+00 | 9.263644e-01 | 1.101422e+00 | 0.0439900 |
| LSO | women | 9.992451e-01 | 8.956028e-01 | 1.109863e+00 | 0.0539591 |
| LSO | children | 1.038281e+00 | 7.744667e-01 | 1.285143e+00 | 0.1309482 |
| LSO | elderly | 1.225958e+00 | 1.081702e+00 | 1.374302e+00 | 0.0610089 |
| LBR | men | 8.351083e-01 | 7.318541e-01 | 9.463800e-01 | 0.0662268 |
| LBR | women | 1.337110e+00 | 1.210855e+00 | 1.477033e+00 | 0.0504430 |
| LBR | children | 2.032738e+00 | 1.862616e+00 | 2.220550e+00 | 0.0443652 |
| LBR | elderly | 1.755939e+00 | 1.510203e+00 | 2.002534e+00 | 0.0715266 |
| MWI | men | 1.534511e+00 | 1.199224e+00 | 2.015969e+00 | 0.1326296 |
| MWI | women | 2.497918e-01 | 1.391800e-03 | 5.969550e-01 | 1.4991289 |
| MWI | children | 5.468000e-03 | 4.647000e-03 | 5.139829e-01 | 1.6831378 |
| MWI | elderly | 1.844736e+00 | 1.187221e+00 | 2.636526e+00 | 0.2039618 |
| MNG | men | 1.257279e+00 | 7.345181e-01 | 2.162088e+00 | 0.2726589 |
| MNG | women | 7.137662e-01 | 1.496807e-01 | 1.483691e+00 | 0.7038411 |
| MNG | children | 1.754440e-02 | 1.274690e-02 | 2.759240e-02 | 0.2053710 |
| MNG | elderly | 1.494329e+00 | 4.724860e-02 | 3.277424e+00 | 0.8570947 |
| MMR | men | 1.061325e+00 | 1.020939e+00 | 1.102387e+00 | 0.0194487 |
| MMR | women | 1.060742e+00 | 1.012176e+00 | 1.108461e+00 | 0.0232382 |
| MMR | children | 2.128624e+00 | 2.064252e+00 | 2.195362e+00 | 0.0157120 |
| MMR | elderly | 1.419964e+00 | 1.352353e+00 | 1.488525e+00 | 0.0245341 |
| NAM | men | 9.753666e-01 | 8.095270e-01 | 1.166815e+00 | 0.0936731 |
| NAM | women | 1.064649e+00 | 8.633918e-01 | 1.285497e+00 | 0.1016483 |
| NAM | children | 2.773110e-02 | 4.716000e-03 | 4.189158e-01 | 1.7444907 |
| NAM | elderly | 1.072356e+00 | 6.479664e-01 | 1.509509e+00 | 0.2150461 |
| NPL | men | 1.153839e+00 | 1.038729e+00 | 1.282789e+00 | 0.0536260 |
| NPL | women | 1.078744e+00 | 9.433265e-01 | 1.227046e+00 | 0.0671187 |
| NPL | children | 2.186500e-03 | 2.011800e-03 | 1.503520e-02 | 0.5506817 |
| NPL | elderly | 1.789510e+00 | 1.601906e+00 | 1.996224e+00 | 0.0562578 |
| NGA | men |  |  |  |  |
| NGA | women |  |  |  |  |
| NGA | children | 2.762353e+04 | 2.447995e+04 | 3.070231e+04 | 0.0577780 |
| NGA | elderly |  |  |  |  |
| PAK | men | 1.093061e+00 | 1.072304e+00 | 1.114775e+00 | 0.0099439 |
| PAK | women | 1.028330e+00 | 1.006678e+00 | 1.049349e+00 | 0.0106586 |
| PAK | children | 1.523971e+00 | 1.490532e+00 | 1.556594e+00 | 0.0109873 |
| PAK | elderly | 1.483934e+00 | 1.449548e+00 | 1.518920e+00 | 0.0119261 |
| PER | men | 1.147700e+00 | 1.087772e+00 | 1.211240e+00 | 0.0278942 |
| PER | women | 9.429502e-01 | 8.727436e-01 | 1.015790e+00 | 0.0389231 |
| PER | children | 1.118726e+00 | 9.449784e-01 | 1.279852e+00 | 0.0779012 |
| PER | elderly | 1.524757e+00 | 1.422869e+00 | 1.630718e+00 | 0.0346661 |
| PHL | men | 9.884333e-01 | 9.835886e-01 | 9.933821e-01 | 0.0025084 |
| PHL | women | 1.078733e+00 | 1.073021e+00 | 1.084435e+00 | 0.0026819 |
| PHL | children | 1.317005e+00 | 1.310259e+00 | 1.323784e+00 | 0.0026210 |
| PHL | elderly | 1.115657e+00 | 1.108294e+00 | 1.122876e+00 | 0.0033356 |
| MDA | men | 9.605597e-01 | 8.630048e-01 | 1.066169e+00 | 0.0544578 |
| MDA | women | 1.204476e+00 | 1.068065e+00 | 1.350488e+00 | 0.0594214 |
| MDA | children | 1.300455e+00 | 9.636368e-01 | 1.601503e+00 | 0.1295934 |
| MDA | elderly | 1.302405e+00 | 1.077582e+00 | 1.517302e+00 | 0.0868441 |
| RUS | men | 1.001505e+00 | 9.423225e-01 | 1.063315e+00 | 0.0309732 |
| RUS | women | 1.023065e+00 | 9.431722e-01 | 1.107517e+00 | 0.0408735 |
| RUS | children | 8.405560e-01 | 6.047032e-01 | 1.067565e+00 | 0.1449381 |
| RUS | elderly | 1.155550e+00 | 1.007961e+00 | 1.304383e+00 | 0.0651408 |
| SLE | men | 9.997773e-01 | 9.140730e-01 | 1.087635e+00 | 0.0442604 |
| SLE | women | 1.107794e+00 | 1.008758e+00 | 1.212633e+00 | 0.0467501 |
| SLE | children | 3.023323e+00 | 2.852832e+00 | 3.210868e+00 | 0.0298405 |
| SLE | elderly | 1.600556e+00 | 1.415687e+00 | 1.787016e+00 | 0.0599660 |
| SOM | men | 1.115260e+00 | 8.094591e-01 | 1.541186e+00 | 0.1631936 |
| SOM | women | 4.824254e-01 | 1.460468e-01 | 8.568661e-01 | 0.5683867 |
| SOM | children | 2.162383e+00 | 1.680425e+00 | 2.861485e+00 | 0.1355322 |
| SOM | elderly | 5.499100e-03 | 4.538100e-03 | 9.704480e-02 | 0.6390826 |
| ZAF | men | 6.073673e+03 | 4.522644e+03 | 7.616791e+03 | 0.1323932 |
| ZAF | women |  |  |  |  |
| ZAF | children |  |  |  |  |
| ZAF | elderly | 2.238414e+05 | 2.219424e+05 | 2.256700e+05 | 0.0042179 |
| TJK | men | 1.128918e+00 | 1.021394e+00 | 1.251659e+00 | 0.0519725 |
| TJK | women | 8.160715e-01 | 6.981792e-01 | 9.388207e-01 | 0.0755319 |
| TJK | children | 1.445930e+00 | 1.209357e+00 | 1.680109e+00 | 0.0831249 |
| TJK | elderly | 9.235389e-01 | 6.916223e-01 | 1.145862e+00 | 0.1311484 |
| THA | men | 2.395921e+00 | 2.211708e+00 | 2.615406e+00 | 0.0426967 |
| THA | women | 2.433617e+00 | 2.236708e+00 | 2.666848e+00 | 0.0446537 |
| THA | children | 2.953137e+00 | 2.455125e+00 | 3.474020e+00 | 0.0880277 |
| THA | elderly | 6.030829e-01 | 4.506799e-01 | 7.636014e-01 | 0.1345298 |
| UKR | men | 1.034024e+00 | 9.890829e-01 | 1.081966e+00 | 0.0228552 |
| UKR | women | 9.594591e-01 | 8.993702e-01 | 1.021710e+00 | 0.0323397 |
| UKR | children | 1.308429e+00 | 1.130200e+00 | 1.482813e+00 | 0.0695838 |
| UKR | elderly | 1.149581e+00 | 1.046695e+00 | 1.248807e+00 | 0.0450825 |
| TZA | men | 1.255679e+01 | 7.655100e-03 | 1.734684e+03 | 3.3546704 |
| TZA | women | 3.202580e-02 | 2.045900e-03 | 1.363528e-01 | 1.1047915 |
| TZA | children | 5.547642e+01 | 3.243295e+00 | 5.541607e+03 | 2.9221564 |
| TZA | elderly | 7.626510e-02 | 4.865400e-03 | 3.399913e-01 | 1.1084180 |
| UZB | men | 8.863624e-01 | 7.926697e-01 | 9.893216e-01 | 0.0561977 |
| UZB | women | 1.183579e+00 | 1.075531e+00 | 1.300900e+00 | 0.0490086 |
| UZB | children | 1.008920e+00 | 8.421496e-01 | 1.178685e+00 | 0.0853631 |
| UZB | elderly | 1.160510e+00 | 1.002285e+00 | 1.322466e+00 | 0.0708210 |
| VNM | men | 5.692000e-04 | 3.421000e-04 | 1.634000e-03 | 0.4352158 |
| VNM | women | 6.679130e+00 | 3.885495e+00 | 1.986806e+01 | 0.5260317 |
| VNM | children | 1.365064e+01 | 7.305995e+00 | 4.131188e+01 | 0.5439234 |
| VNM | elderly | 7.306412e-01 | 1.554300e-03 | 3.420276e+00 | 2.3039490 |
| ZMB | men |  |  |  |  |
| ZMB | women |  |  |  |  |
| ZMB | children |  |  |  |  |
| ZMB | elderly |  |  |  |  |
| ZWE | men | 1.028717e+00 | 9.741936e-01 | 1.085173e+00 | 0.0275293 |
| ZWE | women | 1.095592e+00 | 1.032814e+00 | 1.162013e+00 | 0.0298343 |
| ZWE | children | 4.443576e-01 | 2.791920e-01 | 6.019062e-01 | 0.1975437 |
| ZWE | elderly | 1.509386e+00 | 1.395402e+00 | 1.620852e+00 | 0.0382639 |

```
ga<-plotFun(resa)
# print(ga$ridgePlot)
# Prep data for comparison to children meta-analysis
resa1<-as.data.frame(resa$compDat)
resac1<-resa1[resa1$group=="children",]
resac1$Region<-dat[year=="2020",g_whoregion]
resac1$Country<-dat[year=="2020",country]
resac1$observed_A<-as.data.frame(resa$reference[,3])
resac1$expected_A<-as.data.frame(resa$reference[,4])
# Remove RR of Na
resac1<-subset(resac1,!is.na(RR))
# Conduct meta-analysis
Risk_children<-metagen(studlab=Country,sm="RR",TE=log(RR),subgroup=Region,fixed=FALSE,data=resac1,backtransf=TRUE,lower=log(RR_low),upper=log(RR_upp))
# Countries with evidence of effect
AC_evidence_hi_strong<-sum(exp(Risk_children$TE)>1.1&(Risk_children$pval<0.01))
AC_evidence_hi_med<-sum(exp(Risk_children$TE)>1.1&Risk_children$pval>0.01&Risk_children$pval<0.05)
AC_evidence_hi_weak<-sum(exp(Risk_children$TE)>1.25&Risk_children$pval>0.05&Risk_children$pval<0.1)
AC_evidence_lo_strong<-sum(exp(Risk_children$TE)<1/1.1&(Risk_children$pval<0.01))
AC_evidence_lo_med<-sum(exp(Risk_children$TE)<1/1.1&Risk_children$pval>0.01&Risk_children$pval<0.05)
AC_evidence_lo_weak<-sum(exp(Risk_children$TE)<1/1.25&Risk_children$pval>0.05&Risk_children$pval<0.1)
# P-value for regional subgroups
#Risk_children$pval.random.w
print("Meta-analysis for children/adults")
```

```
## [1] "Meta-analysis for children/adults"
```

```
Risk_children
```

```
## Number of studies combined: k = 42
## 
##                          RR           95%-CI    z p-value
## Random effects model 1.0876 [0.4062; 2.9120] 0.17  0.8672
## 
## Quantifying heterogeneity:
##  tau^2 = 10.3452 [6.9616; 17.2250]; tau = 3.2164 [2.6385; 4.1503]
##  I^2 = 99.9%; H = 31.41 [30.65; 32.19]
## 
## Test of heterogeneity:
##         Q d.f. p-value
##  40443.56   41       0
## 
## Results for subgroups (random effects model):
##                                         k     RR            95%-CI   tau^2
## Region = European Region                9 1.3250 [1.0432;  1.6830]  0.1037
## Region = South-East Asia Region         7 0.3125 [0.0327;  2.9861]  9.2356
## Region = African Region                18 2.5985 [0.4051; 16.6659] 15.6553
## Region = Region of the Americas         2 1.4008 [0.9068;  2.1637]  0.0938
## Region = Western Pacific Region         4 0.1120 [0.0013;  9.4285] 20.3942
## Region = Eastern Mediterranean Region   2 1.7684 [1.2599;  2.4821]  0.0519
##                                          tau        Q   I^2
## Region = European Region              0.3221    56.13 85.7%
## Region = South-East Asia Region       3.0390  2134.86 99.7%
## Region = African Region               3.9567 24984.67 99.9%
## Region = Region of the Americas       0.3062    21.03 95.2%
## Region = Western Pacific Region       4.5160  2276.17 99.9%
## Region = Eastern Mediterranean Region 0.2279     6.60 84.8%
## 
## Test for subgroup differences (random effects model):
##                     Q d.f. p-value
## Between groups   5.31    5  0.3789
## 
## Details on meta-analytical method:
## - Inverse variance method
## - Restricted maximum-likelihood estimator for tau^2
## - Q-profile method for confidence interval of tau^2 and tau
```

```
# Sensitivity analysis removing countries with more cases than expected
resac1s<-resac1[resac1$moreObsThanExp=="no"&(resac1$expected_A>resac1$observed_A),]
Risk_childrenS<-metagen(studlab=Country,sm="RR",TE=log(RR),subgroup=Region,fixed=FALSE,data=resac1s,backtransf=TRUE,lower=log(RR_low),upper=log(RR_upp))
print("Sensitivity analysis for children/adults removing countries with more cases than expected")
```

```
## [1] "Sensitivity analysis for children/adults removing countries with more cases than expected"
```

```
Risk_childrenS
```

```
## Number of studies combined: k = 32
## 
##                          RR           95%-CI    z p-value
## Random effects model 1.5713 [1.2174; 2.0280] 3.47  0.0005
## 
## Quantifying heterogeneity:
##  tau^2 = 0.4701 [0.3693; 1.6553]; tau = 0.6857 [0.6077; 1.2866]
##  I^2 = 99.6% [99.5%; 99.6%]; H = 15.19 [14.49; 15.94]
## 
## Test of heterogeneity:
##        Q d.f. p-value
##  7155.54   31       0
## 
## Results for subgroups (random effects model):
##                                         k     RR            95%-CI  tau^2
## Region = European Region                9 1.3250 [1.0432;  1.6830] 0.1037
## Region = South-East Asia Region         5 1.8085 [1.3481;  2.4262] 0.1107
## Region = African Region                12 1.3639 [0.6543;  2.8430] 1.4432
## Region = Region of the Americas         2 1.4008 [0.9068;  2.1637] 0.0938
## Region = Eastern Mediterranean Region   2 1.7684 [1.2599;  2.4821] 0.0519
## Region = Western Pacific Region         2 4.0666 [0.4117; 40.1637] 2.6364
##                                          tau       Q   I^2
## Region = European Region              0.3221   56.13 85.7%
## Region = South-East Asia Region       0.3328 1523.90 99.7%
## Region = African Region               1.2013  310.89 96.5%
## Region = Region of the Americas       0.3062   21.03 95.2%
## Region = Eastern Mediterranean Region 0.2279    6.60 84.8%
## Region = Western Pacific Region       1.6237   27.99 96.4%
## 
## Test for subgroup differences (random effects model):
##                     Q d.f. p-value
## Between groups   4.28    5  0.5092
## 
## Details on meta-analytical method:
## - Inverse variance method
## - Restricted maximum-likelihood estimator for tau^2
## - Q-profile method for confidence interval of tau^2 and tau
```

```
# Sensitivity analysis removing countries with poor fit or limited data
resac1p<-resac1[!resac1$iso3%in%c("AZE","BRA","CMR","COD","CAF","COG","PRK","GAB","GNB","LBR","SLE","KEN","LSO","MWI","MNG","MMR","NPL","NGA","PAK","PHL","SOM","TJK","THA","UKR","VNM","ZMB"),]
Risk_childrenP<-metagen(studlab=Country,sm="RR",TE=log(RR),subgroup=Region,fixed=FALSE,data=resac1p,backtransf=TRUE,lower=log(RR_low),upper=log(RR_upp))
print("Sensitivity analysis for children/adults removing countries with a poor fit or limited data")
```

```
## [1] "Sensitivity analysis for children/adults removing countries with a poor fit or limited data"
```

```
Risk_childrenP
```

```
## Number of studies combined: k = 17
## 
##                          RR           95%-CI     z p-value
## Random effects model 0.6534 [0.2114; 2.0197] -0.74  0.4599
## 
## Quantifying heterogeneity:
##  tau^2 = 5.2962 [2.8354; 13.2897]; tau = 2.3014 [1.6839; 3.6455]
##  I^2 = 99.5% [99.5%; 99.6%]; H = 14.61 [13.64; 15.64]
## 
## Test of heterogeneity:
##        Q d.f. p-value
##  3412.97   16       0
## 
## Results for subgroups (random effects model):
##                                   k     RR           95%-CI  tau^2    tau
## Region = South-East Asia Region   3 1.4709 [1.1862; 1.8239] 0.0360 0.1897
## Region = European Region          6 1.1939 [0.8313; 1.7146] 0.1504 0.3878
## Region = African Region           6 0.8315 [0.1114; 6.2032] 5.4874 2.3425
## Region = Western Pacific Region   1 0.0005 [0.0004; 0.0007]     --     --
## Region = Region of the Americas   1 1.1187 [0.9613; 1.3019]     --     --
##                                       Q   I^2
## Region = South-East Asia Region 1268.21 99.8%
## Region = European Region          21.47 76.7%
## Region = African Region          159.08 96.9%
## Region = Western Pacific Region    0.00    --
## Region = Region of the Americas    0.00    --
## 
## Test for subgroup differences (random effects model):
##                        Q d.f. p-value
## Between groups   1577.25    4       0
## 
## Details on meta-analytical method:
## - Inverse variance method
## - Restricted maximum-likelihood estimator for tau^2
## - Q-profile method for confidence interval of tau^2 and tau
```

```
# Prep data for comparison to elderly meta-analysis
resae1<-resa1[resa1$group=="elderly",]
resae1$Region<-dat[year=="2020",g_whoregion]
resae1$Country<-dat[year=="2020",country]
resae1$observed_A<-as.data.frame(resa$reference[,3])
resae1$expected_A<-as.data.frame(resa$reference[,4])
# Remove RR of Na
resae1<-subset(resae1,!is.na(RR))
# Conduct meta-analysis
Risk_elderly<-metagen(studlab=Country,sm="RR",TE=log(RR),subgroup=Region,fixed=FALSE,data=resae1,backtransf=TRUE,lower=log(RR_low),upper=log(RR_upp))
# Countries with evidence of effect
AE_evidence_hi_strong<-sum(exp(Risk_elderly$TE)>1.1&(Risk_elderly$pval<0.01))
AE_evidence_hi_med<-sum(exp(Risk_elderly$TE)>1.1&Risk_elderly$pval>0.01&Risk_elderly$pval<0.05)
AE_evidence_hi_weak<-sum(exp(Risk_elderly$TE)>1.25&Risk_elderly$pval>0.05&Risk_elderly$pval<0.1)
AE_evidence_lo_strong<-sum(exp(Risk_elderly$TE)<1/1.1&(Risk_elderly$pval<0.01))
AE_evidence_lo_med<-sum(exp(Risk_elderly$TE)<1/1.1&Risk_elderly$pval>0.01&Risk_elderly$pval<0.05)
AE_evidence_lo_weak<-sum(exp(Risk_elderly$TE)<1/1.25&Risk_elderly$pval>0.05&Risk_elderly$pval<0.1)
# P-value for regional subgroups
#Risk_elderly$pval.random.w
print("Meta-analysis for elderly/adults")
```

```
## [1] "Meta-analysis for elderly/adults"
```

```
Risk_elderly
```

```
## Number of studies combined: k = 41
## 
##                          RR           95%-CI    z p-value
## Random effects model 1.4036 [0.6226; 3.1643] 0.82  0.4137
## 
## Quantifying heterogeneity:
##  tau^2 = 6.8181 [4.5375; 11.4171]; tau = 2.6111 [2.1301; 3.3789]
##  I^2 = 100.0%; H = 395.22 [393.91; 396.54]
## 
## Test of heterogeneity:
##           Q d.f. p-value
##  6248047.29   40       0
## 
## Results for subgroups (random effects model):
##                                         k     RR            95%-CI   tau^2
## Region = European Region                9 1.3312 [1.1281;  1.5709]  0.0554
## Region = South-East Asia Region         7 0.5815 [0.1369;  2.4702]  3.7993
## Region = African Region                17 2.9039 [0.5051; 16.6949] 13.2053
## Region = Region of the Americas         2 1.2990 [0.9433;  1.7888]  0.0511
## Region = Western Pacific Region         4 1.1805 [1.0570;  1.3184]  0.0063
## Region = Eastern Mediterranean Region   2 0.0954 [0.0004; 22.9895] 15.3627
##                                          tau         Q    I^2
## Region = European Region              0.2354     51.29  84.4%
## Region = South-East Asia Region       1.9492   1327.15  99.5%
## Region = African Region               3.6339 286543.88 100.0%
## Region = Region of the Americas       0.2261     24.33  95.9%
## Region = Western Pacific Region       0.0794     89.06  96.6%
## Region = Eastern Mediterranean Region 3.9195     51.32  98.1%
## 
## Test for subgroup differences (random effects model):
##                     Q d.f. p-value
## Between groups   4.31    5  0.5056
## 
## Details on meta-analytical method:
## - Inverse variance method
## - Restricted maximum-likelihood estimator for tau^2
## - Q-profile method for confidence interval of tau^2 and tau
```

```
# Sensitivity analysis removing countries with more cases than expected
resae1s<-resae1[resae1$moreObsThanExp=="no"&(resae1$expected_A>resae1$observed_A),]
Risk_elderlyS<-metagen(studlab=Country,sm="RR",TE=log(RR),subgroup=Region,fixed=FALSE,data=resae1s,backtransf=TRUE,lower=log(RR_low),upper=log(RR_upp))
print("Sensitivity analysis for elderly/adults removing countries with more cases than expected")
```

```
## [1] "Sensitivity analysis for elderly/adults removing countries with more cases than expected"
```

```
Risk_elderlyS
```

```
## Number of studies combined: k = 35
## 
##                          RR           95%-CI    z  p-value
## Random effects model 1.3594 [1.2508; 1.4775] 7.22 < 0.0001
## 
## Quantifying heterogeneity:
##  tau^2 = 0.0470 [0.0293; 0.1264]; tau = 0.2168 [0.1711; 0.3555]
##  I^2 = 98.7% [98.5%; 98.8%]; H = 8.66 [8.10; 9.26]
## 
## Test of heterogeneity:
##        Q d.f. p-value
##  2548.12   34       0
## 
## Results for subgroups (random effects model):
##                                         k     RR           95%-CI  tau^2    tau
## Region = European Region                9 1.3312 [1.1281; 1.5709] 0.0554 0.2354
## Region = South-East Asia Region         6 1.2164 [0.9221; 1.6048] 0.1165 0.3414
## Region = African Region                13 1.5325 [1.3898; 1.6898] 0.0143 0.1197
## Region = Region of the Americas         2 1.2990 [0.9433; 1.7888] 0.0511 0.2261
## Region = Western Pacific Region         4 1.1805 [1.0570; 1.3184] 0.0063 0.0794
## Region = Eastern Mediterranean Region   1 1.4839 [1.4497; 1.5190]     --     --
##                                            Q   I^2
## Region = European Region               51.29 84.4%
## Region = South-East Asia Region       957.78 99.5%
## Region = African Region                36.70 67.3%
## Region = Region of the Americas        24.33 95.9%
## Region = Western Pacific Region        89.06 96.6%
## Region = Eastern Mediterranean Region   0.00    --
## 
## Test for subgroup differences (random effects model):
##                      Q d.f. p-value
## Between groups   20.18    5  0.0012
## 
## Details on meta-analytical method:
## - Inverse variance method
## - Restricted maximum-likelihood estimator for tau^2
## - Q-profile method for confidence interval of tau^2 and tau
```

```
# Sensitivity analysis removing countries with poor fit or limited data
resae1p<-resae1[!resae1$iso3%in%c("CMR","CAF","COD","LBR","SLE","PRK","GAB","GNB","KEN","IND","MWI","MNG","MMR","NAM","NPL","NGA","PAK","MDA","SOM","ZAF","UZB","VNM","ZMB","ZWE"),]
Risk_elderlyP<-metagen(studlab=Country,sm="RR",TE=log(RR),subgroup=Region,fixed=FALSE,data=resae1p,backtransf=TRUE,lower=log(RR_low),upper=log(RR_upp))
print("Sensitivity analysis for elderly/adults removing countries with a poor fit or limited data")
```

```
## [1] "Sensitivity analysis for elderly/adults removing countries with a poor fit or limited data"
```

```
Risk_elderlyP
```

```
## Number of studies combined: k = 20
## 
##                          RR           95%-CI    z p-value
## Random effects model 1.2375 [1.0792; 1.4190] 3.05  0.0023
## 
## Quantifying heterogeneity:
##  tau^2 = 0.0727 [0.2591; 3.5088]; tau = 0.2696 [0.5090; 1.8732]
##  I^2 = 99.0% [98.8%; 99.2%]; H = 10.01 [9.23; 10.85]
## 
## Test of heterogeneity:
##        Q d.f. p-value
##  1902.14   19       0
## 
## Results for subgroups (random effects model):
##                                   k     RR           95%-CI  tau^2    tau
## Region = European Region          7 1.3661 [1.1001; 1.6964] 0.0757 0.2752
## Region = South-East Asia Region   3 1.0322 [0.6236; 1.7086] 0.1926 0.4389
## Region = African Region           6 0.4474 [0.0590; 3.3928] 5.6347 2.3738
## Region = Region of the Americas   2 1.2990 [0.9433; 1.7888] 0.0511 0.2261
## Region = Western Pacific Region   2 1.1802 [1.0559; 1.3191] 0.0064 0.0798
##                                      Q   I^2
## Region = European Region         49.84 88.0%
## Region = South-East Asia Region 174.70 98.9%
## Region = African Region          35.59 86.0%
## Region = Region of the Americas  24.33 95.9%
## Region = Western Pacific Region  88.94 98.9%
## 
## Test for subgroup differences (random effects model):
##                     Q d.f. p-value
## Between groups   2.89    4  0.5762
## 
## Details on meta-analytical method:
## - Inverse variance method
## - Restricted maximum-likelihood estimator for tau^2
## - Q-profile method for confidence interval of tau^2 and tau
```

# 10. Plot missing cases

We plot country-level observed vs expected cases

```
# Observed vs expected notifications for men
options(ggrepel.max.overlaps = 12)
resmm1 <- merge(as.data.frame(resm$reference),resm1[c("iso3","Region")],by="iso3")
plot_Mmissing<-ggplot(data=resmm1,aes(x=log10(observed),y=log10(expected),color=Region,label=iso3))+
  geom_text_repel(aes(label=iso3))+
  geom_point(size=4)+
  geom_abline(intercept=0,slope=1,linetype="dashed",colour="black",size=0.5)+
  geom_hline(yintercept=0, linetype="solid", colour="black", size=0.1)+
  geom_vline(xintercept=0, linetype="solid", colour="black", size=0.1)+
  scale_colour_manual(values=c("#79ACE7","#E8E863","#E179E7","#E77979","#9EE779","#7BE7CF"),guide="none")+
  scale_y_continuous(trans='log10',name="Expected men",breaks=c(3,4,5,6),labels=c("1,000","10,000","100,000","1,000,000"))+
  scale_x_continuous(trans='log10',name="Observed men",breaks=c(3,4,5,6),labels=c("1,000","10,000","100,000","1,000,000"))+
  coord_cartesian(xlim =c(3,6.5),ylim =c(3,6.5))+
  theme(axis.text.x=element_text(size=10),axis.text.y=element_text(size=10,angle=90,hjust=0.5),axis.title.x=element_text(size=12),axis.title.y=element_text(size=12),legend.position=c(0.8,0.3),legend.background = element_blank(),legend.text = element_text(size=6),legend.title = element_text(size=9))
plot_Mmissing
```

```
ggsave("Missing_M.png",device="png",width=10,height=10,units=c("cm"))
# Observed vs expected notifications for women
options(ggrepel.max.overlaps = 12)
plot_Wmissing<-ggplot(data=resm1,aes(x=log10(observed),y=log10(expected),color=Region,label=iso3))+
  geom_text_repel(aes(label=iso3))+
  geom_point(size=4)+
  geom_abline(intercept=0,slope=1,linetype="dashed",colour="black",size=0.5)+
  geom_hline(yintercept=0, linetype="solid", colour="black", size=0.1)+
  geom_vline(xintercept=0, linetype="solid", colour="black", size=0.1)+
  scale_colour_manual(values=c("#79ACE7","#E8E863","#E179E7","#E77979","#9EE779","#7BE7CF"))+
  scale_y_continuous(trans='log10',name="Expected women",breaks=c(2,4,6),labels=c("100","10,000","1,000,000"))+
  scale_x_continuous(trans='log10',name="Observed women",breaks=c(2,4,6),labels=c("100","10,000","1,000,000"))+
  coord_cartesian(xlim =c(2,6.5),ylim =c(2,6.5))+
  theme(axis.text.x=element_text(size=10),axis.text.y=element_text(size=10,angle=90,hjust=0.5),axis.title.x=element_text(size=12),axis.title.y=element_text(size=12),legend.position=c(0.8,0.3),legend.background = element_blank(),legend.text = element_text(size=6),legend.title = element_text(size=9))
plot_Wmissing
```

```
ggsave("Missing_W.png",device="png",width=10,height=10,units=c("cm"))
# Observed vs expected notifications for children
options(ggrepel.max.overlaps = 20)
plot_Cmissing<-ggplot(data=resac1,aes(x=log10(observed),y=log10(expected),color=Region,label=iso3))+
  geom_text_repel(aes(label=iso3))+
  geom_point(size=4)+
  geom_abline(intercept=0,slope=1,linetype="dashed",colour="black",size=0.5)+
  scale_colour_manual(values=c("#79ACE7","#E8E863","#E179E7","#E77979","#9EE779","#7BE7CF"),guide="none")+
  scale_y_continuous(trans='log10',name="Expected children",breaks=c(1,3,5),labels=c("10","1,000","100,000"))+
  scale_x_continuous(trans='log10',name="Observed children",breaks=c(1,3,5),labels=c("10","1,000","100,000"))+
  coord_cartesian(xlim =c(0.5,5.5),ylim =c(0.5,5.5))+
  theme(axis.text.x=element_text(size=10),axis.text.y=element_text(size=10,angle=90,hjust=0.5),axis.title.x=element_text(size=12),axis.title.y=element_text(size=12),legend.position=c(0.8,0.3),legend.background = element_blank(),legend.text = element_text(size=6),legend.title = element_text(size=9))
plot_Cmissing
```

```
ggsave("Missing_C.png",device="png",width=10,height=10,units=c("cm"))
# Observed vs expected notifications for elderly
options(ggrepel.max.overlaps = 15)
plot_Emissing<-ggplot(data=resae1,aes(x=log10(observed),y=log10(expected),color=Region,label=iso3))+
  geom_text_repel(aes(label=iso3))+
  geom_point(size=4)+
  geom_abline(intercept=0,slope=1,linetype="dashed",colour="black",size=0.5)+
  geom_hline(yintercept=0, linetype="solid", colour="black", size=0.1)+
  geom_vline(xintercept=0, linetype="solid", colour="black", size=0.1)+
  scale_colour_manual(values=c("#79ACE7","#E8E863","#E179E7","#E77979","#9EE779","#7BE7CF"),guide="none")+
  scale_y_continuous(trans='log10',name="Expected elderly",breaks=c(2,3,4,5),labels=c("100","1,000","10,000","100,000"))+
  scale_x_continuous(trans='log10',name="Observed elderly",breaks=c(2,3,4,5),labels=c("100","1,000","10,000","100,000"))+
  coord_cartesian(xlim =c(1.5,5.5),ylim =c(1.5,5.5))+
  theme(axis.text.x=element_text(size=10),axis.text.y=element_text(size=10,angle=90,hjust=0.5),axis.title.x=element_text(size=12),axis.title.y=element_text(size=12),legend.position=c(0.8,0.3),legend.background = element_blank(),legend.text = element_text(size=6),legend.title = element_text(size=9))
plot_Emissing
```

```
ggsave("Missing_E.png",device="png",width=10,height=10,units=c("cm"))
# Observed vs expected notifications for adults
resaa1 <- merge(as.data.frame(resa$reference),resac1[c("iso3","Region")],by="iso3")
options(ggrepel.max.overlaps = 15)
plot_Amissing<-ggplot(data=resaa1,aes(x=log10(observed),y=log10(expected),color=Region,label=iso3))+
  geom_text_repel(aes(label=iso3))+
  geom_point(size=4)+
  geom_abline(intercept=0,slope=1,linetype="dashed",colour="black",size=0.5)+
  geom_hline(yintercept=0, linetype="solid", colour="black", size=0.1)+
  geom_vline(xintercept=0, linetype="solid", colour="black", size=0.1)+
  scale_colour_manual(values=c("#79ACE7","#E8E863","#E179E7","#E77979","#9EE779","#7BE7CF"),guide="none")+
  scale_y_continuous(trans='log10',name="Expected adults",breaks=c(3,4,5,6),labels=c("1,000","10,000","100,000","1,000,000"))+
  scale_x_continuous(trans='log10',name="Observed adults",breaks=c(3,4,5,6),labels=c("1,000","10,000","100,000","1,000,000"))+
  coord_cartesian(xlim =c(3,6.5),ylim =c(3,6.5))+
  theme(axis.text.x=element_text(size=10),axis.text.y=element_text(size=10,angle=90,hjust=0.5),axis.title.x=element_text(size=12),axis.title.y=element_text(size=12),legend.position=c(0.8,0.3),legend.background = element_blank(),legend.text = element_text(size=6),legend.title = element_text(size=9))
plot_Amissing
```

```
ggsave("Missing_A.png",device="png",width=10,height=10,units=c("cm"))
```

# 11. Plot meta-analyses

We plot forest plots for the meta-analyses

```
# Men to women comparison
png("forest_MF.png", width = 1000, height = 1800, res=120) 
forest(x=Risk_sex, print.subgroup.name=FALSE,text.random="Overall summary",text.random.w="Regional summary",ref=1,at=(c(.1, 1, 10)),
       leftcols=c("studlab"),rightcols=c("effect","ci"),weight.study="same",weight.subgroup="same",test.subgroup.random=FALSE,xlim=c(0.1,10),
       lty.random = 0,hetstat=FALSE,sortvar=-TE,leftlabs = c("Country"),smlab=("Women:Men"),rightlabs = c("Risk ratio","95% CI"),
       col.study="black",col.diamond = "white",col.square="black",col.inside="black",squaresize=0.5,col.by="black",allstudies=FALSE)
dev.off()
```

```
## png 
##   2
```

```
# Adults to children comparison
png("forest_AC.png", width = 1000, height = 1800, res=120) 
forest(x=Risk_children, backtransf=TRUE,print.subgroup.name=FALSE,text.random="Overall summary",text.random.w="Regional summary",ref=1,at=(c(.1, 1, 10)),
       leftcols=c("studlab"),rightcols=c("effect","ci"),weight.study="same",weight.subgroup="same",test.subgroup.random=FALSE,xlim=c(0.1,10),
       lty.random = 0,hetstat=FALSE,sortvar=-TE,leftlabs = c("Country"),smlab=("Children:Adults"),rightlabs = c("Risk ratio","95% CI"),
       col.study="black",col.diamond = "white",col.square="black",col.inside="black",squaresize=0.5,col.by="black",allstudies=FALSE)
dev.off()
```

```
## png 
##   2
```

```
# Adults to elderly comparison
png("forest_AE.png", width = 1000, height = 1800, res=120) 
forest(x=Risk_elderly, backtransf=TRUE,print.subgroup.name=FALSE,text.random="Overall summary",text.random.w="Regional summary",ref=1,at=(c(.1, 1, 10)),
       leftcols=c("studlab"),rightcols=c("effect","ci"),weight.study="same",weight.subgroup="same",test.subgroup.random=FALSE,xlim=c(0.1,10),
       lty.random = 0,hetstat=FALSE,sortvar=-TE,leftlabs = c("Country"),smlab=("Elderly:Adults"),rightlabs = c("Risk ratio","95% CI"),
       col.study="black",col.diamond = "white",col.square="black",col.inside="black",squaresize=0.5,col.by="black",allstudies=FALSE)
dev.off()
```

```
## png 
##   2
```
